# Tri-objective generator maintenance scheduling model based on sequential strategy

**DOI:** 10.1371/journal.pone.0276225

**Published:** 2022-10-18

**Authors:** Shatha Abdulhadi Muthana, Ku Ruhana Ku-Mahamud

**Affiliations:** 1 General Company of South Electricity Distribution, Ministry of Electricity, Baghdad, Iraq; 2 School of Computing, Universiti Utara Malaysia, Kedah, Malaysia; 3 Shibaura Institute of Technology, Tokyo, Japan; Karlstad University: Karlstads Universitet, SWEDEN

## Abstract

A multi-objective modeling approach is required in the context of generator maintenance scheduling (GMS) for power generation systems. Most multi-objective modeling approaches in practice are modeled using a periodic system approach that caters for a fixed maintenance window. This approach is not suitable for different types of generating units and cannot extend the generator lifespan. To address this issue, this study proposes a tri-objective GMS model with three conflicting objectives based on the sequential system approach that accounts for operating hours and start-up times. The GMS model’s objectives are to minimize the total operation cost, maximize system reliability and minimize violation. The main difference between the proposed tri-objective GMS model and other multi-objective GMS models, is that the proposed model uses a sequential strategy based on operating hours and start-up times. In addition, the proposed model has considered the most important criteria in scheduling the generator maintenance, and this reflects the real-life requirements in electrical power systems. A multi-objective graph model is also developed to generate the maintenance units scheduling and used in developing the proposed Pareto ant colony system (PACS) algorithm. A PACS algorithm is proposed to implement the model and obtain solution for GMS. The performance of the proposed model was evaluated using the IEEE RTS 26, 32, and 36-unit systems dataset. The performance metrics used comprise the GMS model objectives. The experimental results showed that the obtained solution from the proposed tri-objective GMS model was a robust solution by considering the different initial operational hours of the units.

## I. Introduction

In the electricity industry there are two types of power systems known as regulated (centralized) and deregulated (restructured) power systems. The traditional system is a regulated power system with a central control structure that fully regulates the activities of production, transmission, and distribution systems; in other words, a single operator controls all operations of the power system and is responsible for centrally solving all the system problems, including maintenance scheduling problems [1–3]. However, towards the ends of 1990s, many places had replaced these monopolies due to competition [1–3]. Many countries have privatized their own electricity industries where they dismantled the integrated energy system into three major sectors known as generating, transmission, and distribution companies. This concept is called restructured power systems or deregulated power systems. According to the studies of [2, 3], the main aim of regulated systems is to improve on the reliability while lowering costs. The main aim of deregulated power systems is to maximize the profits of generating companies while also organizing the decisions of the different entities. It can be observed that regulated power systems’ objectives are relevant to the cost and reliability criteria. This may conflict with the objectives of deregulated power systems which focus on profit. As a result, the focus of this study has been on generator maintenance scheduling in regulated power systems because the main trend for many countries is to provide a reliable supply of electricity at the lowest possible cost.

In some power systems, generating units representing gas turbines play a prime role in supplying energy [4]. A gas turbine is a repairable, degraded system, and preventive maintenance generally improves a fraction of its overall performance. A study by [5] suggests that a gas turbine requires more frequent inspection and maintenance as it ages. Therefore, periodic maintenance scheduling is ineffective for this type of generator and a maintenance strategy based on a sequential approach is required. Thus, the generator maintenance scheduling (GMS) problem is more suitably solved using a sequential system approach. Before the scheduling process, there is the planning process. The planning process determines the scheduling. In this study, planning for the scheduling depends on the units’ operational hours in addition to the number of periods of maintenance scheduling over one year (i.e., 52 periods). However, if a unit is to enter maintenance at period 52, the maintenance will prolong to period 53. It is assumed, in this study, that the maintenance duration is two periods (i.e., two weeks) and is identical for all units in the generated test systems. The GMS problem has been traditionally formulated as a single optimization problem which is mostly integrated within the dominant scheduling criteria in the objective function. However, some of these criteria are claimed to cause constraints by some studies [3]. Therefore, these problems require the multi-objective modeling approach to be applied. In this study, a tri-objective GMS model is proposed to fulfill the requirements of a real electrical power system. A graph model has to be developed to generate the scheduling of the maintenance units. The single objective unidirectional graph models in the studies of [4, 6–8] are used to show the process of developing the generators’ maintenance schedule in the single objective scenario. No graph model has previously been proposed from extant multi-objective GMS studies [9–12].

The methods used in solving single objective problems are called single objective optimization methods (SOOMs). Some of these methods include exact algorithms, heuristic, metaheuristic, and hybrid metaheuristic search algorithms while the methods used to solve multi-objective problems are known as multi-objective optimization methods (MOOMs) [13]. Studies by [14, 15] assert that MOOMs are alternative methods that can be applied to solve the complex problems of GMS with the support of non-Pareto or Pareto-based techniques. Different criteria can be used to classify the technique as either non-Pareto-based or Pareto-based. The basic idea of Pareto-based techniques is that Pareto front directly incorporates the concept of Pareto optimum. Non-Pareto-based techniques are approaches that do not directly incorporate the concept of Pareto optimum [16]. In addition, [17] give classification to algorithms depending on whether the output of the algorithms is a set of Pareto solutions (i.e., a set of optimal solutions) or the output is a single solution. The Pareto dominance approach uses Pareto dominance in relation to selecting non-dominated solutions. Solutions for non–Pareto-based techniques, as suggested by [14], include a vector optimized evolutionary strategy, vector evaluated genetic algorithm, and weight-based genetic algorithm. Meanwhile, for Pareto-based techniques such as Pareto based ant colony optimization algorithms [18–20], strength Pareto evolutionary algorithm, multi-objective genetic algorithm, and non-dominated sorting genetic algorithm [14] are suggested. Other algorithms that apply the Pareto-based-technique include a dominance based multi-objective simulated annealing algorithm [10], and multi-objective differential evolution algorithm [12]. Studies by [14, 21], support that the Pareto-based technique is mostly used as a quality criterion to find several best solutions. In general, for electric energy optimization, ant colony optimization algorithms have shown superiority and relevance in solving generator or power plant maintenance scheduling problems [4, 6–8, 21]. The findings of [22] express one of the prominent intelligent algorithms, i.e., the ant colony system (ACS), which is implemented widely to solve various types of scheduling problems. Most importantly, a study by [4] discloses that obtaining a solution by the ACS algorithm for GMS with a systems approach based on operational hours, is extremely powerful with various initializations of hours. In addition, the ACS algorithm is very effectively used when the system operator prefers to update the maintenance scheduling program. However, the ACS algorithm can be used to obtain a solution from a single objective GMS model, and if this is to be used with the proposed model, it needs some adjustments. Thus, in this study, the Pareto ant colony system (PACS) algorithm is proposed to obtain a solution from the proposed tri-objective GMS model. As the Pareto front contains a vast number of non-dominated solutions, choosing one solution from a large set is not easy for the decision maker [16]. Methods such as the Grey Relational Analysis, Technique for Order Preference by Similarity to Ideal Solution and Simple Additive Weighting are preferred when choosing one of the non-dominated solutions [23]. In this study Grey Relational Analysis is used to select one solution from the Pareto front.

Section II reviews previous studies on GMS models and optimization approaches. This is followed by Section III which describes the proposed tri-objective GMS model. In Section IV a multi-objective graph model is presented, and Section V describes the proposed PACS algorithm. Experimental design of the proposed model is presented in Section VI while Section VII presents the discussions on the presented results. Finally, Section VIII depicts the conclusions and future work.

## II. Related literature

The GMS is a complex and non-linear optimization problem that specifies the maintenance schedule of power generation units [24]. Various GMS models have been developed to represent single and multi-objective electrical power system requirements to generate the schedule for unit maintenance [24]. Any mathematical GMS model can consist of decision variables, parameters, constraints, and objective functions. According to [3, 25], the criteria that are commonly used in developing GMS models are reliability, cost, and convenience. Most importantly [2, 10] emphasize that the criteria that draw the attention of many studies in developing GMS models comprise reliability and cost, especially in regulated power systems, and profit for deregulated power systems. In addition, there is an environmental criterion, which has recently received attention when solving GMS problems [26, 27]. Risk is another criterion that is also considered [28, 29]. These criteria can be considered as objective functions or constraints. If the criteria in a GMS model are considered as objective functions that are not aggregated, then it is a multi-objective GMS model, and the solution can be optimized using the multi-objective optimization method. The single objective optimization method can be used to optimize a solution with a single criterion or multi-criteria that aggregate the objective functions or consider one of the criteria as an objective function and the others as constraints [11].

It is almost impossible to solve the GMS problem that represents multiple requirements of a real power system by a single criterion. If a multi criteria is considered by making one of the criteria an objective function and the remaining as constraints, then the solution only fulfills part of the requirements. This is insufficient to regulate the relative importance of the requirements of the company. In addition, if a multi-criteria is considered by aggregate multi-objective functions, the disadvantage of the aggregated method is that the weight values are not easy to obtain, and they need to be finely tuned in different runs to obtain a Pareto front [11, 14]. Thus, considering multi-criteria as objective functions without aggregation will have to be used to meet the diverse needs of any realistic power system. However, the maintenance strategy used in most of the models uses a fixed maintenance window, which is classified as a periodic maintenance strategy. This strategy is inefficient for different types of generating units and it cannot extend the lifespan of generating units [4]. Thus, the sequential strategy is considered more efficient because it depends on the age of the generating units which can extend the life of the units. In addition, the strategy can be applied with different types of generating units. This strategy is implemented in [4] which considers operational hours in deciding the maintenance outage of units. The sequential strategy is also implemented in [30] but in a different way. The age is calculated as a proportion of the time (i.e., year) the unit has been in the system. However, this does not reflect the actual working time of the units. The method adopted in [4] in deciding the age reflects that the exact working hours of the generating unit is better. However, [4] consider operating hours without start-up times; the inventors have discovered that, generally, one engine start is equivalent to 10 hours of operation in terms of the impact on the life of the engine [31]. Thus, maintenance outages of generating units have to be considered based on two factors, operating hours and start-up times, particularly for gas turbine generating units.

Several studies consider the multi criteria by a single objective function which considers one of the criteria as an objective function and the other as a constraint. The cost objective function is explored in [32] by minimizing the expected energy production cost and presenting reliability by the stochastic constraints of unserved amounts of electrical power. Convenience criteria are also considered for treating complex constraints in GMS problems. In [33] minimization of the whole operating risk during the midterm horizon is considered as the objective function, which consists of individual operating risk and system operating risk. Operating risk includes the sum of the expected corrective maintenance costs which is calculated by the product of the expected failure number of the system, while system operating risk includes reliability criteria that incorporate expected energy not supplied. Moreover, the cost criterion is considered through a constraint of maximum prospective preventive maintenance cost limitation. Profit as an objective function is suggested by [34, 35], and reliability is fulfilled through various kinds of constraints such as the reserve capacity of the system and the load satisfying constraints. In addition, [35] consider reliability in terms of stochastics through forced outage constraint to reduce the damage in power plants. Moreover [34] consider convenience criteria where constraint violation is treated by adding the penalty factor to the objective function.

The cost objective function proposed by [36] includes generating and maintenance costs with peak regulation pressure penalty fee. Furthermore, [36] propose reliability criteria through two constraints, i.e., deterministic by satisfying the reserve capacity of the corresponding period, and stochastic by constraint of expected energy not supplied. Cost as an objective function is proposed by [37] considering maintenance and generation costs. Reliability is considered through the load balance constraint that ensures the provision of a sufficient power supply. [37] consider the convenience criterion where constraints violation is treated by adding the penalty factor to the objective function. According to [29], the scheduling objective seeks to maximize the probability of no power generating units failing during the scheduling window (i.e., minimizing the risk of generating units failing). Additionally, reliability is considered through power demand satisfaction constraints for the system. In soft constraints if they are violated by a candidate solution, the solution is still considered feasible, but the objective function is then penalized according to the number of such constraint violations and the degree of each violation, thus convenience criteria are also considered. [4] consider the cost criterion through the objective of total operational cost minimization, and reliability considered through system reserve constraints. However, a multi-criteria model by considering one of the criteria as an objective function and others as constraints is insufficient to meet all the requirements of the real power system. The solution only fulfils part of the requirements, and this is insufficient to regulate the relative importance of the requirements of the company.

Multi-criteria can be solved by aggregating multi-objective functions. However, the weight values are not easy to obtain, and need to be finely tuned in different runs to obtain a Pareto front [11, 14]. These studies include [38] who propose a cost objective by minimizing the total operating cost, and a reliability objective that is achieved in two ways; minimizing the sum of squares of reserve and minimizing the loss of load expectation. [38] also consider convenience by handling the constraints’ violations using the penalty function method. [27] establish a GMS model by setting generation costs minimization, maximization reliability in terms of being deterministic through the supply reserve rate. Reliability is also considered in terms of stochastics through minimization of loss of load expectation. In addition [27] consider environmental criteria through minimizing CO_2_ emissions. [28] consider three objectives, i.e., reliability maximization by minimizing the sum of squared reserves, minimizing operational cost consisting of maintenance cost, start-up cost and cost of generation and, lastly, minimizing the risk of generating unit fails before scheduled maintenance periods. [39] consider two objectives including expected total maintenance cost and risk criteria which are considered by lowering failure intensity.

The GMS model that considers multi-criteria by several objective functions, more realistically represents an actual power system. Thus, considering multi-criteria as objective functions without aggregation will have to be used to meet the diverse needs of any realistic power system. [11] consider, in their study, three types of objective functions, comprising producer profit, system reliability which is defined as the minimization of the standard deviation of the reliability index, and total generation cost. [10] consider, in their model, two objective functions, those of maximizing reliability by minimizing the sum of squared reserves and minimizing cost through electricity production cost. A multiplicative penalty function is employed when any constraint violation occurs, thus a convenience criterion is also considered. The objectives considered by [12] include minimizing the overall operational cost and maximizing the deterministic reliability of the power system by maximizing the average value of reliability index in the planning period. [30] incorporate two objectives in their model comprising maximizing reliability through maximizing the expected rate of energy and minimizing cost through minimizing the total expected costs related to maintenance efforts. [40] consider cost minimization through maintenance cost; furthermore, reliability is considered by providing sufficient power reserve and the sustainability effects to the wind farm system. However, although these studies consider multi criteria with multi objective functions, most adopt the periodic strategy for maintenance scheduling since this strategy is convenient to implement. However, the sequential strategy is more appropriate when the system requires frequent maintenance as it ages [4, 5, 31]. Thus, the periodic strategy is not suitable for systems such as gas turbine generating units, that consist of generators requiring frequent maintenance because some parts in the generator will expire after certain operating hours and, therefore, must be replaced with new parts.

A sequential strategy is adopted by [4, 30, 39], implement sequential strategy by defining the age of the unit as equal to a proportion of the time the unit has been in the system, while [4] consider operational hours in deciding the unit’s maintenance outage. The method adopted by [4] in deciding the age reflects the exact working hours of the generating unit. Thus, the method of [4] is adapted in this study to consider a maintenance sequential strategy based on operational hours which include start-up times in addition to operating hours that can extend the life of generating units. The periodic strategy, adopted by other studies, can be used if the age of the generator is not considered.

The scope of this study focuses on GMS model with multi-objective functions in electrical power systems, i.e., cost, reliability, and convenience, in addition to a sequential maintenance strategy based on operating hours and start-up times. Table 1 illustrates the criteria and maintenance strategies of several GMS models, in addition to the solution methods that are considered to implement the models and obtain solutions for the GMS. It can be summarized that the most popular criteria used in GMS are reliability, cost, and convenience. In addition, the periodic maintenance strategy has been commonly adopted because it is more convenient than a sequential maintenance strategy. However, the periodic strategy cannot be applied to different types of generating units like gas turbines. The metaheuristics optimization method is a prominent algorithm that has proved efficient to produce optimal or near optimal solutions.

**Table 1:** GMS models and solution methods.

Reference	Criteria’s type	GMS models’ criteria						Strategy of maintenance				Solution method
		Reliability	Cost	Convenience	Environmental	Profit	risk	Sequential			Periodic	
								Years	Operating hours	Start-up times		
[32]	Multi criteria, single objective optimization method	√	√	√	x	x	x	x	x	x	√	Genetic algorithm
[33]		√	√	x	x	x	√	x	x	x	√	Decomposition algorithm based on genetic algorithm
[34]		√	x	√	x	√	x	x	x	x	√	Hybrid differential evolution algorithm
[35]		√	x	x	x	√	x	x	x	x	√	Modified electro search optimization algorithm
[36]		√	√	√	x	x	x	x	x	x	√	Genetic algorithm
[37]		√	√	√	x	x	x	x	x	x	√	Mathematical approach assisted differential evolution algorithm
[29]		√	x	√	x	x	√	x	x	x	√	Exact solution approach via CPLEX
[4]		√	√	x	x	x	x	x	√	x	x	Ant colony system algorithm
[38]		√	√	√	x	x	x	x	x	x	√	fuzzy supported teaching learning algorithm
[27]		√	√	x	√	x	x	x	x	x	√	Genetic algorithm
[28]		√	√	x	x	x	√	x	x	x	√	Exchange market algorithm
[39]		x	√	x	x	x	√	√	x	x	x	Genetic algorithm
[11]	Multi criteria, multi-objective optimization method	√	√	x	x	√	x	x	x	x	√	Group search optimizer with multiple producers’ algorithm
[10]		√	√	√	x	x	x	x	x	x	√	Dominance-based multi objective simulated annealing algorithm
[12]		√	√	x	x	x	x	x	x	x	√	Fuzzy clustered multi objective hybrid differential evolution algorithm
[30]		√	√	x	x	x	x	√	x	x	x	Multi-objective particle swarm optimization algorithm
[40]		√	√	x	x	x	x	x	x	x	√	Non dominated sorting genetic algorithm II

### III. Proposed tri-objective GMS model

The proposed tri-objective GMS model comprises four main components: decision variables, parameters, constraints, and objectives. The four decision variables are *X*_*ij*_, *Y*_*ij*_, *V*_*ijt*_, and *y*_*ijt*_. There are twenty-seven parameters, including *LI*, *LJ*, *LT*, *pend*_*i*,*j*_ (*age*)*, NUW, NUS, ${oph}_{i}^{s},{oph}_{i}^{maxav},{oph}_{i}^{minrq},N_{i},D_{i},p_{ijt},,\Gamma_{i}^{down}$, G,* and *g(j)*. Constraints consist of several generator maintenance scheduling and unit commitment constraints. In this research, there are eight constraints which are classified as either hard or soft constraints. The hard constraints are load balance, minimum system reserve, and minimum and maximum capacity of generating unit. The soft constraints are the maintenance outage unit constraint, continuous maintenance constraint, minimum up and downtime constraint, maintenance and online status constraint. The maintenance window constraint in some cases is considered hard, while in other cases is considered soft. If the unit’s operational hour is at the upper end point of the maintenance window, this constraint is considered hard. However, if it is at the lower end point, this constraint is considered soft. The final component of the model is the objectives and, in this study, there are three main objectives. The first objective is the most common economic criterion for regulated power systems, which focuses on the minimization of the total operation cost. The total cost of operation includes both energy production cost and maintenance cost. The second objective is the maximization of reliability, which focuses on maximization of the system’s gross reserve to achieve a more reliable margin of generating capacity above the expected demand. Finally, the third objective is the convenience objective, which focuses on minimization of the violation in maintenance outage unit constraint. This objective aims to have more units available for production. These components are addressed at length in the latter part of this section. In the proposed GMS model, the unit commitment within the hourly time scale is considered with GMS, and the outages of units are scheduled in such a way that the units’ operating hours without maintenance inspections are between the pre-determined minimum and maximum operating hours of the units. Thus, this problem is more complex than periodic scheduling of maintenance because scheduling maintenance requires an analysis of the cost, gross reserve, and violation in each time interval. As a result, the cost, gross reserve and violation are analyzed based on the decision of hours.

In this study, the GMS and unit commitment problems were solved simultaneously in a long-term planning horizon (i.e., one year). The unit commitment problem plays a key part in designing and operating electrical power systems. The power generation industry uses unit commitment and economic dispatch to help with generation scheduling decisions. Unit commitment determines the optimum combination of generating units at the power system with lowest production cost for a given planning horizon (a day or a week in advance). The set of operating constraints should be considered in unit commitment. The economic dispatch problem seeks to define the optimal production of obtainable generation units to meet the predictable demand at the lowest probable cost, given a number of constraints. According to [3], the economic dispatch problem is typically modeled as a sub-problem of the unit commitment problem. Further, the study by [3] illustrates that the unit commitment problem deals with how many units to commit over a time period whereas the economic dispatch problem deals with how much power/energy each power generating unit that has been committed should deliver. In addition [41] disclose that in maintenance scheduling with economic dispatch, the main challenge is to efficiently and optimally schedule generating units for maintenance while the running units handle fluctuating, uncertain and peak loads throughout the maintenance period.

The study of [4] considers the GMS model based on the operating hours sequential approach. However, the start-up times with operating hours in deciding the maintenance outages of generating was not considered. Furthermore, the inherent trade-offs between the various conflicting scheduling objectives were not addressed. As a result, with this deficiency, the GMS resolution is far from the optimum solution. The proposed tri-objective GMS model is developed based on the model of [4] with three enhancements. The first enhancement is the use of start-up times with operating hours for deciding the units’ maintenance outage as oppose to [4] who only used operating hours. This is because one engine start is equivalent to 10 hours of operation in terms of the impact on the life of the engine. The second enhancement is assigning the unit’s maintenance outage constraint as a soft instead of hard constraint as assigned in [4]. This approach to enhance flexibility in an abnormal situation reflects a more realistic situation. More units will be in operation to meet the higher demand and, thus, there is a possibility for more units to be sent for maintenance when the units’ working hours are close to the upper endpoint of the maintenance window. Hence, if the abnormal situations are considered, this constraint has to be soft, then the number of feasible solutions can be increased which results in more optimal solutions. The third enhancement is to enable the model to consider three objectives instead of a single objective. This will reflect a more realistic model of the electrical power system. The following sections describe the components of the proposed tri-objective GMS model.

### A. GMS model decision variables

The relationship between decision variables and maintenance schedule in a power system can be defined as follows: suppose *I* = {1,…,*LI*} set of generating units, indexed by *i*, *J* = {1,…,*LJ*} set of periods (weeks) indexed by *j* and *k*, *T* = {1,…,*LT*} set of hourly times, indexed by *t* (168h in a week), then a maintenance schedule is an assignment of zeros and ones for decision variables. The listing of GMS decision variables is shown in Table 2.

**Table 2:** Decision variables for generator maintenance scheduling.

Symbol	Meaning
**Indices:**	
*i*	Index of generating units.
*j*, *k*	Indexes of periods (weeks).
*t*	Index of hours.
**Sets:**	
*I*	Set of generating units.
*J*	Set of periods (weeks).
*T*	Set of hours.
**Variables:**	
X*X*_*ij*_	Binary variable shows the maintenance status of unit *i*∈*I* at period *j*∈*J* (1 if the unit *i* is on maintenance, otherwise is 0).
*Y*_*ij*_	Binary variable shows the starting maintenance status of unit *i*∈*I* at period *j*∈*J* (1 if the maintenance outage of unit *i* is started in period *j*, otherwise is 0).
V*V*_*ijt*_	Binary variable shows the online status of unit *i*∈*I* at period *j*∈*J*, and hour *t*∈*T* (1 if unit *i* is online at period *j* and hour *t*, otherwise is 0).
*y*_*ijt*_	Binary variable shows the start-up status of unit *i* at period *j* and hour *t* (1 if unit *i* is started-up at period *j* and hour *t*, otherwise is 0).

The GMS problem is usually presented as a scheduling problem with binary decision variables which represent whether different units should be maintained over each set of periods. However, another type of decision variable can be considered to show the online and start-up status of units as shown in Table 2. Hence, these decision variables are also important to show the status of other units.

### B. GMS model parameters

The inputs that are required to configure the tri-objective GMS model are parameters presented in Table 3. The majority of these inputs are usually required when solving instances of GMS problems. These data serve as the GMS system’s benchmark, which is critical when configuring a GMS model in an electrical power system.

**Table 3:** Parameters and description.

Parameters	Meaning
*LI*	Total number of units.
*LJ*	Total number of periods.
*LT*	Total hours in each period (168 h in a week).
*pend*_*i*,*j*_ (*age*)	Operational hours of unit *i* at the beginning of period *j* after the last maintenance outage, if the outage is started in period *j*; otherwise, it equals to zero.
*NUW*	Number of units working hours, since entering service initially or after an overhaul.
*NUS*	Number of starts for the unit, since entering service initially or after an overhaul.
${oph}_{i}^{s}$	Initial operational hours of unit *i* before starting scheduling and after performing the last maintenance outage.
${oph}_{i}^{maxav}$	Maximum available operational hours for unit *i* between the last maintenance outage and the next one.
${oph}_{i}^{minrq}$	Minimum required operational hours for unit *i* between the last maintenance outage and the next one.
*N*_*i*_	Maximum number of units for maintenance outages in period *j*.
*D*_*i*_	Duration (periods) of the maintenance outage for unit *i*.
*p*_*ijt*_	Power generation dispatch of unit *i* in period *j* and time *t*.
*D*_*jt*_	System demand in period *j* and time *t*.
$R_{jt}^{min}$	Minimum reserve requirement in period *j* and time *t*.
$P_{i}^{min}$	Minimum capacity of unit *i*.
$P_{i}^{max}$	Maximum capacity of unit *i*.
*G*_*ijt*_	Power generating capacity of unit *i* during period *j* and time *t*.
$C_{i}^{s}$	Start-up cost of unit *i*.
$C_{i}^{Fx}$	Fixed cost ($/h) of online unit *i*.
$C_{i}^{P}$	Production cost ($/MWh) of unit *i*.
$C_{i}^{M}$	Maintenance cost ($) of unit *i*.
$s_{it}^{on}$	Continuous on-span of unit *i*, after which the unit *i* is switched off.
$s_{it}^{off}$	Continuous off-span of unit *i*, after which the unit *i* is switched on.
$\Gamma_{i}^{up}$	Known minimum up time of unit *i*.
$\Gamma_{i}^{down}$	Known minimum down time of unit *i*.
*G*	Limiting value of soft constraint.
*g(j)*	Violation value of soft constraint in period *j*.

### C. GMS model constraints

In general, constraint is a condition that must be satisfied in any optimization solution. The constraints that have been used in this study to configure the tri-objective GMS model are the generator maintenance scheduling and unit commitment in addition to coupling constraints between unit commitment and GMS. Generator maintenance scheduling constraints that have been considered are the maintenance outage units, and continuous maintenance, whilst unit commitment constraints are load balance, minimum system reserve, minimum and maximum capacity of generating units, and minimum up and down time constraints. However, coupling constraints are the participation constraints in an activity of maintenance scheduling between unit commitment and GMS which are maintenance and online status as well as maintenance window. The constraints are divided into hard and soft constraints. In an optimization model, a hard constraint must be satisfied by any feasible solution to the model. A soft constraint on the other hand can be violated. To limit the occurrence of violations, the convenience objective function is considered for the soft constraint.

#### 1) Maintenance window constraints

For maintenance scheduling based on operating hours, the constraints of minimum and maximum operational hours for maintenance outage of each unit are presented in Eq (1) and Eq (2) [4]. The constraint in Eq (1) ensures that the maintenance outage of unit *i* can start at period *j*, if its operating hours after the last outage at the beginning of period *j* are more than the lower endpoint of its maintenance window.


$${pend}_{i,j}\geq {oph}_{i}^{minrq}\;\forall i\in I,j\in J$$ (1)

Further, the constraint in Eq (2) enforces unit *i* to start the maintenance outage at period *j*, if its respective operating hours from the previous outage could exceed the upper endpoint of the maintenance window by being online in period *j*.


$${pend}_{i,j}\geq {oph}_{i}^{maxav}-LT\;\forall i\in I,j\in J$$ (2)

In other words, as supported by [4], the maintenance window constraints are to check the minimum and maximum operational hours for maintenance outage of each unit.

The generating units of gas turbines have an age and the closer the age is to the predefined maximum number of hours leads to the increase in the probability of maintenance outage of the generating unit. However, the age of the generating unit has not exceeded the predefined maximum number of hours. The age of a gas turbines generating unit can be determined in two ways. According to [5], the first way assumes that the system ages only when it is in operation. Further, [5] explain that the second way is to define the age based on the number of working hours and the number of times the unit has started operation since entering the service. In addition [31] state that the inventors have discovered that, generally, one engine start is equivalent to 10 hours of operation in terms of the impact on the life of the engine. Thus, maintenance outages of generating units must be considered based on two factors: start-up times and operating hours, particularly for gas turbine generating units. Although, this may increase the possibility of maintenance costs and decrease the system’s gross reserve because it will expedite the process of entering generating units into maintenance outages; nevertheless, it will in return increase the lifespan of generating units. Thus, it will avoid the numerous failures that may arise in the future. Hence, in this study the *pend*_*i*,*j*_ (age) that determines the maintenance outages of unit *i* at period *j* is calculated as in Eq ((3) [31].


$${pend}_{i,j}=NUW+(NUS*10)\forall i\in I,j\epsilon J$$ (3)

#### 2) Maintenance outage unit’s constraint

This constraint is included to indicate the number of units for maintenance outage in a specific period, *j*, which is presented in Eq (4) [4]. The probability of sending units for maintenance outage is increased when its working hours’ time is close to the upper endpoint of the maintenance window. In the proposed model, for normal situations, a specific number which indicates the maximum number of units can be sent for maintenance. This constraint is considered a soft constraint to enhance flexibility in an abnormal situation which reflects a more realistic situation. More units will be in operation to meet the increased demand and, thus, there is a possibility of more units being sent for maintenance when the units’ working hours are close to the upper endpoint of the maintenance window. Hence, if abnormal situations are taken into consideration, this constraint must be soft, as the number of feasible solutions can be increased, resulting in more optimal solutions.


$$\sum_{i=1}^{LI}X_{ij}\leq N_{i},\forall \;j\epsilon J$$ (4)

#### 3) Continuous maintenance constraint

This constraint guarantees that when the maintenance outage is started in the period, *j*, it will continue for the required number of periods which is presented in Eq (5) [4, 10].


$$\sum_{k=j}^{k=j+D_{i}-1}X_{ik}\geq D_{i}Y_{ij},\forall \;i\epsilon I,j\epsilon J$$ (5)

#### 4) Load balance constraint

This constraint ensures that the load demand equals the total power production of online units during each period and hour which is presented in Eq (6) [4].


$$\sum_{i=1}^{LI}{V_{ijt}p}_{ijt}=D_{jt},\forall \;j\epsilon J,t\epsilon T$$ (6)

#### 5) Minimum system reserve constraint

This constraint guarantees that the total maximum productions for online units exceed the load demand plus the reserve, presented in Eq (7) [4].


$$\sum_{i=1}^{LI}V_{ijt}P_{i}^{max}\geq D_{jt}+R_{jt}^{min},\forall \;j\epsilon J,t\epsilon T$$ (7)

#### 6) Minimum and maximum capacity of generating unit’s constraint

This constraint controls the upper and lower bounds of production for each online generating unit, presented in Eq (8) [4, 42].


$$V_{ijt}P_{i}^{min}{\leq p}_{ijt}\leq V_{ijt}P_{i}^{max}\forall i\in I,j\epsilon J,t\epsilon T$$ (8)

#### 7) Minimum up and downtime constraints

The minimum up constraint ensures that if the unit is turned on, it should remain on for a minimum predefined number of hours, i.e., a unit must be continuously ‘on’ for a certain number of time instants before it can be switched off [4, 43]. Meanwhile, the minimum downtime constraint is to ensure that, if the unit turns off, it should remain off for the minimum predefined number of hours, i.e., a unit must be continuously ‘off’ for a certain number of time instants before it can be switched on [4, 43], as presented in Eqs (9) and (10).


$$s_{it}^{on}\geq \Gamma_{i}^{up}\forall \;i\epsilon I,t\epsilon T$$ (9)

$$s_{it}^{off}\geq \Gamma_{i}^{down}\forall \;i\epsilon I,t\epsilon T$$ (10)

#### 8) Maintenance and online status constraint

This constraint prevents any unit undergoing maintenance from being online (i.e., not connected again to the network during specified period *j* and time *t*), as presented in Eq (11) [4].


$$X_{i,j}+V_{i,j,t}\leq 1\;\forall i\in I,j\in J,T\in t$$ (11)

### D. GMS model objectives

The proposed model consists of three main objectives namely cost, reliability, and convenience [3]. The cost objective is proposed to minimize the total operation cost which includes production and maintenance costs, while the reliability objective is proposed to maximize reliability by considering gross reserve for creation of an extra reliable margin of generating capacity above the expected demand. The convenience objective is used to overcome the difficulties in fulfilling all of the constraints and to limit the violation. Sometimes constraints’ violations are allowed [3] as minimization of these soft constraint violations is incorporated as a separate GMS objective (called a convenience objective). Hence, in this study this objective was used to limit the violations of a maintenance outage unit’s constraint. Thus, if the search space increases, the number of feasible solutions can be increased, and more optimal solutions can be obtained.

#### 1) Operation cost GMS model objective

There are two different types of costs, known as the production and maintenance costs [4]. The function for cost has two parts; the first part of the cost objective refers to the resulting cost from online units (i.e., production cost), while the second part refers to the resulting cost from units that are on maintenance outage (i.e., maintenance cost). The function for the operation cost objective is presented in Eq (12) [4].


$$minimze\;\sum_{i=1}^{LI}\sum_{j=1}^{LJ}\sum_{t=1}^{LT}(C_{i}^{Fx}V_{ijt}+{C_{i}^{s}y_{ijt}+C}_{i}^{P}p_{ijt})+\sum_{i=1}^{LI}\sum_{j=1}^{LJ}\left(C_{i}^{M}X_{ij}\right)$$ (12)

#### 2) Reliability GMS model objective

Gross reserve is used to investigate the obtained solutions in terms of system reliability, and it can be defined as the differences between the available capacity of system generators that are not on maintenance outage and the demand system [4, 44]. Generally, the aspect of gross reserve refers to the ability to create a more reliable margin of generating capacity above the expected demand, whereas demand refers to the rate at which electricity is consumed. The function for the reliability objective is presented in Eq (13).


$$maximize\;\sum_{i=1}^{LI}V_{ijt}P_{i}^{max}-D_{jt}$$ (13)

#### 3) Convenience GMS model objective

Minimizing soft constraint violations is a type of convenience objective [3]. The convenience objective is formulated in this study to limit the violation in maintenance outage unit’s constraint and to gain an optimal solution with less violation in the number of units that have to be in the maintenance outage. The function for the convenience (i.e., violation) objective is presented in Eq (14) [25], where *G* is the limiting value of soft constraint, and *g(j)* is the number of violations for soft constraint in each period *j*.


$$minimze\;\sum_{j=1}^{LJ}\max\left\{0,g(j)-G\right\}$$ (14)

## IV. Generating maintenance scheduling graph model

**Fig 1:**
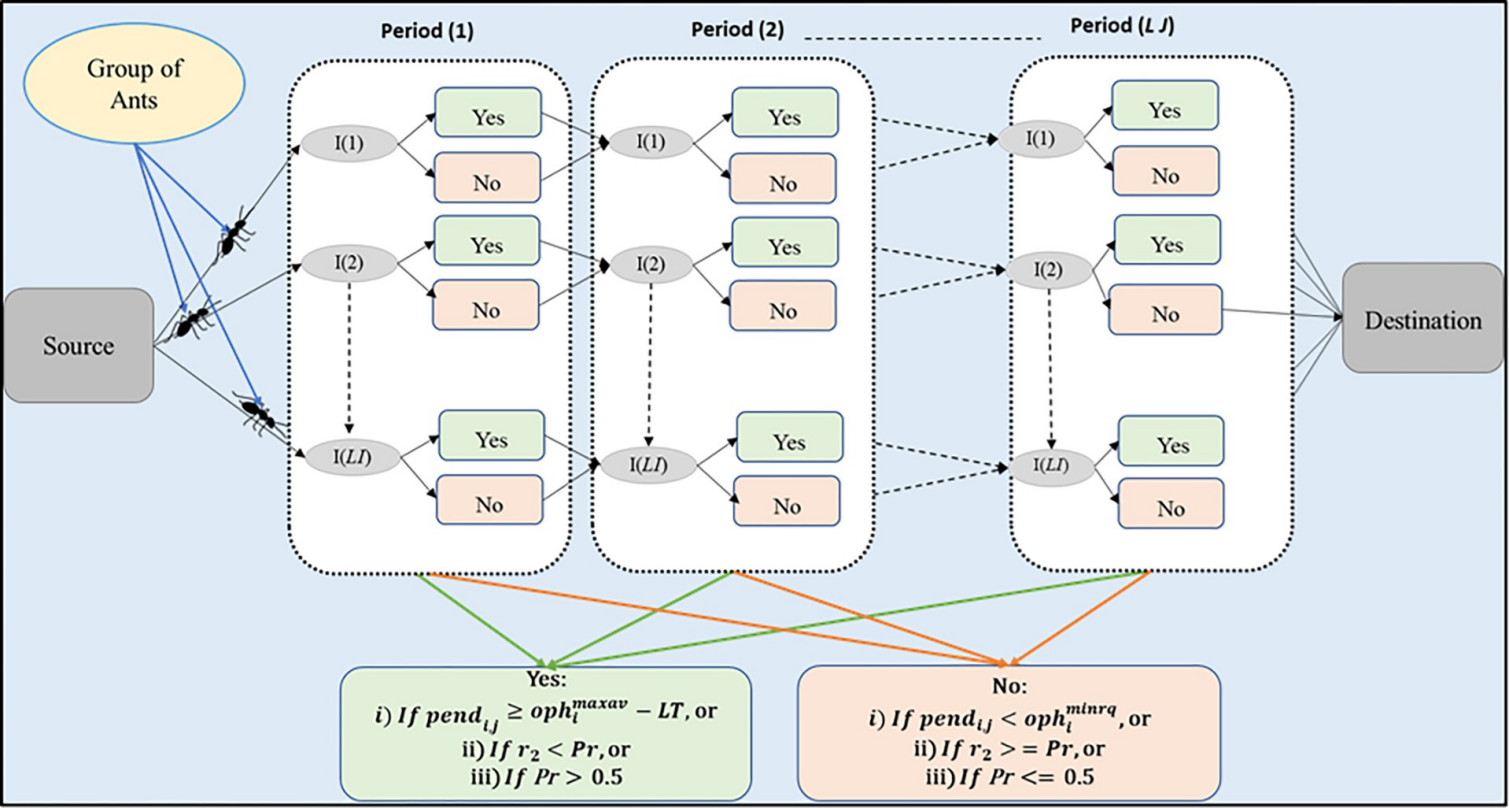
Graph model for maintenance scheduling of generating units using ants’ group movement.

A unidirectional multi-objective graph model is developed based on the single objective graph model presented in [4] for scheduling the maintenance of generating units for one year. In addition, the graph model is used in developing the multi-objective PACS algorithm. Enhancement in the decision of units’ maintenance outage in [4] was made. The proposed graph model presented in Fig 1 considers the decisions of *Yes* or *No* to determine the units’ maintenance outage in each period. The decision is made by looking initially to the operational hours (*pend*_*i*,*j*_) of the unit, if it is at the lower or upper endpoint of the maintenance window, then PACS algorithm rules will be considered if it is not at the upper endpoint of the maintenance window. In this study, the enhancement in the decision has considered start-up times in the calculation of the unit’s operational hours beside operating hours. Furthermore, in terms of the algorithm, this includes enhancement in calculating pheromone and heuristic information values. Three (3) pheromone values are defined using one pheromone for each objective. The heuristic information of each unit is also determined based on operational hours, and the lower or upper end points of the maintenance window. In contrast, [4] consider the ACS rules with one pheromone for one objective. Furthermore, in [4] work also determines the heuristic information based on the operational hours as well as the lower or upper end points of the maintenance window, but the calculation of the operational hours only depends on the operating hours, the start-up times of the units are not considered.

The steps of the solution construction in the graph model are as follows:

1. The first ants’ group starts by deciding if the unit’s maintenance outage should be performed or not. If *Yes* then the unit should enter maintenance outage and if *No*, the unit will not undergo maintenance outage. The decision of *Yes* or *No* can be determined by looking at the operational hours (*pend*_*i*,*j*_) of the unit. If the unit has been in operation for quite some time, i.e., the unit’s operational hours are at the upper end point of the maintenance window (${pend}_{i,j}\geq {oph}_{i}^{maxav}-LT$), the unit will enter maintenance outage during this period (i.e., ‘*Yes*’). If the operation hours are less than the minimum requirement of the operational hours (i.e., ${pend}_{i,j}<{oph}_{i}^{minrq}$), the unit will not enter the maintenance outage during this period (i.e., ‘*No*’). However, if the operational hours are between the lower and upper maintenance window endpoints (i.e., ${pend}_{i,j}\geq {oph}_{i}^{minrq}$), a further decision will have to be made for the unit either to enter the maintenance outage or not. A random number *r*_1_ between (0,1) will be generated.
  - If (*r*_1_<*Exploration rate*), then generate another random number *r*_2_ between (0,1), and calculate the probability of units’ maintenance outage using the following proposed probability (*Pr*) equation:
$$pr=\frac{{\left(\left[C\tau_{yes}^{c}]+[R\tau_{yes}^{r}]+[V\tau_{yes}^{v}\right]\right)}^{\alpha }{\left[\eta_{yes}\right]}^{\beta }}{{\left(\left[C\tau_{yes}^{c}]+[R\tau_{yes}^{r}]+[V\tau_{yes}^{v}\right]\right)}^{\alpha }{\left[\eta_{yes}\right]}^{\beta }+{\left(\left[C\tau_{no}^{c}]+[R\tau_{no}^{r}]+[V\tau_{no}^{v}\right]\right)}^{\alpha }{\left[\eta_{no}\right]}^{\beta }}$$ (15)
  - If (*r*_2_<*Pr*) then perform the unit’s maintenance outage, otherwise do not perform (i.e., *r*_2_≥*Pr*).
  - If (*r*_1_≥*Exploration rate*), then check the value of *Pr*. If *Pr* > 0.5 then perform the unit’s maintenance outage, otherwise do not perform (i.e., *Pr* ≤ 0.5). The higher the value of *Pr the* more likely it is to enter the maintenance outage.
2. Step (1) will be repeated in other periods until all units have been scheduled for maintenance outage.

After all ants have obtained the scheduling solution, the objective functions are calculated and the solution of the first ants’ group will be stored as the best solution so far. The local pheromone update will be performed before other ants’ groups construct their scheduling solutions according to the process (steps 1 and 2) of the first group. The Pareto approach is applied to make tradeoff between the obtained solutions from the groups of ants whereby the Pareto front for the optimal solution is constructed. This is performed after the second group of ants has constructed the solution. An iteration is completed when all ants’ groups have produced their solutions. The global update is then performed for the best ant groups before the second and consecutive iterations are performed. In each iteration, the Pareto front is updated based on the Pareto front of the previous iteration. The last Pareto front, when all iterations have been completed, will contain the best scheduling solution.

In Eq (15) the parameters *ƞ* and *τ* represent the heuristic and pheromone in which the values of these parameters are used to guide a solution for optimal maintenance scheduling based on operational hours with low cost, high reliability and low violation. The pheromone values are obtained from updated trail information during previous steps based on the result of the objective functions. The parameters of *C*, *R*, and *V* are regulating the relative importance of each objective. The values for *C*, *R*, and *V* are randomly assigned within the range [0, 1) while the summation of *C*, *R*, and *V* has to be equal to one [45, 46]. Table 4, displays the parameters’ values of ACS algorithms as considered by [4, 24].

**Table 4:** Parameter default values.

Factor	Global rate (*p*)	Local rate (ξ)	Initial pheromone	Pheromone power (α)	Heuristic power (β)	Exploration probability	No. of iterations	No. of ants group
Parameters	0.1	0.005	0.01	1	0.005	0.1	100	100

## V. Proposed multi-objective PACS algorithm

The development of the multi-objective PACS algorithm is based on the single objective ACS algorithm presented in [4], and the multi-objective graph model presented in this study. The single objective ACS algorithm presented in [4] adopts the sequential strategy based on operational hours, which has obtained better solutions for systems based on operational hours. The PACS algorithm is used to obtain the solution for the maintenance schedule of the generating unit for one year. Enhancements in PACS algorithm include: i) the transition rule for choosing the periods of units’ maintenance outage presented in Eq (15), ii) algorithm local update presented in Eqs (18)–(21), and iii) algorithm global update presented in Eqs (24)–(27). In addition, the Pareto approach has been considered for trade-off decision making. Furthermore, in this study the multi-objective Pareto ACS nomenclature in [4] is updated to accommodate a broader range of parameters that are required to solve the multi-objective GMS problem. The pseudo code of the proposed PACS algorithm is presented in [47]. The proof that optimal or near optimal Pareto front has been achieved is based on comparison between the proposed algorithm and four other multi-objective optimization algorithms (i.e., Non-dominated Sorting Genetic, Strength Pareto Evolutionary, Multi-objective Simulated Annealing, and Multi-objective Particle Swarm Optimization) using the performance metrics coverage, distance to Pareto front, and overall Pareto spread [47].

Table 5 displays the multi-objective Pareto ACS nomenclature with the addition of pheromone values of ants to make the decision of whether or not to undertake maintenance outage of a specific unit in a specific period. The parameters *ƞ* and *τ* represent the heuristic and pheromone respectively. The heuristic is used to determine whether to enter, or not, maintenance outage based on operational hours (i.e., operating hours and start-up times) of the units. If *YES*, then the unit should enter maintenance outage and this usually occurs when the operational hour of the unit exceeds the specified lower endpoint of the maintenance window, which increases as it approaches close to the upper endpoint of the maintenance window. Conversely, *NO* indicates not to enter the maintenance outage which is usually when the unit operational hours are far from the specified maintenance window. Similar to the heuristic, the pheromone parameter is used to make decision whether to enter the maintenance outage. However, the decision, here, will depend on the best compromise solution for cost, reliability, and convenience objective functions as opposed to operational hours.

**Table 5:** Pareto ant colony system’s nomenclature.

Parameters	Meaning
*η*_*yes*_	heuristic amount of the *i*th ant using to make a decision to do the maintenance outage for unit *i* at period *j*.
*η*_*no*_	heuristic amount of the *i*th ant using to make a decision not to do the maintenance outage for unit *i* at period *j*.
$\tau_{yes}^{c}$	pheromone value of the *i*th ant using to make a decision to do the maintenance outage for unit *i* at period *j*.
$\tau_{yes}^{r}$	pheromone value of the *i*th ant using to make a decision to do the maintenance outage for unit *i* at period *j*.
$\tau_{yes}^{v}$	pheromone value of the *i*th ant using to make a decision to do the maintenance outage for unit *i* at period *j*.
$\tau_{no}^{c}$	pheromone value of the *i*th ant using to make a decision not to do the maintenance outage for unit *i* at period *j*.
$\tau_{no}^{r}$	pheromone value of the *i*th ant using to make a decision not to do the maintenance outage for unit *i* at period *j*.
$\tau_{no}^{v}$	pheromone value of the *i*th ant using to make a decision not to do the maintenance outage for unit *i* at period *j*.

The scheduling algorithm consists of three (3) main processes. The processes include local pheromone update, global pheromone update and trade-off between the obtained solutions of the ant groups using the Pareto approach.

### A. Local pheromone updates (YES) and (NO) based on cost, reliability, and convenience objective functions

The pheromone quantity deposited by ants in period *j* is updated to reflect the experience acquired by ants. At the beginning of each period, *j* is initialized with a weak amount of pheromone $\left(\tau_{yes}^{c}=\tau_{0}),(\tau_{no}^{c}=\tau_{0}),(\tau_{yes}^{r}=\tau_{0}),(\tau_{no}^{r}=\tau_{0}\right)$ and $(\tau_{yes}^{v}=\tau_{0}),(\tau_{no}^{v}=\tau_{0}).$ During solution construction, the pheromone trails of period *j* visited by an ant can be locally updated using the following equations:

$$\tau_{yes}^{c}\leftarrow (1-\xi )\tau_{yes}^{c}+\tau_{0}$$ (16)

$$\tau_{no}^{c}\leftarrow (1-\xi )\tau_{no}^{c}+\tau_{0}$$ (17)

$$\tau_{yes}^{r}\leftarrow (1-\xi )\tau_{yes}^{r}+\tau_{0}$$ (18)

$$\tau_{no}^{r}\leftarrow (1-\xi )\tau_{no}^{r}+\tau_{0}$$ (19)

$$\tau_{yes}^{v}\leftarrow (1-\xi )\tau_{yes}^{v}+\tau_{0}$$ (20)

$$\tau_{no}^{v}\leftarrow (1-\xi )\tau_{no}^{v}+\tau_{0}$$ (21)

Where, *ξ* is the local pheromone evaporation rate of (0<*ξ*<1) utilized to avert unbounded accumulation of pheromone. Consequently, updating of the local pheromone is applied for exploring the search space’s new areas. More precisely, the impact of the local update rule is that every time ants use a period, *j*, its pheromone trails are decreased. For this reason, the node of that period is less popular to the next ants and assists to avert ants that keep track of the same path. This permits the exploration of periods previously unexplored. In practice, this has an effect on the algorithm, which does not display stagnation behavior (i.e., ants do not converge to the common path’s generation).

### B. Global pheromone updates (YES) and (NO) based on cost, reliability, and convenience objective functions

After all ants have completed their solutions, the rules of the global pheromone update are applied to the preferable set. As in updating of the local pheromone rule, *p* global is the evaporation rate (0<*p*<1) utilized to explore new areas in the search space. Global pheromone updating is applied to exploit the best solution in the search space. Therefore, for the best ants’ group the pheromone trails in period *j* visited by an ant can be globally updated using the following equations:

$$\tau_{yes}^{c}\leftarrow (1-p).\tau_{yes}^{c}+p.\Delta_{yes}^{c}$$ (22)

$$\tau_{no}^{c}\leftarrow (1-p).\tau_{no}^{c}+p.\Delta_{no}^{c}$$ (23)

where,

$$\Delta_{yes,no}^{c}=\{\frac{1}{\begin{array}{c} best\;operation\;cost\\ 0\;otherwise \end{array}}\;(if\;period\;j\in Best\;Solution)$$

$$\tau_{yes}^{r}\leftarrow (1-p).\tau_{yes}^{r}+p.\Delta_{yes}^{r}$$ (24)

$$\tau_{no}^{r}\leftarrow (1-p).\tau_{no}^{r}+p.\Delta_{no}^{r}$$ (25)

where,

$$\Delta_{yes,no}^{r}=\left\{ \begin{array}{c} 1*best\;reliability\;(if\;period\;j\in Best\;Solution)\\ 0\;otherwise \end{array}\right.$$

$$\tau_{yes}^{v}\leftarrow (1-p).\tau_{yes}^{v}+p.\Delta_{yes}^{v}$$ (26)

$$\tau_{no}^{v}\leftarrow (1-p).\tau_{no}^{v}+p.\Delta_{no}^{v}$$ (27)

where,

$$\Delta_{yes,no}^{v}=\{\frac{1}{\begin{array}{c} best\;less\;violation\\ 0\;otherwise \end{array}}(if\;period\;j\in Best\;Solution)$$

### C. Pareto approach for solution trade-off

With multi-objective optimization, solutions are linked according to the thought of dominance which supplies practitioners with multiple solutions that can be compared. The concept of optimality has been popularized to include the concept of effective solutions set, also known as Pareto frontier. The concept of dominance is formulated as follows: solution *X* is told to dominate the other solution *X*^/^ if both two conditions are true: (i) solution *X* is no worse than *X*^/^ in all objectives, and (ii) solution *X* is better than *X*^/^ in at least one objective. In this study a dominance measure’s criterion makes comparison between two solutions (*X*, *X*^/^) based on three objectives and decides which of the following conditions apply:

1. 1-If *X* is better in all objectives than *X*^/^, *X* is said to strongly dominate *X*^/^, and can be denoted as *X*≻≻*X*^/^.
2. 2- If *X* is not worse than *X*^/^ in all objectives and better in two objectives, *X* is said to dominate *X*^/^, and can be denoted as *X*≻*X*^/^.
3. 3- If *X* is not worse than *X*^/^ in all objectives and better in at least one objective, *X* is said to weakly dominate *X*^/^, and can be denoted as *X*≻*X*^/^.
4. 4- If *X* does not weakly dominate *X*^/^, nor *X*^/^ does not weakly dominate *X*, it is said they are an equal, and can be denoted as *X* = *X*^/^.

The mathematical representation of the dominance measures mentioned above for the two solutions *X*(*c*,*r*,*v*) and *X*^/^(*c*^/^,*r*^/^,*v*^/^) are:

If $\left(c<c^{/})˄(r>r^{/})˄(v<v^{/}\right)$, then *X* is said to strongly dominate *X*^/^.
2. If $\left(\left(c<c^{/})˄(r>r^{/})˄(v=v^{/}\right))˅(\left(c<c^{/})˄(r=r^{/})˄(v<v^{/}\right))˅(\left(c=c^{/})˄(r>r^{/})˄(v<v^{/}\right)\right)$, then *X* is said to dominate *X*^/^.
If $(\left(c<c^{/})˄(r=r^{/})˄(v=v^{/}\right))˅(\left(c=c^{/})˄(r>r^{/})˄(v=v^{/}\right))˅(\left(c=c^{/})˄(r=r^{/})˄(v<v^{/}\right)),$ then *X* is said to weakly dominate *X*^/^.
If $\left((c>c^{/})˄(r>r^{/})˄(v<v^{/}))˅((c<c^{/})˄(r<r^{/})˄(v<v^{/}))˅((c<c^{/})˄(r>r^{/})˄(v>v^{/}))˅((c>c^{/})˄(r>r^{/})˄(v=v^{/}))˅((c>c^{/})˄(r=r^{/})˄(v<v^{/}))˅((c<c^{/})˄(r<r^{/})˄(v=v^{/}))˅((c=c^{/})˄(r<r^{/})˄(v<v^{/}))˅((c<c^{/})˄(r=r^{/})˄(v>v^{/}))˅((c=c^{/})˄(r>r^{/})˄(v>v^{/}))˅((c>c^{/})˄(r=r^{/})˄(v=v^{/}))˅((c=c^{/})˄(r<r^{/})˄(v=v^{/}))˅((c=c^{/})˄(r=r^{/})˄(v>v^{/}))˅((c=c^{/})˄(r=r^{/})˄(v=v^{/})\right)$, then *X* is said an equal to *X*^/^.

## VI. Experimental design

**Fig 2:**
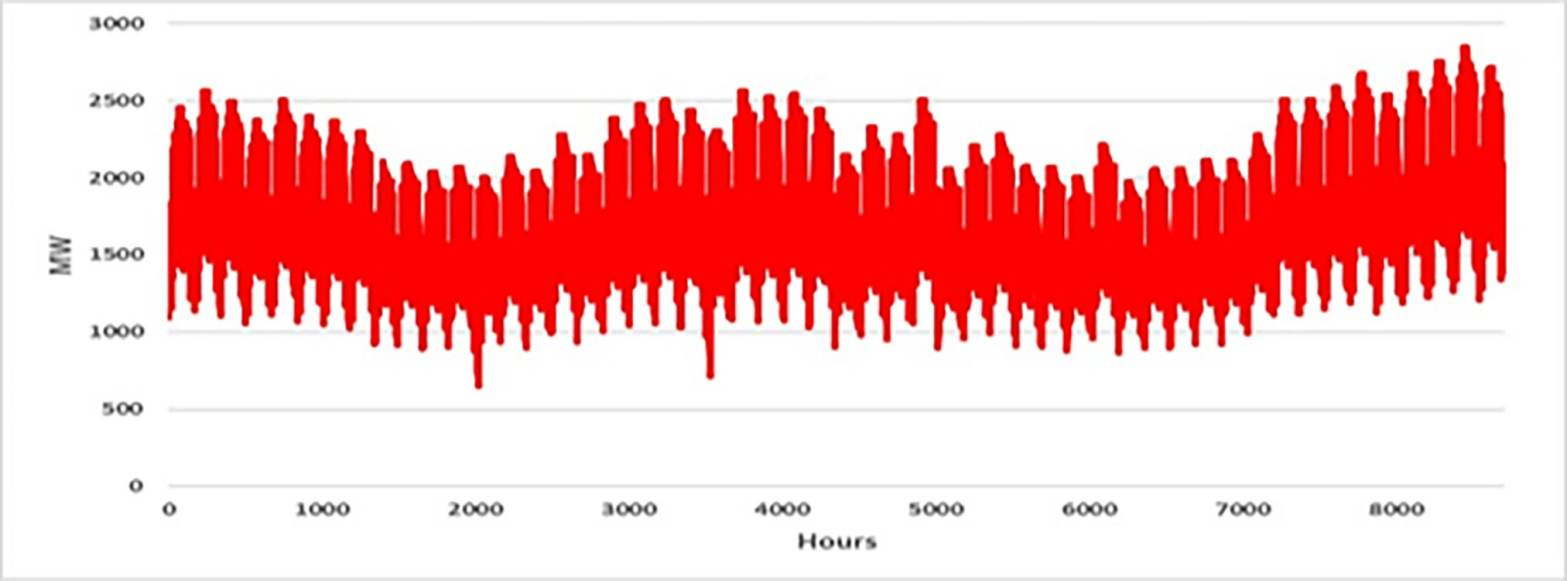
Expected hourly load demand data.

The current study takes into account the case study of a power plant with 26, 32, and 36 units. The details of this test system are based on IEEE RTS 26, 32, and 36 unit systems, which is supported by [4, 24, 48]. The test system includes six (6) maintenance windows and the demand for the 26 and 32 test systems is based on IEEE RTS demand systems [4, 24, 48]. The demand tested in the 36-unit system is double that tested in the 26- and 32-unit systems as presented in [4]. Fig 2 displays the fluctuations in load demand over the course of an hour. The maintenance duration for each unit is two weeks while the start-up cost of units is randomly generated between $100 and $200. It is also assumed that the maximum number of units for maintenance outages in period *j* is three (3) and that if there are more than three (3), violation will occur, and that the system reserve is 5% of the load demand at the time of each period and hour.

Evaluation of the proposed tri-objective GMS model is made by comparing the results of the proposed model with the single objective GMS model. In performing the comparison, the ACS algorithm is used to implement the single objective model and produce the solution for GMS as proposed by [4]. The PACS algorithm is used to implement and produce a solution for the tri-objective GMS model. This kind of comparison is based on extreme solution which, in this study, consists of three objectives: operation cost, reliability, and violation.

An extreme solution is one that has the potential to provide the best value in a specified objective regardless of other objective values [49]. The calculation for the percentage of cost improvement (*PCI*) is given in Eq (28) [4], whereas the calculation for the percentage of violation improvement (*PVI*) and percentage of reliability improvement (*PRI*) are proposed as in Eqs (29) and (30) respectively.


$$PCI=\frac{original\;number-new\;number}{original\;number}*100$$ (28)

$$PVI=\frac{original\;number-new\;number}{original\;number}*100$$ (29)

$$PRI=\frac{new\;number-original\;number}{original\;number}*100$$ (30)

The stability of the GMS model is measured using the coefficient of variation (CV) of the gross reserve as discussed in [4]. Regardless of the importance of other objectives (i.e., operation cost and convenience), gross reserve is chosen to calculate the coefficient of variation. The gross reserve is the most volatile variable that can affect model efficiency because the efficiency of the electric power system focuses on meeting load demand. The coefficient of variation is calculated using Eq (31) [50].


$$Coefficient\;of\;variation=\left(\frac{Standard\;deviation}{mean}\right)*100$$ (31)

The standard deviation is calculated as

$$Standard\;deviation=\sqrt{\frac{1}{n}\sum_{i=1}^{n}{\left(x_{i}-mean\right)}^{2}}$$ (32)

where *x*_*i*_, is the value of gross reserve in a specific run *i* and *n* is the number of runs. In this study, the number of runs is ten.

Statistical test is also used to show if there is any significant difference between the old and new solutions of model III and model IV. An average % of improvement > 0.75 shows the improvement is significant [4, 51].

## VII. Results and discussions

Three (3) enhancements have been performed in stages on the single objective GMS model of [4] which has resulted in the proposed tri-objective GMS model. The enhancements are on the operational hours’ calculation, maintenance outage unit constraint, and objective function. Comparison has been performed to show the effect of enhancements on the cost, reliability (gross reserve), and violation. Table 6 displays the stages of enhancement where models II-IV contain enhancement from stages 1 to 3 respectively. Model I represents the single objective GMS model in [4] while model IV represents the proposed tri-objective GMS model.

**Table 6:** GMS model enhancement stages.

GMS model	Operational hours	Maintenance outage units’ constraint	Objective
I	Operating hours	Hard constraint	Single objective
II	Operating hours + start-up times	Hard constraint	Single objective
III	Operating hours + start-up times	Soft constraint	Single objective
IV	Operating hours + start-up times	Soft constraint	Tri-objective

The first enhancement is in model II where the calculation of operational hours is determined based on operating hours and start-up times rather than only operating hours, as in model I. The second enhancement is in model III where the maintenance outage unit’s constraint is considered as a soft constraint. Finally, the single objective is changed to a tri-objective in model IV.

Table 7 presents the results of the single objective GMS model I in [4] where the operational hours are determined based on operating hours only and the maintenance outage unit constraint is considered as a hard constraint. In this model, the single objective function is the operation cost. As observed from the results of model I, when the maintenance window gets bigger, cost and reliability are enhanced, where cost decreases and gross reserve increases. The increase in gross reserve also indicates that reliability increases. Moreover, there is no violation because the number of units specified for maintenance outage has been satisfied. The infeasible case occurred in the small maintenance window because more units reach the required operational hours for maintenance at the same time. In technical terms, in model I, the maintenance outage unit’s constraint is considered as a hard constraint. Thus, if the number of units to enter maintenance outage exceed the specified allowed number, the constraint is considered violated. Therefore, the ants will die and, thus, no solution will be obtained. Results of this model are stable, as indicated by the small values of the CV of the gross reserve.

**Table 7:** Results of GMS model I [4].

Test system	Maintenance window (hours)	Average of Cost ($)	Average of Gross Reserve (megawatt)	CV of Gross Reserve	Average of Violation
26-unit system	[1000–2000]	Infeasible	Infeasible	Infeasible	Infeasible
	[1000–2500]	192,681,251.60	1,927,988.40	0.28	-
	[1500–2500]	187,554,008.27	1,927,293.90	0.35	-
	[2000–3000]	179,644,201.80	1,938,113.40	0.58	-
	[2000–4000]	177,153,243.45	1,939,061.00	0.36	-
	[3000–5000]	174,032,798.93	1,940,223.20	0.48	-
32-unit system	[1000–2000]	Infeasible	Infeasible	Infeasible	Infeasible
	[1000–3000]	186,523,736.05	1,434,626.30	0.38	-
	[2000–3000]	181,272,798.33	1,436,243.40	0.44	-
	[2000–4000]	176,244,161.00	1,448,186.80	0.33	-
	[3000–4000]	175,567,883.1	1,447,940.20	0.17	-
	[3000–5000]	170,739,222.94	1,463,395.90	0.31	-
36-unit system	[1500–2500]	Infeasible	Infeasible	Infeasible	Infeasible
	[1500–3000]	357,401,622.58	2,378,498.80	0.25	-
	[2000–3000]	352,037,048.40	2,386,961.57	0.20	-
	[2000–4000]	345,018,935.31	2,388,690.40	0.24	-
	[3000–4000]	341,197,147.11	2,398,876.00	0.13	-
	[3000–5000]	336,385,061.33	2,412,457.50	0.15	-

Table 8 presents the results of the proposed GMS model II which includes the first modification to the single GMS model I, in which operational hours are determined based on operating hours and start-up times rather than only operating hours. On the practical side, considering start-up times in the calculation of operational hours can increase the generating unit’s life. Unit maintenance outage constraint is again considered as a hard constraint. In addition, operation cost is an objective function. In general, as the maintenance window gets bigger, the operation cost decreases and gross reserve increases. Moreover, there is no violation because the number of units specified for maintenance outage has been satisfied. The infeasible cases occurred in all the small maintenance windows (i.e., 1000 hours). In addition, there are infeasible solutions for a maintenance window of 1500 hours in the 36-unit system. The infeasible solution is because the load demand was not able to be fulfilled which led to the reserve constraint not being fulfilled because many units have to be maintained. In terms of model stability, results of this model are stable as indicated by the small values of the gross reserve CV.

**Table 8:** Results of GMS model II.

Test system	Maintenance window (hours)	Average of Cost ($)	Average of Gross Reserve (megawatt)	CV of Gross Reserve	Average of Violation
26-unit system	[1000–2000]	Infeasible	Infeasible	Infeasible	Infeasible
	[1000–2500]	196,720,168.33	1,924,943.80	0.47	-
	[1500–2500]	192,454,212.80	1,926,101.90	0.36	-
	[2000–3000]	185,474,225.21	1,932,042.80	0.32	-
	[2000–4000]	181,337,117.69	1,936,179.10	0.41	-
	[3000–5000]	177,476,155.76	1,939,274.10	0.32	-
32-unit system	[1000–2000]	Infeasible	Infeasible	Infeasible	Infeasible
	[1000–3000]	199,660,713.49	1,426,806.50	0.37	-
	[2000–3000]	190,004,272.74	1,430,944.40	0.23	-
	[2000–4000]	184,524,062.37	1,441,606.50	0.29	-
	[3000–4000]	180,313,013.39	1,442,544.30	0.23	-
	[3000–5000]	180,512,745.63	1,450,525.40	0.44	-
36-unit system	[1500–2500]	Infeasible	Infeasible	Infeasible	Infeasible
	[1500–3000]	Infeasible	Infeasible	Infeasible	Infeasible
	[2000–3000]	Infeasible	Infeasible	Infeasible	Infeasible
	[2000–4000]	351,516,032.43	2,386,753.90	0.29	-
	[3000–4000]	Infeasible	Infeasible	Infeasible	Infeasible
	[3000–5000]	341,413,584.20	2,398,587.00	0.39	-

Table 9 presents the results of the proposed GMS model III with two modifications, i.e., calculation of operational hours as in model II and maintenance outage unit constraint, considered as a soft constraint to consider the abnormal situations of not fulfilling load demand. Here, it can be observed, there is violation because the number of units’ maintenance outage is allowed to be more than the specific number. The cost and violation decrease while the gross reserve increases as the maintenance window gets bigger. Solutions were able to be obtained for the small maintenance windows with 26 and 32-unit systems because the maintenance outage unit constraint is considered as a soft constraint. Violation of the specified number of units for maintenance outage is allowed, and this led to feasible solutions being obtained. It can also be seen there are many infeasible solutions for the small maintenance window size (i.e., 1000 hours) for the 36-unit system because of the higher load demand which cannot satisfy the system reserve constraint. However, when the maintenance window gets bigger (i.e., more than 1000 hours), a feasible solution is achieved. This is possible because the maintenance outage unit’s constraint is considered as a soft constraint. Results were also stable as reflected by the small values of the CV for gross reserve.

**Table 9:** Results of GMS model III.

Test system	Maintenance window (hours)	Average of Cost ($)	Average of Gross Reserve (megawatt)	CV of Gross Reserve	Average of Violation
26-unit system	[1000–2000]	197,964,030.79	1,906,116.50	0.35	15.00
	[1000–2500]	185,838,070.13	1,924,890.90	0.42	2.40
	[1500–2500]	185,839,748.57	1,924,356.40	0.47	2.50
	[2000–3000]	182,660,051.31	1,927,152.00	0.39	2.50
	[2000–4000]	176,198,076.36	1,942,819.30	0.49	0.70
	[3000–5000]	172,657,223.42	1,941,358.10	0.53	0.80
32-unit system	[1000–2000]	202,079,493.83	1,415,519.00	0.35	26
	[1000–3000]	185,828,913.56	1,425,528.80	0.12	4.10
	[2000–3000]	185,589,183.77	1,428,827.40	0.29	2.90
	[2000–4000]	177,940,432.69	1,436,039.70	0.42	0.80
	[3000–4000]	178,574,832.69	1,440,257.00	0.48	0.70
	[3000–5000]	173,832,933.29	1,475,943.10	0.16	0.30
36-unit system	[1500–2500]	Infeasible	Infeasible	Infeasible	Infeasible
	[1500–3000]	355,602,679.90	2,374,858.00	0.21	12.00
	[2000–3000]	Infeasible	Infeasible	Infeasible	Infeasible
	[2000–4000]	351,023,284.61	2,387,366.00	0.18	0
	[3000–4000]	Infeasible	Infeasible	Infeasible	Infeasible
	[3000–5000]	341,378,311.12	2,399,930.75	0.21	0

Table 10 presents the results of the proposed tri-objective GMS model IV which includes all the enhancements, i.e., calculation of operational hours as in model II, and consideration of maintenance outage unit’s constraint as a soft constraint as in model III. In addition, there are now three (3) objective functions which are related to operation cost, gross reserve, and violation. The cost and violation decrease while the gross reserve increases as the maintenance window gets bigger. The feasible solutions are also being obtained with the 26 and 32-unit systems. The infeasible case occurred only in the small maintenance window (i.e., 1500–2500) of the 36-unit system. In this experiment, feasible solutions were obtained for the 36-unit system because the number of objectives was increased from one (1) to three (3). In the tri-objective model, load demand is satisfied because the gross reserve is considered as an additional objective function. Thus, a more reliable margin of generating capacity above the expected demand is created. In contrast, in the single objective model, where cost is the objective function and system reserve is considered as the constraint to satisfy load demand, which leads to the load demand may or may not be satisfied. The effect of the three (3) enhancements can be seen where model IV can satisfy the requirements of a real electrical power system. The requirements of a real power system cannot be satisfied by a single objective function. The results of the tri-objective model are also stable as reflected by the small CV values of the gross reserve.

**Table 10:** Results of GMS model IV.

Test system	Maintenance window (hours)	Average of Cost ($)	Average of Gross Reserve (megawatt)	CV of Gross Reserve	Average of Violation
26-unit system	[1000–2000]	197,192,377.15	1,969,874.40	0.05	8
	[1000–2500]	185,635,474.02	1,979,879.90	0.17	0
	[1500–2500]	185,528,532.40	1,973,633.40	0.16	0
	[2000–3000]	182,185,798.51	1,976,973.11	0.21	0
	[2000–4000]	175,838,519.23	2,000,809.60	0.27	0
	[3000–5000]	172,362,251.11	2,004,528.50	0.22	0
32-unit system	[1000–2000]	202,245,289.01	1,460,591.00	0.22	11.8
	[1000–3000]	185,665,522.90	1,465,829.50	0.25	0
	[2000–3000]	185,796,165.76	1,470,793.20	0.44	0
	[2000–4000]	178,177,512.72	1,481,697.70	0.09	0
	[3000–4000]	178,656,267.62	1,482,332.30	0.10	0
	[3000–5000]	173,949,330.13	1,491,474.60	0.03	0
36-unit system	[1500–2500]	Infeasible	Infeasible	Infeasible	Infeasible
	[1500–3000]	356,344,881.51	2,395,492.10	0.06	0
	[2000–3000]	359,986,683.12	2,397,650.30	0.14	0
	[2000–4000]	342,254,333.57	2,411,833.50	0.09	0
	[3000–4000]	343,022,850.70	2,408,575.00	0.13	0
	[3000–5000]	336,755,986.70	2,407,803.20	0.26	0

Tables 11–13 present the percentages of improvement on the three (3) objectives for the experimental results mentioned in Tables 7–10, which tested different GMS model scenarios. The formula to calculate the percentage of cost improvement (*PCI*) is based on [4], as presented in Eq (28). The formulae to calculate the percentage of violation improvement (*PVI*) and percentage of reliability improvement (*PRI*) are proposed in this study, as presented in Eqs (29) and (30) respectively. In Tables 11–13, the positive values denote a better result and negative values refer to no improvement. The term “infeasible” refers to the algorithm not being able to reach any solution in either model while the term “fail” refers to the algorithm not reaching any solution in the improved model. The term “success” refers to the algorithm managing to obtain solutions in the improved model.

**Table 11:** Percentage of cost, and gross reserve improvements between GMS model II and the basic GMS model I.

Test system	Maintenance window (hours)	*PCI* (%)	*PRI* (%)	*PVI* (%)
26-unit system	[1000–2000]	Infeasible	Infeasible	Infeasible
	[1000–2500]	-2.10	-0.16	-
	[1500–2500]	-2.61	-0.06	-
	[2000–3000]	-3.25	-0.31	-
	[2000–4000]	-2.36	-0.15	-
	[3000–5000]	-1.98	-0.05	-
32-unit system	[1000–2000]	Infeasible	Infeasible	Infeasible
	[1000–3000]	-7.04	-0.55	-
	[2000–3000]	-4.82	-0.37	-
	[2000–4000]	-4.70	-0.45	-
	[3000–4000]	-2.70	-0.37	-
	[3000–5000]	-5.72	-0.88	-
36-unit system	[1500–2500]	Infeasible	Infeasible	Infeasible
	[1500–3000]	Fail	Fail	-
	[2000–3000]	Fail	Fail	-
	[2000–4000]	-1.88	-0.08	-
	[3000–4000]	Fail	Fail	-
	[3000–5000]	-1.49	-0.57	-

**Table 12:** Percentage of cost, gross reserve, and violation improvements between GMS model III and GMS model II.

Test system	Maintenance window (hours)	Comparison between GMS model III and II		
		*PCI (%)*	*PRI (%)*	*PVI (%)*
26-unit system	[1000–2000]	**Success**	**Success**	**Success**
	[1000–2500]	**5.53**	-0.003	0
	[1500–2500]	**3.44**	-0.09	0
	[2000–3000]	**1.52**	-0.25	0
	[2000–4000]	**2.83**	**0.34**	0
	[3000–5000]	**2.72**	**0.11**	0
32-unit system	[1000–2000]	**Success**	**Success**	**Success**
	[1000–3000]	**6.93**	-0.09	0
	[2000–3000]	**2.32**	-0.15	0
	[2000–4000]	**3.57**	-0.39	0
	[3000–4000]	**0.96**	-0.16	0
	[3000–5000]	**3.70**	**1.75**	0
36-unit system	[1500–2500]	Infeasible	Infeasible	Infeasible
	[1500–3000]	**Success**	**Success**	**Success**
	[2000–3000]	Infeasible	Infeasible	Infeasible
	[2000–4000]	**0.14**	**0.03**	0
	[3000–4000]	Infeasible	Infeasible	Infeasible
	[3000–5000]	**0.01**	**0.06**	0

**Table 13:** Percentage of cost, gross reserve, and violation improvements between GMS model IV and GMS model III.

Test systems	Maintenance window (hours)	Comparison between GMS model IV and III		
		*PCI* (%)	*PRI* (%)	*PVI* (%)
26-unit system	[1000–2000]	**0.39**	**3.34**	**46.67**
	[1000–2500]	**0.11**	**2.86**	**100.00**
	[1500–2500]	**0.17**	**2.56**	**100.00**
	[2000–3000]	**0.26**	**2.59**	**100.00**
	[2000–4000]	**0.20**	**2.98**	**100.00**
	[3000–5000]	**0.17**	**3.25**	**100.00**
Average % improvement		0.22	**2.93**	**91.11**
32-unit system	[1000–2000]	-0.08	**3.18**	**54.62**
	[1000–3000]	**0.09**	**2.83**	**100.00**
	[2000–3000]	-0.11	**2.94**	**100.00**
	[2000–4000]	-0.13	**3.18**	**100.00**
	[3000–4000]	-0.05	**2.92**	**100.00**
	[3000–5000]	-0.07	**1.05**	**100.00**
Average % improvement		-0.06	**2.68**	**92.44**
36-unit system	[1500–2500]	Infeasible	Infeasible	Infeasible
	[1500–3000]	-0.21	**0.87**	**100**
	[2000–3000]	**Success**	**Success**	**Success**
	[2000–4000]	**2.50**	**1.02**	=
	[3000–4000]	**Success**	**Success**	**Success**
	[3000–5000]	**1.35**	**0.33**	=

Table 11 presents the percentage of improvement in cost, and gross reserve between GMS model II and GMS model I. There is no improvement in cost and gross reserve in GMS model II because the use of start-up times together with operating hours, in calculating the operational hours, has led to more units entering the maintenance outage which has resulted in the increased cost and decreased gross reserve. Table 12 presents the percentage of improvement in cost, gross reserve, and violation between GMS model III and GMS model II. There is improvement in cost for the 26- and 32-unit systems across all maintenance windows. However, the gross reserve and the violation were not improved. In general, improvement in gross reserve was only recorded for larger maintenance windows because the load demand is satisfied as a result of an increase in the search space. The violation did not record any improvement because the maintenance outage unit’s constraint is considered as a hard constraint in model II; thus, the violation is not allowed for this constraint. However, the violation is allowed in model III because the maintenance unit outage is considered as a soft constraint. Feasible solutions were obtained (denoted success) in several maintenance windows of model III where, previously, the algorithm was not able to obtain any solution in model II. This is because violation of the maintenance outage constraint is permitted which increases the algorithm’s search space. There are many infeasible solutions for the 36-unit system, especially for small maintenance window size because of the higher load demand which cannot satisfy the system reserve constraint.

Table 13 presents the percentage of improvement in cost, gross reserve, and violation between tri-objective GMS model IV (i.e., the proposed tri-objective GMS model) and the single GMS model III. There are improvements in cost, gross reserve, and violation for the 26-unit system across all sizes of maintenance window. There are also improvements in gross reserve and violation for the 32-unit system but not in cost. However, the very small differences in cost were recorded across most of the maintenance window sizes. From the algorithm perspective, the global update in the single model (i.e., model III) focuses only on the cost objective function solution whereas in model IV the global update depends on the compromise solutions of the three (3) objective functions. The trade-off between the solutions of the objective functions will affect the cost. This scenario does not occur in the 26-unit system because its complexity is less than the 32-unit system.

There are also mixed results for the 36-unit system. Infeasible solutions for both models, i.e., III and IV were obtained in the small maintenance window size (i.e., 1000 hours) and the early stage of operation. This caused more units to enter the maintenance outage, thus the demand is not satisfied. For the second maintenance window, there are improvements in gross reserve and violation but no improvement in cost. However, the difference between models for cost is small. Model IV was able to obtain solutions in small maintenance window size (i.e., 1000 hours) for cost, gross reserve, and violation (indicated by “success”) as opposed to model III which failed to obtain any solution. This is due to the system gross reserve being considered as an objective in model IV which helps to create a more reliable margin of generated capacity above the expected demand led to satisfy the system reserve constraint. However, this objective is not considered in model III. Thus, the higher load demand was not satisfied which cannot satisfy the system reserve constraint. There are improvements in cost and gross reserve for bigger window sizes but there is no violation for both models. This is indicated by “=“ in Table 13.

The statistical test shows that there is significant improvement in reliability and violation metrics for the 26- and 32- unit maintenance windows. However, there is no improvement for cost metric in the 32-unit system. However, the improvement in cost metric with the 26-unit system is not significant. Statistical test was not possible for the 36-unit system because there are mixed results.

**Fig 3:**
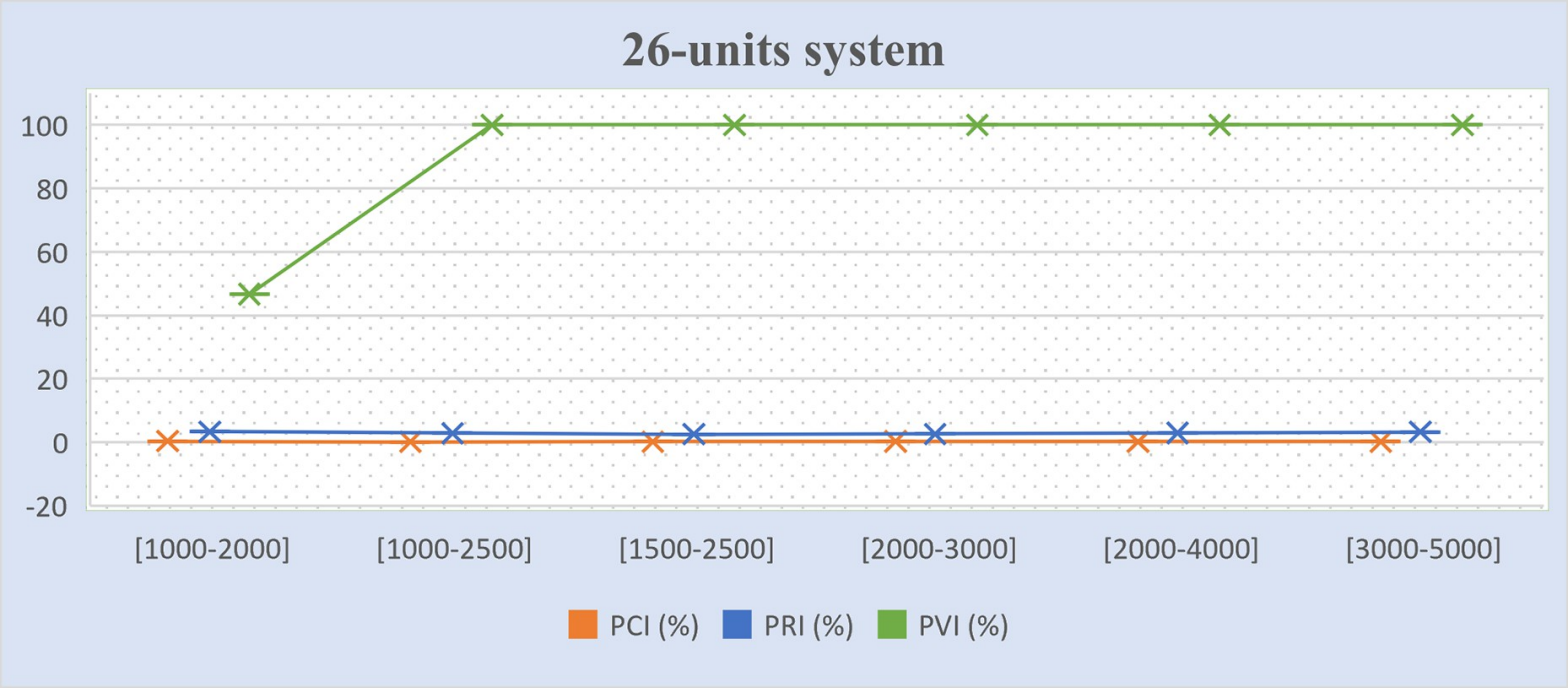
Percentage of improvement in cost, reliability, & violation for GMS model IV with 26-units system.

**Fig 4:**
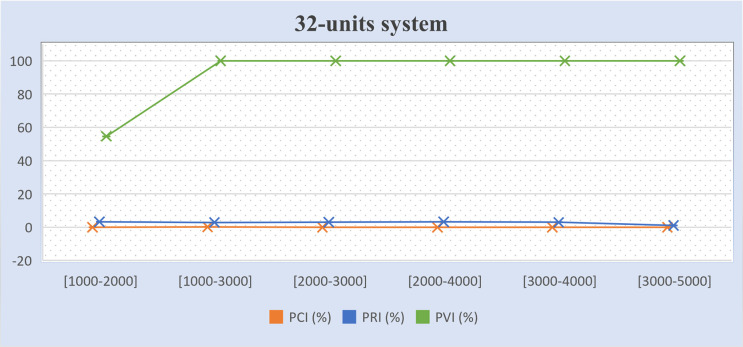
Percentage of improvement in cost, reliability, & violation for GMS model IV with 32-units system.

**Fig 5:**
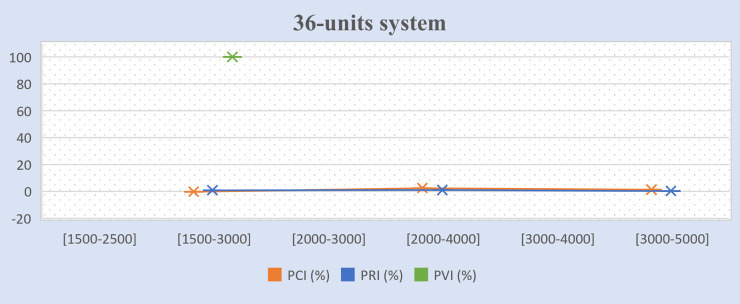
Percentage of improvement in cost, reliability, & violation for GMS model IV with 36-units system.

Results in Table 13 on percentage of improvement in cost, gross reserve, and violation between the tri-objective GMS model IV and single GMS model III are again, displayed in Figs 3–5. In particular, there is a bigger improvement in the violation objective as compared to the cost and reliability objectives in all the three (3) test systems. This is because the violation is an objective function in this tri-objective model whereas, in the single-objective model, the objective function is the cost. There is also an improvement in reliability as this is also an objective function in the tri-objective model whereas it is considered as a constraint in the single objective model. There is no improvement in cost as it is considered as an objective in both models. However, improvement in cost is achieved with the 36-unit system in the bigger maintenance windows.

## VIII. Conclusion

This paper has proposed a tri-objective GMS model, GMS graph model, and a PACS algorithm for the maintenance scheduling of generating units. The proposed tri-objective GMS model is developed to consider multi-criteria in electrical power systems as the objective functions, which include cost, reliability (gross reserve), and convenience (violation). Comparison between a single GMS model and proposed GMS model has shown that the tri-objective GMS model has many advantages that reflect the requirement of a real-world power system. This study is an important step toward scheduling generating units by considering the tri-objective GMS model with sequential strategy based on an operating hours and start-up times. In addition, the proposed GMS graph model, and PACS algorithm is developed to consider the proposed model. The proposed graph model can be used for scheduling the maintenance of generating units and developing the PACS algorithm. The proposed algorithm can be used to implement the proposed model and optimizes a maintenance scheduling solution in terms of reducing operational costs, increasing gross reserve, and reducing violations. The case study from IEEE RTS systems with 26, 32, 36 generating units was used to investigate the performance of the proposed model. Computational results demonstrated that the performance evaluation of the proposed tri-objective GMS model is superior to the benchmark model in different initial operational hours of units in producing the schedule for the GMS in terms of the two objective functions’ metrics (i.e., reliability and violation). In addition, the probability for providing more feasible solutions in most of the maintenance windows is increased in the proposed tri-objective GMS model.

Future work related to GMS can include risk or stochastic reliability measures in GMS model formulation. Work can also consider solving the transmission maintenance scheduling problem in combination with the GMS problem besides involving other constraints such as resource constraints to identify a limit on the available amount of resources for the aim of maintenance. These resources can include service budgets and the availability of spare parts, as well as the availability of manpower for maintenance work. Future work can also consider the transmission/network constraints that must be met in order to ensure the transmission capabilities of the electrical network (e.g., maintaining voltage levels).

## Supporting information

S1 Dataset (PDF)
