# Supplementary material for: Tri-objective generator maintenance scheduling model based on sequential strategy
Source: PLoS One. 2022 Oct 18;17(10):e0276225. doi: 10.1371/journal.pone.0276225 (PMC9578641; doi:10.1371/journal.pone.0276225)
Supplement: S1 Dataset — (PDF) [file pone.0276225.s001.pdf]

Dataset for the 26-unit test system

Units	C_i^{Fx}	C_i^P	C_i^M	P_i^{min}	P_i^{max}	oph_i^s	Up time	Down time	$\text{Ini}_{\text{hourS}}$
1	24.389	25.547	255470	2.4	12	1000	1	1	-1
2	24.411	25.675	256750	2.4	12	800	1	1	-1
3	24.638	25.803	258030	2.4	12	1200	1	1	-1
4	24.761	25.932	259320	2.4	12	1300	1	1	-1
5	24.888	26.061	260610	2.4	12	2100	1	1	-1
6	117.755	37.551	375510	4	20	100	1	1	-1
7	118.108	37.664	376640	4	20	1900	1	1	-1
8	118.458	37.777	377770	4	20	1900	1	1	-1
9	118.821	37.89	378900	4	20	800	1	1	-1
10	81.136	13.327	133270	15.2	76	540	3	2	3
11	81.298	13.354	133540	15.2	76	800	3	2	3
12	81.464	13.381	133810	15.2	76	3100	3	2	3
13	81.626	13.407	134070	15.2	76	2300	3	2	3
14	217.895	18	180000	25	500	200	4	2	-3
15	218.335	18.1	181000	25	100	600	4	2	-3
16	218.775	18.2	182000	25	100	1800	4	7	-3
17	142.735	10.694	106940	54.25	155	900	5	3	5
18	143.029	10.715	107150	54.25	155	1200	5	3	5
19	143.318	10.737	107370	54.25	155	300	5	3	5
20	143.597	10.758	107580	54.25	155	500	5	3	5
21	259.131	23	230000	68.95	197	2000	5	10	-4
22	259.649	23.1	231000	68.95	197	3450	5	4	-4
23	260.176	23.2	232000	68.95	197	1000	5	4	-4
24	177.058	10.862	108620	140	350	700	8	5	10
25	310.002	7.492	74920	100	400	500	8	5	10
26	311.91	7.503	75030	100	400	1450	8	5	10

Dataset for the 32-unit system

Units	C_i^{Fx}	C_i^{P}	C_i^{M}	P_i^{min}	P_i^{max}	oph_i^s	Up Time	Down Time	Ini_{hourS}
1	24.389	25.547	255470	2.4	12	1000	1	1	-1
2	24.411	25.675	256750	2.4	12	800	1	1	-1
3	24.638	25.803	258030	2.4	12	1200	1	1	-1
4	24.761	25.932	259320	2.4	12	1300	1	1	-1
5	24.888	26.061	260610	2.4	12	2100	1	1	-1
6	118.908	37.964	379640	4	20	100	1	1	-1
7	118.458	37.777	377770	4	20	1900	1	1	-1
8	118.908	37.964	379640	4	20	1900	1	1	-1
9	119.458	38.777	387770	4	20	800	1	1	-1
10	81.826	13.507	135070	15.2	76	540	3	2	3
11	81.136	13.327	133270	15.2	76	800	3	2	3
12	81.298	13.354	133540	15.2	76	3100	3	2	3
13	81.626	13.407	134070	15.2	76	2300	3	2	3
14	217.895	18	180000	25	100	200	4	2	5
15	219.775	18.6	186000	25	100	1000	4	2	5
16	218.335	18.1	181000	25	100	600	4	2	5
17	216.775	18.3	183000	25	100	2200	4	2	-3
18	218.775	18.2	182000	25	100	1800	4	2	-3
19	216.775	17.3	173000	25	100	1400	4	2	-3
20	142.735	10.737	107370	54.25	155	900	4	2	-3
21	143.029	10.715	107150	54.25	155	1200	5	3	5
22	143.318	10.737	107370	54.25	155	300	5	3	5
23	143.597	10.758	107580	54.25	155	500	5	3	5
24	259.131	23	230000	68.95	197	2000	5	4	-4
25	259.649	23.1	231000	68.95	197	3450	5	4	-4
26	260.176	23.2	232000	68.95	197	1000	5	4	-4
27	260.576	23.4	234000	68.95	197	100	5	4	-4
28	261.176	23.5	235000	68.95	197	540	5	4	-4
29	260.076	23.04	230400	68.95	197	900	5	4	-4
30	176.057	10.842	108420	140	350	700	8	5	10
31	310.002	7.492	74920	100	400	500	8	5	10
32	311.91	7.503	75030	100	400	1450	8	5	10

Dataset for the 36-unit system

Units	C_i^{Fx}	C_i^P	C_i^M	P_i^{min}	P_i^{max}	oph_i^s	Up Time	Down Time	Ini_{hourS}
1	24.38	25.5	24380	2.4	12	1000	1	1	-1
2	118.9	37.9	118900	4	20	800	1	1	-1
3	118.45	37.7	118450	4	20	1200	1	1	-1
4	118.9	37.9	118900	4	20	1300	1	1	-1
5	119.45	38.7	119450	4	20	2100	1	1	-1
6	117.75	37.5	117750	4	20	100	1	1	-1
7	118.1	37.66	118100	4	20	1900	1	1	-1
8	81.8	13.5	81800	15.2	76	1900	3	2	-1
9	81.13	13.32	81130	15.2	76	800	3	2	-1
10	81.29	13.35	81290	15.2	76	540	3	2	3
11	81.62	13.4	81620	15.2	76	800	3	2	3
12	217.89	18	217890	25	100	3100	4	2	3
13	219.77	18.6	219770	25	100	2300	4	2	3
14	218.33	18.1	218330	25	100	200	4	2	-3
15	216.77	18.2	216770	25	100	600	4	2	-3
16	218.77	18.2	218770	25	100	1800	4	2	-3
17	216.775	17.2	216775	25	100	900	4	2	5
18	218.775	19.2	218775	25	100	1200	4	2	5
19	143.02	10.71	143020	54.25	155	300	5	3	5
20	143.31	10.73	143310	54.25	155	500	5	3	5
21	143.59	10.75	143590	54.25	155	2000	5	3	-4
22	259.13	23	259130	68.95	197	3450	5	4	-4
23	259.64	23.1	259640	68.95	197	1000	5	4	-4
24	260.17	23.2	260170	68.95	197	700	5	4	10
25	260.57	23.4	260570	68.95	197	500	5	4	10
26	261.17	23.5	261170	68.95	197	1450	5	4	10
27	260.07	23.04	260070	68.95	197	1900	5	4	10
28	176.05	10.84	176050	140	350	800	8	5	10
29	177.05	10.86	177050	140	350	300	8	5	10
30	176.05	10.66	176050	140	350	500	8	5	10
31	177.95	10.96	177950	140	350	2000	8	5	10
32	310	7.49	310000	100	400	3450	8	5	10
33	311.9	7.5	311900	100	400	200	8	5	10
34	312.9	7.51	312900	100	400	600	8	5	10
35	314.9	7.53	314900	100	400	1800	8	5	10
36	313.9	7.61	313900	100	400	900	8	5	10

Dataset for the Load Demand with 26-unit system and 32-unit system

Periods	Hours	Weeks						
		Sunday (weekday)	Monday (weekday)	Tuesday (weekday)	Wednesday (weekday)	Thursday (weekday)	Friday (weekend)	Saturday (weekend)
Period (1)	12-1 am	1236	1533	1648	1648	1582	1804	1477
Period (1)	1-2 am	1162	1441	1550	1550	1488	1665	1364
Period (1)	2-3 am	1107	1373	1476	1476	1417	1572	1288
Period (1)	3-4 am	1089	1350	1451	1451	1393	1526	1250
Period (1)	4-5 am	1089	1350	1451	1451	1393	1480	1212
Period (1)	5-6 am	1107	1373	1476	1476	1417	1503	1231
Period (1)	6-7 am	1365	1693	1820	1820	1748	1526	1250
Period (1)	7-8 am	1587	1968	2116	2116	2031	1619	1326
Period (1)	8-9 am	1753	2173	2337	2337	2244	1850	1515
Period (1)	9-10 am	1771	2196	2362	2362	2267	2035	1667
Period (1)	10-11am	1771	2196	2362	2362	2267	2081	1705
Period (1)	11am-12 pm	1753	2173	2337	2337	2244	2104	1724
Period (1)	12-1 pm	1753	2173	2337	2337	2244	2081	1705
Period (1)	1-2 pm	1753	2173	2337	2337	2244	2035	1667
Period (1)	2-3 pm	1716	2128	2288	2288	2196	2012	1648
Period (1)	3-4 pm	1734	2151	2312	2312	2220	2012	1648
Period (1)	4-5 pm	1827	2265	2435	2435	2338	2104	1724
Period (1)	5-6 pm	1845	2288	2460	2460	2362	2312	1894
Period (1)	6-7 pm	1845	2288	2460	2460	2362	2289	1875
Period (1)	7-8 pm	1771	2196	2362	2362	2267	2243	1837
Period (1)	8-9 pm	1679	2082	2239	2239	2149	2174	1781
Period (1)	9-10 pm	1531	1899	2042	2042	1960	2127	1743
Period (1)	10-11 pm	1347	1670	1796	1796	1724	2012	1648
Period (1)	11pm-12am	1162	1441	1550	1550	1488	1873	1534
Period (2)	12-1 am	1291	1601	1722	1722	1653	1884	1544
Period (2)	1-2 am	1214	1506	1619	1619	1554	1739	1425
Period (2)	2-3 am	1157	1434	1542	1542	1480	1643	1346
Period (2)	3-4 am	1137	1410	1516	1516	1456	1594	1306
Period (2)	4-5 am	1137	1410	1516	1516	1456	1546	1266

Period (2)	5-6 am	1157	1434	1542	1542	1480	1570	1286
Period (2)	6-7 am	1426	1769	1902	1902	1826	1594	1306
Period (2)	7-8 am	1658	2055	2210	2210	2122	1691	1385
Period (2)	8-9 am	1831	2271	2442	2442	2344	1933	1583
Period (2)	9-10 am	1850	2294	2467	2467	2369	2126	1741
Period (2)	10-11am	1850	2294	2467	2467	2369	2174	1781
Period (2)	11am-12 pm	1831	2271	2442	2442	2344	2198	1801
Period (2)	12-1 pm	1831	2271	2442	2442	2344	2174	1781
Period (2)	1-2 pm	1831	2271	2442	2442	2344	2126	1741
Period (2)	2-3 pm	1793	2223	2390	2390	2294	2102	1722
Period (2)	3-4 pm	1812	2247	2416	2416	2319	2102	1722
Period (2)	4-5 pm	1908	2366	2544	2544	2443	2198	1801
Period (2)	5-6 pm	1928	2390	2570	2570	2467	2416	1979
Period (2)	6-7 pm	1928	2390	2570	2570	2467	2392	1959
Period (2)	7-8 pm	1850	2294	2467	2467	2369	2343	1920
Period (2)	8-9 pm	1754	2175	2339	2339	2245	2271	1860
Period (2)	9-10 pm	1600	1984	2133	2133	2048	2223	1821
Period (2)	10-11 pm	1407	1745	1876	1876	1801	2102	1722
Period (2)	11pm-12am	1214	1506	1619	1619	1554	1957	1603
Period (3)	12-1 am	1256	1558	1675	1675	1608	1833	1502
Period (3)	1-2 am	1181	1465	1575	1575	1512	1692	1386
Period (3)	2-3 am	1125	1395	1500	1500	1440	1598	1309
Period (3)	3-4 am	1106	1372	1475	1475	1416	1551	1271
Period (3)	4-5 am	1106	1372	1475	1475	1416	1504	1232
Period (3)	5-6 am	1125	1395	1500	1500	1440	1528	1251
Period (3)	6-7 am	1388	1721	1850	1850	1776	1551	1271
Period (3)	7-8 am	1613	2000	2150	2150	2064	1645	1348
Period (3)	8-9 am	1781	2209	2375	2375	2280	1880	1540
Period (3)	9-10 am	1800	2232	2400	2400	2304	2068	1694
Period (3)	10-11am	1800	2232	2400	2400	2304	2115	1733
Period (3)	11am-12 pm	1781	2209	2375	2375	2280	2139	1752
Period (3)	12-1 pm	1781	2209	2375	2375	2280	2115	1733
Period (3)	1-2 pm	1781	2209	2375	2375	2280	2068	1694
Period (3)	2-3 pm	1744	2162	2325	2325	2232	2045	1675
Period (3)	3-4 pm	1763	2186	2350	2350	2256	2045	1675
Period (3)	4-5 pm	1856	2302	2475	2475	2376	2139	1752
Period	5-6 pm	1875	2325	2500	2500	2400	2350	1925

(3)								
Period (3)	6-7 pm	1875	2325	2500	2500	2400	2327	1906
Period (3)	7-8 pm	1800	2232	2400	2400	2304	2280	1867
Period (3)	8-9 pm	1706	2116	2275	2275	2184	2209	1810
Period (3)	9-10 pm	1556	1930	2075	2075	1992	2162	1771
Period (3)	10-11 pm	1369	1697	1825	1825	1752	2045	1675
Period (4)	11pm-12am	1181	1465	1575	1575	1512	1904	1559
Period (4)	12-1 am	1196	1483	1595	1595	1531	1745	1429
Period (4)	1-2 am	1125	1394	1499	1499	1439	1611	1319
Period (4)	2-3 am	1071	1328	1428	1428	1371	1521	1246
Period (4)	3-4 am	1053	1306	1404	1404	1348	1477	1210
Period (4)	4-5 am	1053	1306	1404	1404	1348	1432	1173
Period (4)	5-6 am	1071	1328	1428	1428	1371	1454	1191
Period (4)	6-7 am	1321	1638	1761	1761	1691	1477	1210
Period (4)	7-8 am	1535	1904	2047	2047	1965	1566	1283
Period (4)	8-9 am	1696	2103	2261	2261	2171	1790	1466
Period (4)	9-10 am	1714	2125	2285	2285	2193	1969	1613
Period (4)	10-11am	1714	2125	2285	2285	2193	2013	1649
Period (4)	11am-12 pm	1696	2103	2261	2261	2171	2036	1668
Period (4)	12-1 pm	1696	2103	2261	2261	2171	2013	1649
Period (4)	1-2 pm	1696	2103	2261	2261	2171	1969	1613
Period (4)	2-3 pm	1660	2058	2213	2213	2125	1946	1594
Period (4)	3-4 pm	1678	2081	2237	2237	2148	1946	1594
Period (4)	4-5 pm	1767	2191	2356	2356	2262	2036	1668
Period (4)	5-6 pm	1785	2213	2380	2380	2285	2237	1833
Period (4)	6-7 pm	1785	2213	2380	2380	2285	2215	1814
Period (4)	7-8 pm	1714	2125	2285	2285	2193	2170	1778
Period (4)	8-9 pm	1624	2014	2166	2166	2079	2103	1723
Period (4)	9-10 pm	1482	1837	1975	1975	1896	2058	1686
Period (4)	10-11 pm	1303	1616	1737	1737	1668	1946	1594
Period (4)	11pm-12am	1125	1394	1499	1499	1439	1812	1484
Period (5)	12-1 am	1261	1564	1682	1682	1614	1840	1508
Period (5)	1-2 am	1186	1471	1581	1581	1518	1699	1392
Period (5)	2-3 am	1130	1401	1506	1506	1446	1604	1314
Period (5)	3-4 am	1111	1377	1481	1481	1422	1557	1276
Period (5)	4-5 am	1111	1377	1481	1481	1422	1510	1237
Period (5)	5-6 am	1130	1401	1506	1506	1446	1534	1256

Period (5)	6-7 am	1393	1727	1857	1857	1783	1557	1276
Period (5)	7-8 am	1619	2007	2159	2159	2072	1652	1353
Period (5)	8-9 am	1788	2218	2385	2385	2289	1888	1546
Period (5)	9-10 am	1807	2241	2410	2410	2313	2076	1701
Period (5)	10-11am	1807	2241	2410	2410	2313	2123	1739
Period (5)	11am-12 pm	1788	2218	2385	2385	2289	2147	1759
Period (5)	12-1 pm	1788	2218	2385	2385	2289	2123	1739
Period (5)	1-2 pm	1788	2218	2385	2385	2289	2076	1701
Period (5)	2-3 pm	1751	2171	2334	2334	2241	2053	1681
Period (5)	3-4 pm	1770	2194	2359	2359	2265	2053	1681
Period (5)	4-5 pm	1864	2311	2485	2485	2386	2147	1759
Period (5)	5-6 pm	1883	2334	2510	2510	2410	2359	1933
Period (5)	6-7 pm	1883	2334	2510	2510	2410	2336	1913
Period (5)	7-8 pm	1807	2241	2410	2410	2313	2289	1875
Period (5)	8-9 pm	1713	2124	2284	2284	2193	2218	1817
Period (5)	9-10 pm	1562	1937	2083	2083	2000	2171	1778
Period (5)	10-11 pm	1374	1704	1832	1832	1759	2053	1681
Period (5)	11pm-12am	1186	1471	1581	1581	1518	1911	1565
Period (6)	12-1 am	1206	1495	1608	1608	1544	1760	1441
Period (6)	1-2 am	1134	1406	1512	1512	1452	1624	1331
Period (6)	2-3 am	1080	1339	1440	1440	1382	1534	1257
Period (6)	3-4 am	1062	1317	1416	1416	1359	1489	1220
Period (6)	4-5 am	1062	1317	1416	1416	1359	1444	1183
Period (6)	5-6 am	1080	1339	1440	1440	1382	1466	1201
Period (6)	6-7 am	1332	1652	1776	1776	1705	1489	1220
Period (6)	7-8 am	1548	1920	2064	2064	1981	1579	1294
Period (6)	8-9 am	1710	2120	2280	2280	2189	1805	1478
Period (6)	9-10 am	1728	2143	2304	2304	2212	1985	1626
Period (6)	10-11am	1728	2143	2304	2304	2212	2030	1663
Period (6)	11am-12 pm	1710	2120	2280	2280	2189	2053	1682
Period (6)	12-1 pm	1710	2120	2280	2280	2189	2030	1663
Period (6)	1-2 pm	1710	2120	2280	2280	2189	1985	1626
Period (6)	2-3 pm	1674	2076	2232	2232	2143	1963	1608
Period (6)	3-4 pm	1692	2098	2256	2256	2166	1963	1608
Period (6)	4-5 pm	1782	2210	2376	2376	2281	2053	1682
Period (6)	5-6 pm	1800	2232	2400	2400	2304	2256	1848
Period	6-7 pm	1800	2232	2400	2400	2304	2233	1830

(6)								
Period (6)	7-8 pm	1728	2143	2304	2304	2212	2188	1793
Period (6)	8-9 pm	1638	2031	2184	2184	2097	2121	1737
Period (6)	9-10 pm	1494	1853	1992	1992	1912	2076	1700
Period (6)	10-11 pm	1314	1629	1752	1752	1682	1963	1608
Period (6)	11pm-12am	1134	1406	1512	1512	1452	1827	1497
Period (7)	12-1 am	1191	1477	1588	1588	1524	1738	1423
Period (7)	1-2 am	1120	1389	1493	1493	1433	1604	1314
Period (7)	2-3 am	1067	1322	1422	1422	1365	1515	1241
Period (7)	3-4 am	1049	1300	1398	1398	1342	1470	1204
Period (7)	4-5 am	1049	1300	1398	1398	1342	1426	1168
Period (7)	5-6 am	1067	1322	1422	1422	1365	1448	1186
Period (7)	6-7 am	1315	1631	1754	1754	1684	1470	1204
Period (7)	7-8 am	1529	1896	2038	2038	1957	1559	1277
Period (7)	8-9 am	1689	2094	2252	2252	2161	1782	1460
Period (7)	9-10 am	1706	2116	2275	2275	2184	1960	1606
Period (7)	10-11am	1706	2116	2275	2275	2184	2005	1642
Period (7)	11am-12 pm	1689	2094	2252	2252	2161	2027	1661
Period (7)	12-1 pm	1689	2094	2252	2252	2161	2005	1642
Period (7)	1-2 pm	1689	2094	2252	2252	2161	1960	1606
Period (7)	2-3 pm	1653	2050	2204	2204	2116	1938	1588
Period (7)	3-4 pm	1671	2072	2228	2228	2139	1938	1588
Period (7)	4-5 pm	1760	2182	2346	2346	2252	2027	1661
Period (7)	5-6 pm	1778	2204	2370	2370	2275	2228	1825
Period (7)	6-7 pm	1778	2204	2370	2370	2275	2206	1807
Period (7)	7-8 pm	1706	2116	2275	2275	2184	2161	1770
Period (7)	8-9 pm	1618	2006	2157	2157	2070	2094	1715
Period (7)	9-10 pm	1475	1829	1967	1967	1888	2050	1679
Period (7)	10-11 pm	1298	1609	1730	1730	1661	1938	1588
Period (7)	11pm-12am	1120	1389	1493	1493	1433	1805	1478
Period (8)	12-1 am	1156	1433	1541	1541	1479	1686	1381
Period (8)	1-2 am	1087	1348	1449	1449	1391	1557	1275
Period (8)	2-3 am	1035	1283	1380	1380	1325	1470	1204
Period (8)	3-4 am	1018	1262	1357	1357	1303	1427	1169
Period (8)	4-5 am	1018	1262	1357	1357	1303	1384	1133
Period (8)	5-6 am	1035	1283	1380	1380	1325	1405	1151
Period (8)	6-7 am	1277	1583	1702	1702	1634	1427	1169

Period (8)	7-8 am	1484	1840	1978	1978	1899	1513	1240
Period (8)	8-9 am	1639	2032	2185	2185	2098	1730	1417
Period (8)	9-10 am	1656	2053	2208	2208	2120	1903	1558
Period (8)	10-11am	1656	2053	2208	2208	2120	1946	1594
Period (8)	11am-12 pm	1639	2032	2185	2185	2098	1967	1612
Period (8)	12-1 pm	1639	2032	2185	2185	2098	1946	1594
Period (8)	1-2 pm	1639	2032	2185	2185	2098	1903	1558
Period (8)	2-3 pm	1604	1989	2139	2139	2053	1881	1541
Period (8)	3-4 pm	1622	2011	2162	2162	2076	1881	1541
Period (8)	4-5 pm	1708	2118	2277	2277	2186	1967	1612
Period (8)	5-6 pm	1725	2139	2300	2300	2208	2162	1771
Period (8)	6-7 pm	1725	2139	2300	2300	2208	2140	1753
Period (8)	7-8 pm	1656	2053	2208	2208	2120	2097	1718
Period (8)	8-9 pm	1570	1946	2093	2093	2009	2032	1665
Period (8)	9-10 pm	1432	1775	1909	1909	1833	1989	1629
Period (8)	10-11 pm	1259	1561	1679	1679	1612	1881	1541
Period (8)	11pm-12am	1087	1348	1449	1449	1391	1751	1435
Period (9)	12-1 am	997	1236	1329	1329	1276	1488	1219
Period (9)	1-2 am	981	1216	1308	1308	1256	1448	1186
Period (9)	2-3 am	950	1177	1266	1266	1215	1369	1121
Period (9)	3-4 am	918	1138	1224	1224	1175	1309	1072
Period (9)	4-5 am	934	1158	1245	1245	1195	1289	1056
Period (9)	5-6 am	1029	1275	1372	1372	1317	1289	1056
Period (9)	6-7 am	1139	1413	1519	1519	1458	1349	1105
Period (9)	7-8 am	1345	1668	1794	1794	1722	1468	1202
Period (9)	8-9 am	1503	1864	2005	2005	1924	1646	1349
Period (9)	9-10 am	1567	1942	2089	2089	2005	1765	1446
Period (9)	10-11am	1583	1962	2110	2110	2026	1825	1495
Period (9)	11am-12 pm	1567	1942	2089	2089	2005	1864	1527
Period (9)	12-1 pm	1472	1825	1962	1962	1884	1805	1478
Period (9)	1-2 pm	1456	1805	1941	1941	1864	1785	1462
Period (9)	2-3 pm	1424	1766	1899	1899	1823	1785	1462
Period (9)	3-4 pm	1393	1727	1857	1857	1783	1706	1397
Period (9)	4-5 pm	1424	1766	1899	1899	1823	1686	1381
Period (9)	5-6 pm	1456	1805	1941	1941	1864	1745	1430
Period (9)	6-7 pm	1519	1884	2026	2026	1945	1825	1495
Period	7-8 pm	1551	1923	2068	2068	1985	1983	1625

(9)								
Period (9)	8-9 pm	1519	1884	2026	2026	1945	1924	1576
Period (9)	9-10 pm	1424	1766	1899	1899	1823	1884	1543
Period (9)	10-11 pm	1266	1570	1688	1688	1620	1785	1462
Period (9)	11pm-12am	1108	1373	1477	1477	1418	1686	1381
Period (10)	12-1 am	992	1230	1323	1323	1270	1481	1213
Period (10)	1-2 am	977	1211	1302	1302	1250	1441	1180
Period (10)	2-3 am	945	1172	1260	1260	1210	1362	1116
Period (10)	3-4 am	914	1133	1218	1218	1169	1303	1067
Period (10)	4-5 am	929	1152	1239	1239	1189	1283	1051
Period (10)	5-6 am	1024	1269	1365	1365	1310	1283	1051
Period (10)	6-7 am	1134	1406	1512	1512	1452	1342	1100
Period (10)	7-8 am	1339	1660	1785	1785	1714	1461	1197
Period (10)	8-9 am	1496	1855	1995	1995	1915	1638	1342
Period (10)	9-10 am	1559	1933	2079	2079	1996	1757	1439
Period (10)	10-11am	1575	1953	2100	2100	2016	1816	1488
Period (10)	11am-12 pm	1559	1933	2079	2079	1996	1856	1520
Period (10)	12-1 pm	1465	1816	1953	1953	1875	1796	1471
Period (10)	1-2 pm	1449	1797	1932	1932	1855	1777	1455
Period (10)	2-3 pm	1418	1758	1890	1890	1814	1777	1455
Period (10)	3-4 pm	1386	1719	1848	1848	1774	1698	1391
Period (10)	4-5 pm	1418	1758	1890	1890	1814	1678	1374
Period (10)	5-6 pm	1449	1797	1932	1932	1855	1737	1423
Period (10)	6-7 pm	1512	1875	2016	2016	1935	1816	1488
Period (10)	7-8 pm	1544	1914	2058	2058	1976	1974	1617
Period (10)	8-9 pm	1512	1875	2016	2016	1935	1915	1568
Period (10)	9-10 pm	1418	1758	1890	1890	1814	1875	1536
Period (10)	10-11 pm	1260	1562	1680	1680	1613	1777	1455
Period (10)	11pm-12am	1103	1367	1470	1470	1411	1678	1374
Period (11)	12-1 am	964	1195	1285	1285	1234	1438	1178
Period (11)	1-2 am	949	1176	1265	1265	1214	1400	1147
Period (11)	2-3 am	918	1138	1224	1224	1175	1323	1084
Period (11)	3-4 am	887	1100	1183	1183	1136	1266	1037
Period (11)	4-5 am	903	1119	1204	1204	1155	1246	1021
Period (11)	5-6 am	995	1233	1326	1326	1273	1246	1021
Period (11)	6-7 am	1102	1366	1469	1469	1410	1304	1068
Period (11)	7-8 am	1301	1612	1734	1734	1665	1419	1162

Period (11)	8-9 am	1454	1802	1938	1938	1860	1592	1304
Period (11)	9-10 am	1515	1878	2020	2020	1939	1707	1398
Period (11)	10-11am	1530	1897	2040	2040	1958	1764	1445
Period (11)	11am-12 pm	1515	1878	2020	2020	1939	1803	1477
Period (11)	12-1 pm	1423	1764	1897	1897	1821	1745	1429
Period (11)	1-2 pm	1408	1745	1877	1877	1802	1726	1414
Period (11)	2-3 pm	1377	1707	1836	1836	1763	1726	1414
Period (11)	3-4 pm	1346	1669	1795	1795	1723	1649	1351
Period (11)	4-5 pm	1377	1707	1836	1836	1763	1630	1335
Period (11)	5-6 pm	1408	1745	1877	1877	1802	1687	1382
Period (11)	6-7 pm	1469	1821	1958	1958	1880	1764	1445
Period (11)	7-8 pm	1499	1859	1999	1999	1919	1918	1571
Period (11)	8-9 pm	1469	1821	1958	1958	1880	1860	1524
Period (11)	9-10 pm	1377	1707	1836	1836	1763	1822	1492
Period (11)	10-11 pm	1224	1518	1632	1632	1567	1726	1414
Period (11)	11pm-12am	1071	1328	1428	1428	1371	1630	1335
Period (12)	12-1 am	978	1213	1304	1304	1252	1459	1195
Period (12)	1-2 am	963	1194	1283	1283	1232	1420	1164
Period (12)	2-3 am	932	1155	1242	1242	1192	1343	1100
Period (12)	3-4 am	900	1117	1201	1201	1153	1284	1052
Period (12)	4-5 am	916	1136	1221	1221	1172	1265	1036
Period (12)	5-6 am	1009	1251	1346	1346	1292	1265	1036
Period (12)	6-7 am	1118	1386	1490	1490	1431	1323	1084
Period (12)	7-8 am	1320	1636	1760	1760	1689	1440	1179
Period (12)	8-9 am	1475	1829	1967	1967	1888	1615	1323
Period (12)	9-10 am	1537	1906	2049	2049	1967	1732	1419
Period (12)	10-11am	1553	1925	2070	2070	1987	1790	1466
Period (12)	11am-12 pm	1537	1906	2049	2049	1967	1829	1498
Period (12)	12-1 pm	1444	1790	1925	1925	1848	1771	1450
Period (12)	1-2 pm	1428	1771	1904	1904	1828	1751	1435
Period (12)	2-3 pm	1397	1733	1863	1863	1788	1751	1435
Period (12)	3-4 pm	1366	1694	1822	1822	1749	1673	1371
Period (12)	4-5 pm	1397	1733	1863	1863	1788	1654	1355
Period (12)	5-6 pm	1428	1771	1904	1904	1828	1712	1403
Period (12)	6-7 pm	1490	1848	1987	1987	1908	1790	1466
Period (12)	7-8 pm	1521	1887	2029	2029	1947	1946	1594
Period	8-9 pm	1490	1848	1987	1987	1908	1887	1546

(12)								
Period (12)	9-10 pm	1397	1733	1863	1863	1788	1849	1514
Period (12)	10-11 pm	1242	1540	1656	1656	1590	1751	1435
Period (12)	11pm-12am	1087	1348	1449	1449	1391	1654	1355
Period (13)	12-1 am	950	764	1266	1266	1216	1417	1161
Period (13)	1-2 am	935	740	1246	1246	1196	1379	1130
Period (13)	2-3 am	905	693	1206	1206	1158	1304	1068
Period (13)	3-4 am	874	648	1166	1166	1119	1247	1021
Period (13)	4-5 am	889	670	1186	1186	1138	1228	1006
Period (13)	5-6 am	980	813	1307	1307	1254	1228	1006
Period (13)	6-7 am	1085	998	1447	1447	1389	1285	1052
Period (13)	7-8 am	1281	1391	1709	1709	1640	1398	1145
Period (13)	8-9 am	1432	1737	1910	1910	1833	1568	1285
Period (13)	9-10 am	1492	1887	1990	1990	1910	1682	1377
Period (13)	10-11am	1508	1925	2010	2010	1930	1738	1424
Period (13)	11am-12 pm	1492	1887	1990	1990	1910	1776	1455
Period (13)	12-1 pm	1402	1665	1869	1869	1795	1719	1408
Period (13)	1-2 pm	1387	1629	1849	1849	1775	1700	1393
Period (13)	2-3 pm	1357	1559	1809	1809	1737	1700	1393
Period (13)	3-4 pm	1327	1491	1769	1769	1698	1625	1331
Period (13)	4-5 pm	1357	1559	1809	1809	1737	1606	1316
Period (13)	5-6 pm	1387	1629	1849	1849	1775	1663	1362
Period (13)	6-7 pm	1447	1774	1930	1930	1852	1738	1424
Period (13)	7-8 pm	1477	1849	1970	1970	1891	1889	1548
Period (13)	8-9 pm	1447	1774	1930	1930	1852	1833	1501
Period (13)	9-10 pm	1357	1559	1809	1809	1737	1795	1470
Period (13)	10-11 pm	1206	1232	1608	1608	1544	1700	1393
Period (13)	11pm-12am	1055	943	1407	1407	1351	1606	1316
Period (14)	12-1 am	1011	1254	1348	1348	1294	1509	1236
Period (14)	1-2 am	995	1234	1327	1327	1274	1468	1203
Period (14)	2-3 am	963	1194	1284	1284	1233	1388	1137
Period (14)	3-4 am	931	1154	1241	1241	1192	1328	1088
Period (14)	4-5 am	947	1174	1263	1263	1212	1308	1071
Period (14)	5-6 am	1043	1294	1391	1391	1335	1308	1071
Period (14)	6-7 am	1156	1433	1541	1541	1479	1368	1121
Period (14)	7-8 am	1364	1692	1819	1819	1746	1489	1219
Period (14)	8-9 am	1525	1891	2033	2033	1952	1670	1368

Period (14)	9-10 am	1589	1970	2119	2119	2034	1790	1467
Period (14)	10-11am	1605	1990	2140	2140	2054	1851	1516
Period (14)	11am-12 pm	1589	1970	2119	2119	2034	1891	1549
Period (14)	12-1 pm	1493	1851	1990	1990	1911	1831	1499
Period (14)	1-2 pm	1477	1831	1969	1969	1890	1810	1483
Period (14)	2-3 pm	1445	1791	1926	1926	1849	1810	1483
Period (14)	3-4 pm	1412	1751	1883	1883	1808	1730	1417
Period (14)	4-5 pm	1445	1791	1926	1926	1849	1710	1401
Period (14)	5-6 pm	1477	1831	1969	1969	1890	1770	1450
Period (14)	6-7 pm	1541	1910	2054	2054	1972	1851	1516
Period (14)	7-8 pm	1573	1950	2097	2097	2013	2012	1648
Period (14)	8-9 pm	1541	1910	2054	2054	1972	1951	1598
Period (14)	9-10 pm	1445	1791	1926	1926	1849	1911	1565
Period (14)	10-11 pm	1284	1592	1712	1712	1644	1810	1483
Period (14)	11pm-12am	1124	1393	1498	1498	1438	1710	1401
Period (15)	12-1 am	969	1201	1292	1292	1240	1445	1184
Period (15)	1-2 am	953	1182	1271	1271	1220	1407	1152
Period (15)	2-3 am	923	1144	1230	1230	1181	1330	1089
Period (15)	3-4 am	892	1106	1189	1189	1141	1272	1042
Period (15)	4-5 am	907	1125	1210	1210	1161	1253	1026
Period (15)	5-6 am	999	1240	1333	1333	1279	1253	1026
Period (15)	6-7 am	1107	1373	1476	1476	1417	1310	1073
Period (15)	7-8 am	1307	1621	1743	1743	1673	1426	1168
Period (15)	8-9 am	1461	1812	1948	1948	1870	1599	1310
Period (15)	9-10 am	1522	1888	2030	2030	1948	1715	1405
Period (15)	10-11am	1538	1907	2050	2050	1968	1773	1452
Period (15)	11am-12 pm	1522	1888	2030	2030	1948	1811	1484
Period (15)	12-1 pm	1430	1774	1907	1907	1830	1754	1436
Period (15)	1-2 pm	1415	1754	1886	1886	1811	1734	1421
Period (15)	2-3 pm	1384	1716	1845	1845	1771	1734	1421
Period (15)	3-4 pm	1353	1678	1804	1804	1732	1657	1358
Period (15)	4-5 pm	1384	1716	1845	1845	1771	1638	1342
Period (15)	5-6 pm	1415	1754	1886	1886	1811	1696	1389
Period (15)	6-7 pm	1476	1831	1968	1968	1889	1773	1452
Period (15)	7-8 pm	1507	1869	2009	2009	1929	1927	1579
Period (15)	8-9 pm	1476	1831	1968	1968	1889	1869	1531
Period	9-10 pm	1384	1716	1845	1845	1771	1831	1500

(15)								
Period (15)	10-11 pm	1230	1526	1640	1640	1574	1734	1421
Period (15)	11pm-12am	1076	1335	1435	1435	1378	1638	1342
Period (16)	12-1 am	1077	1336	1436	1436	1379	1607	1317
Period (16)	1-2 am	1060	1314	1414	1414	1357	1565	1282
Period (16)	2-3 am	1026	1272	1368	1368	1313	1479	1211
Period (16)	3-4 am	992	1230	1322	1322	1270	1415	1159
Period (16)	4-5 am	1009	1251	1345	1345	1291	1393	1141
Period (16)	5-6 am	1112	1378	1482	1482	1423	1393	1141
Period (16)	6-7 am	1231	1526	1642	1642	1576	1457	1194
Period (16)	7-8 am	1454	1802	1938	1938	1860	1586	1299
Period (16)	8-9 am	1625	2014	2166	2166	2079	1779	1457
Period (16)	9-10 am	1693	2099	2257	2257	2167	1907	1562
Period (16)	10-11am	1710	2120	2280	2280	2189	1972	1615
Period (16)	11am-12 pm	1693	2099	2257	2257	2167	2015	1650
Period (16)	12-1 pm	1590	1972	2120	2120	2036	1950	1598
Period (16)	1-2 pm	1573	1950	2098	2098	2014	1929	1580
Period (16)	2-3 pm	1539	1908	2052	2052	1970	1929	1580
Period (16)	3-4 pm	1505	1866	2006	2006	1926	1843	1510
Period (16)	4-5 pm	1539	1908	2052	2052	1970	1822	1492
Period (16)	5-6 pm	1573	1950	2098	2098	2014	1886	1545
Period (16)	6-7 pm	1642	2035	2189	2189	2101	1972	1615
Period (16)	7-8 pm	1676	2078	2234	2234	2145	2143	1756
Period (16)	8-9 pm	1642	2035	2189	2189	2101	2079	1703
Period (16)	9-10 pm	1539	1908	2052	2052	1970	2036	1668
Period (16)	10-11 pm	1368	1696	1824	1824	1751	1929	1580
Period (16)	11pm-12am	1197	1484	1596	1596	1532	1822	1492
Period (17)	12-1 am	1016	1260	1355	1355	1300	1516	1242
Period (17)	1-2 am	1000	1240	1333	1333	1280	1475	1209
Period (17)	2-3 am	968	1200	1290	1290	1238	1394	1142
Period (17)	3-4 am	935	1160	1247	1247	1197	1334	1093
Period (17)	4-5 am	951	1180	1269	1269	1218	1314	1076
Period (17)	5-6 am	1048	1300	1398	1398	1342	1314	1076
Period (17)	6-7 am	1161	1440	1548	1548	1486	1374	1126
Period (17)	7-8 am	1371	1700	1828	1828	1754	1496	1225
Period (17)	8-9 am	1532	1900	2043	2043	1961	1677	1374
Period (17)	9-10 am	1596	1980	2129	2129	2043	1799	1473

Period (17)	10-11am	1613	2000	2150	2150	2064	1859	1523
Period (17)	11am-12 pm	1596	1980	2129	2129	2043	1900	1556
Period (17)	12-1 pm	1500	1860	2000	2000	1920	1839	1507
Period (17)	1-2 pm	1484	1840	1978	1978	1899	1819	1490
Period (17)	2-3 pm	1451	1800	1935	1935	1858	1819	1490
Period (17)	3-4 pm	1419	1760	1892	1892	1816	1738	1424
Period (17)	4-5 pm	1451	1800	1935	1935	1858	1718	1407
Period (17)	5-6 pm	1484	1840	1978	1978	1899	1778	1457
Period (17)	6-7 pm	1548	1920	2064	2064	1981	1859	1523
Period (17)	7-8 pm	1580	1960	2107	2107	2023	2021	1656
Period (17)	8-9 pm	1548	1920	2064	2064	1981	1960	1606
Period (17)	9-10 pm	1451	1800	1935	1935	1858	1920	1573
Period (17)	10-11 pm	1290	1600	1720	1720	1651	1819	1490
Period (17)	11pm-12am	1129	1400	1505	1505	1445	1718	1407
Period (18)	12-1 am	1147	1423	1530	1530	1468	1662	1362
Period (18)	1-2 am	1076	1334	1434	1434	1377	1573	1288
Period (18)	2-3 am	1040	1289	1386	1386	1331	1483	1215
Period (18)	3-4 am	1004	1245	1338	1338	1285	1460	1196
Period (18)	4-5 am	1004	1245	1338	1338	1285	1438	1178
Period (18)	5-6 am	1040	1289	1386	1386	1331	1393	1141
Period (18)	6-7 am	1147	1423	1530	1530	1468	1393	1141
Period (18)	7-8 am	1362	1689	1816	1816	1744	1483	1215
Period (18)	8-9 am	1559	1934	2079	2079	1996	1820	1491
Period (18)	9-10 am	1703	2112	2271	2271	2180	1932	1583
Period (18)	10-11am	1775	2200	2366	2366	2271	2044	1675
Period (18)	11am-12 pm	1793	2223	2390	2390	2294	2089	1711
Period (18)	12-1 pm	1775	2200	2366	2366	2271	2089	1711
Period (18)	1-2 pm	1793	2223	2390	2390	2294	2067	1693
Period (18)	2-3 pm	1793	2223	2390	2390	2294	2044	1675
Period (18)	3-4 pm	1739	2156	2318	2318	2226	2044	1675
Period (18)	4-5 pm	1721	2134	2294	2294	2203	2067	1693
Period (18)	5-6 pm	1721	2134	2294	2294	2203	2112	1730
Period (18)	6-7 pm	1667	2067	2223	2223	2134	2134	1748
Period (18)	7-8 pm	1649	2045	2199	2199	2111	2134	1748
Period (18)	8-9 pm	1649	2045	2199	2199	2111	2247	1840
Period (18)	9-10 pm	1667	2067	2223	2223	2134	2089	1711
Period	10-11 pm	1559	1934	2079	2079	1996	1977	1619

(18)								
Period (18)	11pm-12am	1291	1600	1721	1721	1652	1797	1472
Period (19)	12-1 am	1190	1476	1587	1587	1524	1725	1413
Period (19)	1-2 am	1116	1384	1488	1488	1428	1632	1337
Period (19)	2-3 am	1079	1338	1438	1438	1381	1539	1260
Period (19)	3-4 am	1042	1292	1389	1389	1333	1515	1241
Period (19)	4-5 am	1042	1292	1389	1389	1333	1492	1222
Period (19)	5-6 am	1079	1338	1438	1438	1381	1445	1184
Period (19)	6-7 am	1190	1476	1587	1587	1524	1445	1184
Period (19)	7-8 am	1414	1753	1885	1885	1809	1539	1260
Period (19)	8-9 am	1618	2007	2158	2158	2071	1888	1547
Period (19)	9-10 am	1767	2191	2356	2356	2262	2005	1642
Period (19)	10-11am	1841	2283	2455	2455	2357	2121	1738
Period (19)	11am-12 pm	1860	2306	2480	2480	2381	2168	1776
Period (19)	12-1 pm	1841	2283	2455	2455	2357	2168	1776
Period (19)	1-2 pm	1860	2306	2480	2480	2381	2145	1757
Period (19)	2-3 pm	1860	2306	2480	2480	2381	2121	1738
Period (19)	3-4 pm	1804	2237	2406	2406	2309	2121	1738
Period (19)	4-5 pm	1786	2214	2381	2381	2286	2145	1757
Period (19)	5-6 pm	1786	2214	2381	2381	2286	2191	1795
Period (19)	6-7 pm	1730	2145	2306	2306	2214	2215	1814
Period (19)	7-8 pm	1711	2122	2282	2282	2190	2215	1814
Period (19)	8-9 pm	1711	2122	2282	2282	2190	2331	1910
Period (19)	9-10 pm	1730	2145	2306	2306	2214	2168	1776
Period (19)	10-11 pm	1618	2007	2158	2158	2071	2051	1680
Period (19)	11pm-12am	1339	1661	1786	1786	1714	1865	1528
Period (20)	12-1 am	1205	1494	1606	1606	1542	1746	1430
Period (20)	1-2 am	1130	1401	1506	1506	1446	1652	1353
Period (20)	2-3 am	1092	1354	1456	1456	1398	1557	1276
Period (20)	3-4 am	1054	1307	1406	1406	1349	1534	1256
Period (20)	4-5 am	1054	1307	1406	1406	1349	1510	1237
Period (20)	5-6 am	1092	1354	1456	1456	1398	1463	1198
Period (20)	6-7 am	1205	1494	1606	1606	1542	1463	1198
Period (20)	7-8 am	1431	1774	1908	1908	1831	1557	1276
Period (20)	8-9 am	1638	2031	2184	2184	2096	1911	1565
Period (20)	9-10 am	1788	2218	2385	2385	2289	2029	1662
Period (20)	10-11am	1864	2311	2485	2485	2386	2147	1759

Period (20)	11am-12 pm	1883	2334	2510	2510	2410	2194	1797
Period (20)	12-1 pm	1864	2311	2485	2485	2386	2194	1797
Period (20)	1-2 pm	1883	2334	2510	2510	2410	2171	1778
Period (20)	2-3 pm	1883	2334	2510	2510	2410	2147	1759
Period (20)	3-4 pm	1826	2264	2435	2435	2337	2147	1759
Period (20)	4-5 pm	1807	2241	2410	2410	2313	2171	1778
Period (20)	5-6 pm	1807	2241	2410	2410	2313	2218	1817
Period (20)	6-7 pm	1751	2171	2334	2334	2241	2241	1836
Period (20)	7-8 pm	1732	2148	2309	2309	2217	2241	1836
Period (20)	8-9 pm	1732	2148	2309	2309	2217	2359	1933
Period (20)	9-10 pm	1751	2171	2334	2334	2241	2194	1797
Period (20)	10-11 pm	1638	2031	2184	2184	2096	2076	1701
Period (20)	11pm-12am	1355	1681	1807	1807	1735	1888	1546
Period (21)	12-1 am	1171	1452	1562	1562	1499	1697	1390
Period (21)	1-2 am	1098	1362	1464	1464	1405	1606	1315
Period (21)	2-3 am	1061	1316	1415	1415	1359	1514	1240
Period (21)	3-4 am	1025	1271	1366	1366	1312	1491	1221
Period (21)	4-5 am	1025	1271	1366	1366	1312	1468	1202
Period (21)	5-6 am	1061	1316	1415	1415	1359	1422	1165
Period (21)	6-7 am	1171	1452	1562	1562	1499	1422	1165
Period (21)	7-8 am	1391	1725	1854	1854	1780	1514	1240
Period (21)	8-9 am	1592	1974	2123	2123	2038	1858	1522
Period (21)	9-10 am	1739	2156	2318	2318	2225	1972	1616
Period (21)	10-11am	1812	2247	2416	2416	2319	2087	1710
Period (21)	11am-12 pm	1830	2269	2440	2440	2342	2133	1747
Period (21)	12-1 pm	1812	2247	2416	2416	2319	2133	1747
Period (21)	1-2 pm	1830	2269	2440	2440	2342	2110	1728
Period (21)	2-3 pm	1830	2269	2440	2440	2342	2087	1710
Period (21)	3-4 pm	1775	2201	2367	2367	2272	2087	1710
Period (21)	4-5 pm	1757	2178	2342	2342	2249	2110	1728
Period (21)	5-6 pm	1757	2178	2342	2342	2249	2156	1766
Period (21)	6-7 pm	1702	2110	2269	2269	2178	2179	1785
Period (21)	7-8 pm	1684	2088	2245	2245	2155	2179	1785
Period (21)	8-9 pm	1684	2088	2245	2245	2155	2294	1879
Period (21)	9-10 pm	1702	2110	2269	2269	2178	2133	1747
Period (21)	10-11 pm	1592	1974	2123	2123	2038	2018	1653
Period	11pm-	1318	1634	1757	1757	1687	1835	1503

(21)	12am							
Period (22)	12-1 am	1109	929	1478	1478	1419	1607	1316
Period (22)	1-2 am	1040	817	1386	1386	1331	1520	1245
Period (22)	2-3 am	1005	763	1340	1340	1286	1433	1174
Period (22)	3-4 am	970	712	1294	1294	1242	1411	1156
Period (22)	4-5 am	970	712	1294	1294	1242	1390	1138
Period (22)	5-6 am	1005	763	1340	1340	1286	1346	1103
Period (22)	6-7 am	1109	929	1478	1478	1419	1346	1103
Period (22)	7-8 am	1317	1311	1756	1756	1685	1433	1174
Period (22)	8-9 am	1507	1718	2010	2010	1929	1759	1441
Period (22)	9-10 am	1646	2048	2195	2195	2107	1867	1530
Period (22)	10-11am	1715	2224	2287	2287	2195	1976	1619
Period (22)	11am-12 pm	1733	2269	2310	2310	2218	2019	1654
Period (22)	12-1 pm	1715	2224	2287	2287	2195	2019	1654
Period (22)	1-2 pm	1733	2269	2310	2310	2218	1998	1636
Period (22)	2-3 pm	1733	2269	2310	2310	2218	1976	1619
Period (22)	3-4 pm	1681	2135	2241	2241	2151	1976	1619
Period (22)	4-5 pm	1663	2091	2218	2218	2129	1998	1636
Period (22)	5-6 pm	1663	2091	2218	2218	2129	2041	1672
Period (22)	6-7 pm	1611	1963	2148	2148	2062	2063	1690
Period (22)	7-8 pm	1594	1921	2125	2125	2040	2063	1690
Period (22)	8-9 pm	1594	1921	2125	2125	2040	2171	1779
Period (22)	9-10 pm	1611	1963	2148	2148	2062	2019	1654
Period (22)	10-11 pm	1507	1718	2010	2010	1929	1911	1565
Period (22)	11pm-12am	1247	1176	1663	1663	1597	1737	1423
Period (23)	12-1 am	1234	1530	1645	1645	1579	1788	1464
Period (23)	1-2 am	1157	1434	1542	1542	1480	1691	1385
Period (23)	2-3 am	1118	1386	1491	1491	1431	1594	1306
Period (23)	3-4 am	1079	1338	1439	1439	1382	1570	1286
Period (23)	4-5 am	1079	1338	1439	1439	1382	1546	1266
Period (23)	5-6 am	1118	1386	1491	1491	1431	1498	1227
Period (23)	6-7 am	1234	1530	1645	1645	1579	1498	1227
Period (23)	7-8 am	1465	1816	1953	1953	1875	1594	1306
Period (23)	8-9 am	1677	2079	2236	2236	2146	1957	1603
Period (23)	9-10 am	1831	2271	2442	2442	2344	2078	1702
Period (23)	10-11am	1908	2366	2544	2544	2443	2198	1801
Period (23)	11am-12 pm	1928	2390	2570	2570	2467	2247	1840

Period (23)	12-1 pm	1908	2366	2544	2544	2443	2247	1840
Period (23)	1-2 pm	1928	2390	2570	2570	2467	2223	1821
Period (23)	2-3 pm	1928	2390	2570	2570	2467	2198	1801
Period (23)	3-4 pm	1870	2318	2493	2493	2393	2198	1801
Period (23)	4-5 pm	1850	2294	2467	2467	2369	2223	1821
Period (23)	5-6 pm	1850	2294	2467	2467	2369	2271	1860
Period (23)	6-7 pm	1793	2223	2390	2390	2294	2295	1880
Period (23)	7-8 pm	1773	2199	2364	2364	2270	2295	1880
Period (23)	8-9 pm	1773	2199	2364	2364	2270	2416	1979
Period (23)	9-10 pm	1793	2223	2390	2390	2294	2247	1840
Period (23)	10-11 pm	1677	2079	2236	2236	2146	2126	1741
Period (23)	11pm-12am	1388	1721	1850	1850	1776	1933	1583
Period (24)	12-1 am	1214	1506	1619	1619	1554	1760	1442
Period (24)	1-2 am	1139	1412	1518	1518	1457	1665	1364
Period (24)	2-3 am	1101	1365	1467	1467	1409	1570	1286
Period (24)	3-4 am	1063	1318	1417	1417	1360	1546	1266
Period (24)	4-5 am	1063	1318	1417	1417	1360	1522	1247
Period (24)	5-6 am	1101	1365	1467	1467	1409	1474	1208
Period (24)	6-7 am	1214	1506	1619	1619	1554	1474	1208
Period (24)	7-8 am	1442	1788	1923	1923	1846	1570	1286
Period (24)	8-9 am	1651	2047	2201	2201	2113	1926	1578
Period (24)	9-10 am	1803	2235	2404	2404	2307	2045	1675
Period (24)	10-11am	1879	2329	2505	2505	2405	2164	1773
Period (24)	11am-12 pm	1898	2353	2530	2530	2429	2212	1812
Period (24)	12-1 pm	1879	2329	2505	2505	2405	2212	1812
Period (24)	1-2 pm	1898	2353	2530	2530	2429	2188	1792
Period (24)	2-3 pm	1898	2353	2530	2530	2429	2164	1773
Period (24)	3-4 pm	1841	2282	2454	2454	2356	2164	1773
Period (24)	4-5 pm	1822	2259	2429	2429	2332	2188	1792
Period (24)	5-6 pm	1822	2259	2429	2429	2332	2236	1831
Period (24)	6-7 pm	1765	2188	2353	2353	2259	2259	1851
Period (24)	7-8 pm	1746	2165	2328	2328	2234	2259	1851
Period (24)	8-9 pm	1746	2165	2328	2328	2234	2378	1948
Period (24)	9-10 pm	1765	2188	2353	2353	2259	2212	1812
Period (24)	10-11 pm	1651	2047	2201	2201	2113	2093	1714
Period (24)	11pm-12am	1366	1694	1822	1822	1749	1903	1558
Period	12-1 am	1224	1518	1632	1632	1567	1774	1453

(25)								
Period (25)	1-2 am	1148	1423	1530	1530	1469	1678	1374
Period (25)	2-3 am	1109	1375	1479	1479	1420	1582	1296
Period (25)	3-4 am	1071	1328	1428	1428	1371	1558	1276
Period (25)	4-5 am	1071	1328	1428	1428	1371	1534	1257
Period (25)	5-6 am	1109	1375	1479	1479	1420	1486	1217
Period (25)	6-7 am	1224	1518	1632	1632	1567	1486	1217
Period (25)	7-8 am	1454	1802	1938	1938	1860	1582	1296
Period (25)	8-9 am	1664	2063	2219	2219	2130	1942	1590
Period (25)	9-10 am	1817	2253	2423	2423	2326	2061	1689
Period (25)	10-11am	1893	2348	2525	2525	2424	2181	1787
Period (25)	11am-12 pm	1913	2372	2550	2550	2448	2229	1826
Period (25)	12-1 pm	1893	2348	2525	2525	2424	2229	1826
Period (25)	1-2 pm	1913	2372	2550	2550	2448	2205	1806
Period (25)	2-3 pm	1913	2372	2550	2550	2448	2181	1787
Period (25)	3-4 pm	1855	2300	2474	2474	2375	2181	1787
Period (25)	4-5 pm	1836	2277	2448	2448	2350	2205	1806
Period (25)	5-6 pm	1836	2277	2448	2448	2350	2253	1846
Period (25)	6-7 pm	1779	2205	2372	2372	2277	2277	1865
Period (25)	7-8 pm	1760	2182	2346	2346	2252	2277	1865
Period (25)	8-9 pm	1760	2182	2346	2346	2252	2397	1964
Period (25)	9-10 pm	1779	2205	2372	2372	2277	2229	1826
Period (25)	10-11 pm	1664	2063	2219	2219	2130	2109	1728
Period (25)	11pm-12am	1377	1707	1836	1836	1763	1918	1571
Period (26)	12-1 am	1176	1458	1568	1568	1505	1704	1396
Period (26)	1-2 am	1103	1367	1470	1470	1411	1612	1321
Period (26)	2-3 am	1066	1322	1421	1421	1364	1520	1245
Period (26)	3-4 am	1029	1276	1372	1372	1317	1497	1226
Period (26)	4-5 am	1029	1276	1372	1372	1317	1474	1207
Period (26)	5-6 am	1066	1322	1421	1421	1364	1428	1170
Period (26)	6-7 am	1176	1458	1568	1568	1505	1428	1170
Period (26)	7-8 am	1397	1732	1862	1862	1788	1520	1245
Period (26)	8-9 am	1599	1982	2132	2132	2046	1865	1528
Period (26)	9-10 am	1746	2165	2328	2328	2234	1981	1622
Period (26)	10-11am	1819	2256	2426	2426	2328	2096	1717
Period (26)	11am-12 pm	1838	2279	2450	2450	2352	2142	1754
Period (26)	12-1 pm	1819	2256	2426	2426	2328	2142	1754

Period (26)	1-2 pm	1838	2279	2450	2450	2352	2119	1736
Period (26)	2-3 pm	1838	2279	2450	2450	2352	2096	1717
Period (26)	3-4 pm	1782	2210	2377	2377	2281	2096	1717
Period (26)	4-5 pm	1764	2187	2352	2352	2258	2119	1736
Period (26)	5-6 pm	1764	2187	2352	2352	2258	2165	1773
Period (26)	6-7 pm	1709	2119	2279	2279	2187	2188	1792
Period (26)	7-8 pm	1691	2096	2254	2254	2164	2188	1792
Period (26)	8-9 pm	1691	2096	2254	2254	2164	2303	1887
Period (26)	9-10 pm	1709	2119	2279	2279	2187	2142	1754
Period (26)	10-11 pm	1599	1982	2132	2132	2046	2027	1660
Period (26)	11pm-12am	1323	1641	1764	1764	1693	1842	1509
Period (27)	12-1 am	1032	1280	1376	1376	1321	1496	1225
Period (27)	1-2 am	968	1200	1290	1290	1238	1415	1159
Period (27)	2-3 am	935	1160	1247	1247	1197	1334	1093
Period (27)	3-4 am	903	1120	1204	1204	1156	1314	1076
Period (27)	4-5 am	903	1120	1204	1204	1156	1293	1060
Period (27)	5-6 am	935	1160	1247	1247	1197	1253	1026
Period (27)	6-7 am	1032	1280	1376	1376	1321	1253	1026
Period (27)	7-8 am	1226	1520	1634	1634	1569	1334	1093
Period (27)	8-9 am	1403	1740	1871	1871	1796	1637	1341
Period (27)	9-10 am	1532	1900	2043	2043	1961	1738	1424
Period (27)	10-11am	1596	1980	2129	2129	2043	1839	1507
Period (27)	11am-12 pm	1613	2000	2150	2150	2064	1880	1540
Period (27)	12-1 pm	1596	1980	2129	2129	2043	1880	1540
Period (27)	1-2 pm	1613	2000	2150	2150	2064	1859	1523
Period (27)	2-3 pm	1613	2000	2150	2150	2064	1839	1507
Period (27)	3-4 pm	1564	1940	2086	2086	2002	1839	1507
Period (27)	4-5 pm	1548	1920	2064	2064	1981	1859	1523
Period (27)	5-6 pm	1548	1920	2064	2064	1981	1900	1556
Period (27)	6-7 pm	1500	1860	2000	2000	1920	1920	1573
Period (27)	7-8 pm	1484	1840	1978	1978	1899	1920	1573
Period (27)	8-9 pm	1484	1840	1978	1978	1899	2021	1656
Period (27)	9-10 pm	1500	1860	2000	2000	1920	1880	1540
Period (27)	10-11 pm	1403	1740	1871	1871	1796	1778	1457
Period (27)	11pm-12am	1161	1440	1548	1548	1486	1617	1324
Period (28)	12-1 am	1118	1387	1491	1491	1432	1621	1328
Period	1-2 am	1049	1300	1398	1398	1342	1533	1256

(28)								
Period (28)	2-3 am	1014	1257	1351	1351	1297	1446	1184
Period (28)	3-4 am	979	1213	1305	1305	1253	1424	1166
Period (28)	4-5 am	979	1213	1305	1305	1253	1402	1148
Period (28)	5-6 am	1014	1257	1351	1351	1297	1358	1112
Period (28)	6-7 am	1118	1387	1491	1491	1432	1358	1112
Period (28)	7-8 am	1328	1647	1771	1771	1700	1446	1184
Period (28)	8-9 am	1520	1885	2027	2027	1946	1774	1453
Period (28)	9-10 am	1660	2059	2214	2214	2125	1884	1543
Period (28)	10-11am	1730	2145	2307	2307	2214	1993	1633
Period (28)	11am-12 pm	1748	2167	2330	2330	2237	2037	1669
Period (28)	12-1 pm	1730	2145	2307	2307	2214	2037	1669
Period (28)	1-2 pm	1748	2167	2330	2330	2237	2015	1651
Period (28)	2-3 pm	1748	2167	2330	2330	2237	1993	1633
Period (28)	3-4 pm	1695	2102	2260	2260	2170	1993	1633
Period (28)	4-5 pm	1678	2080	2237	2237	2147	2015	1651
Period (28)	5-6 pm	1678	2080	2237	2237	2147	2059	1686
Period (28)	6-7 pm	1625	2015	2167	2167	2080	2081	1704
Period (28)	7-8 pm	1608	1994	2144	2144	2058	2081	1704
Period (28)	8-9 pm	1608	1994	2144	2144	2058	2190	1794
Period (28)	9-10 pm	1625	2015	2167	2167	2080	2037	1669
Period (28)	10-11 pm	1520	1885	2027	2027	1946	1927	1579
Period (28)	11pm-12am	1258	1560	1678	1678	1610	1752	1435
Period (29)	12-1 am	1094	1357	1459	1459	1401	1586	1299
Period (29)	1-2 am	1026	1272	1368	1368	1313	1500	1229
Period (29)	2-3 am	992	1230	1322	1322	1270	1415	1159
Period (29)	3-4 am	958	1187	1277	1277	1226	1393	1141
Period (29)	4-5 am	958	1187	1277	1277	1226	1372	1124
Period (29)	5-6 am	992	1230	1322	1322	1270	1329	1088
Period (29)	6-7 am	1094	1357	1459	1459	1401	1329	1088
Period (29)	7-8 am	1300	1612	1733	1733	1663	1415	1159
Period (29)	8-9 am	1488	1845	1984	1984	1904	1736	1422
Period (29)	9-10 am	1625	2014	2166	2166	2079	1843	1510
Period (29)	10-11am	1693	2099	2257	2257	2167	1950	1598
Period (29)	11am-12 pm	1710	2120	2280	2280	2189	1993	1633
Period (29)	12-1 pm	1693	2099	2257	2257	2167	1993	1633
Period (29)	1-2 pm	1710	2120	2280	2280	2189	1972	1615

Period (29)	2-3 pm	1710	2120	2280	2280	2189	1950	1598
Period (29)	3-4 pm	1659	2057	2212	2212	2123	1950	1598
Period (29)	4-5 pm	1642	2036	2189	2189	2101	1972	1615
Period (29)	5-6 pm	1642	2036	2189	2189	2101	2015	1650
Period (29)	6-7 pm	1590	1972	2120	2120	2036	2036	1668
Period (29)	7-8 pm	1573	1951	2098	2098	2014	2036	1668
Period (29)	8-9 pm	1573	1951	2098	2098	2014	2143	1756
Period (29)	9-10 pm	1590	1972	2120	2120	2036	1993	1633
Period (29)	10-11 pm	1488	1845	1984	1984	1904	1886	1545
Period (29)	11pm-12am	1231	1527	1642	1642	1576	1715	1404
Period (30)	12-1 am	1205	1494	1606	1606	1542	1746	1430
Period (30)	1-2 am	1130	1401	1506	1506	1446	1652	1353
Period (30)	2-3 am	1092	1354	1456	1456	1398	1557	1276
Period (30)	3-4 am	1054	1307	1406	1406	1349	1534	1256
Period (30)	4-5 am	1054	1307	1406	1406	1349	1510	1237
Period (30)	5-6 am	1092	1354	1456	1456	1398	1463	1198
Period (30)	6-7 am	1205	1494	1606	1606	1542	1463	1198
Period (30)	7-8 am	1431	1774	1908	1908	1831	1557	1276
Period (30)	8-9 am	1638	2031	2184	2184	2096	1911	1565
Period (30)	9-10 am	1788	2218	2385	2385	2289	2029	1662
Period (30)	10-11am	1864	2311	2485	2485	2386	2147	1759
Period (30)	11am-12 pm	1883	2334	2510	2510	2410	2194	1797
Period (30)	12-1 pm	1864	2311	2485	2485	2386	2194	1797
Period (30)	1-2 pm	1883	2334	2510	2510	2410	2171	1778
Period (30)	2-3 pm	1883	2334	2510	2510	2410	2147	1759
Period (30)	3-4 pm	1826	2264	2435	2435	2337	2147	1759
Period (30)	4-5 pm	1807	2241	2410	2410	2313	2171	1778
Period (30)	5-6 pm	1807	2241	2410	2410	2313	2218	1817
Period (30)	6-7 pm	1751	2171	2334	2334	2241	2241	1836
Period (30)	7-8 pm	1732	2148	2309	2309	2217	2241	1836
Period (30)	8-9 pm	1732	2148	2309	2309	2217	2359	1933
Period (30)	9-10 pm	1751	2171	2334	2334	2241	2194	1797
Period (30)	10-11 pm	1638	2031	2184	2184	2096	2076	1701
Period (30)	11pm-12am	1355	1681	1807	1807	1735	1888	1546
Period (31)	12-1 am	973	1207	1298	1298	1246	1452	1190
Period (31)	1-2 am	958	1188	1277	1277	1226	1414	1158
Period	2-3 am	927	1150	1236	1236	1187	1336	1094

(31)								
Period (31)	3-4 am	896	1111	1195	1195	1147	1278	1047
Period (31)	4-5 am	912	1130	1215	1215	1167	1259	1031
Period (31)	5-6 am	1004	1245	1339	1339	1285	1259	1031
Period (31)	6-7 am	1112	1380	1483	1483	1424	1317	1079
Period (31)	7-8 am	1313	1629	1751	1751	1681	1433	1174
Period (31)	8-9 am	1468	1820	1957	1957	1879	1607	1317
Period (31)	9-10 am	1530	1897	2039	2039	1958	1723	1412
Period (31)	10-11am	1545	1916	2060	2060	1978	1781	1459
Period (31)	11am-12 pm	1530	1897	2039	2039	1958	1820	1491
Period (31)	12-1 pm	1437	1782	1916	1916	1839	1762	1443
Period (31)	1-2 pm	1421	1763	1895	1895	1819	1743	1428
Period (31)	2-3 pm	1391	1724	1854	1854	1780	1743	1428
Period (31)	3-4 pm	1360	1686	1813	1813	1740	1665	1364
Period (31)	4-5 pm	1391	1724	1854	1854	1780	1646	1348
Period (31)	5-6 pm	1421	1763	1895	1895	1819	1704	1396
Period (31)	6-7 pm	1483	1839	1978	1978	1898	1781	1459
Period (31)	7-8 pm	1514	1878	2019	2019	1938	1936	1586
Period (31)	8-9 pm	1483	1839	1978	1978	1898	1878	1539
Period (31)	9-10 pm	1391	1724	1854	1854	1780	1840	1507
Period (31)	10-11 pm	1236	1533	1648	1648	1582	1743	1428
Period (31)	11pm-12am	1082	1341	1442	1442	1384	1646	1348
Period (32)	12-1 am	1044	1295	1392	1392	1337	1558	1276
Period (32)	1-2 am	1028	1274	1370	1370	1315	1517	1242
Period (32)	2-3 am	995	1233	1326	1326	1273	1433	1174
Period (32)	3-4 am	961	1192	1282	1282	1231	1371	1123
Period (32)	4-5 am	978	1212	1304	1304	1252	1350	1106
Period (32)	5-6 am	1077	1336	1437	1437	1379	1350	1106
Period (32)	6-7 am	1193	1480	1591	1591	1528	1413	1157
Period (32)	7-8 am	1409	1747	1879	1879	1803	1537	1259
Period (32)	8-9 am	1575	1952	2100	2100	2016	1724	1412
Period (32)	9-10 am	1641	2034	2188	2188	2100	1849	1515
Period (32)	10-11am	1658	2055	2210	2210	2122	1911	1566
Period (32)	11am-12 pm	1641	2034	2188	2188	2100	1953	1600
Period (32)	12-1 pm	1541	1911	2055	2055	1973	1890	1549
Period (32)	1-2 pm	1525	1891	2033	2033	1952	1870	1532
Period (32)	2-3 pm	1492	1850	1989	1989	1909	1870	1532

Period (32)	3-4 pm	1459	1808	1945	1945	1867	1787	1463
Period (32)	4-5 pm	1492	1850	1989	1989	1909	1766	1446
Period (32)	5-6 pm	1525	1891	2033	2033	1952	1828	1497
Period (32)	6-7 pm	1591	1973	2122	2122	2037	1911	1566
Period (32)	7-8 pm	1624	2014	2166	2166	2079	2077	1702
Period (32)	8-9 pm	1591	1973	2122	2122	2037	2015	1651
Period (32)	9-10 pm	1492	1850	1989	1989	1909	1974	1617
Period (32)	10-11 pm	1326	1644	1768	1768	1697	1870	1532
Period (32)	11pm-12am	1160	1439	1547	1547	1485	1766	1446
Period (33)	12-1 am	1077	1336	1436	1436	1379	1607	1317
Period (33)	1-2 am	1060	1314	1414	1414	1357	1565	1282
Period (33)	2-3 am	1026	1272	1368	1368	1313	1479	1211
Period (33)	3-4 am	992	1230	1322	1322	1270	1415	1159
Period (33)	4-5 am	1009	1251	1345	1345	1291	1393	1141
Period (33)	5-6 am	1112	1378	1482	1482	1423	1393	1141
Period (33)	6-7 am	1231	1526	1642	1642	1576	1457	1194
Period (33)	7-8 am	1454	1802	1938	1938	1860	1586	1299
Period (33)	8-9 am	1625	2014	2166	2166	2079	1779	1457
Period (33)	9-10 am	1693	2099	2257	2257	2167	1907	1562
Period (33)	10-11am	1710	2120	2280	2280	2189	1972	1615
Period (33)	11am-12 pm	1693	2099	2257	2257	2167	2015	1650
Period (33)	12-1 pm	1590	1972	2120	2120	2036	1950	1598
Period (33)	1-2 pm	1573	1950	2098	2098	2014	1929	1580
Period (33)	2-3 pm	1539	1908	2052	2052	1970	1929	1580
Period (33)	3-4 pm	1505	1866	2006	2006	1926	1843	1510
Period (33)	4-5 pm	1539	1908	2052	2052	1970	1822	1492
Period (33)	5-6 pm	1573	1950	2098	2098	2014	1886	1545
Period (33)	6-7 pm	1642	2035	2189	2189	2101	1972	1615
Period (33)	7-8 pm	1676	2078	2234	2234	2145	2143	1756
Period (33)	8-9 pm	1642	2035	2189	2189	2101	2079	1703
Period (33)	9-10 pm	1539	1908	2052	2052	1970	2036	1668
Period (33)	10-11 pm	1368	1696	1824	1824	1751	1929	1580
Period (33)	11pm-12am	1197	1484	1596	1596	1532	1822	1492
Period (34)	12-1 am	983	1218	1310	1310	1258	1466	1201
Period (34)	1-2 am	967	1199	1290	1290	1238	1427	1169
Period (34)	2-3 am	936	1160	1248	1248	1198	1349	1105
Period	3-4 am	905	1122	1206	1206	1158	1290	1057

(34)								
Period (34)	4-5 am	920	1141	1227	1227	1178	1271	1041
Period (34)	5-6 am	1014	1257	1352	1352	1298	1271	1041
Period (34)	6-7 am	1123	1392	1498	1498	1438	1329	1089
Period (34)	7-8 am	1326	1644	1768	1768	1697	1447	1185
Period (34)	8-9 am	1482	1837	1976	1976	1897	1623	1329
Period (34)	9-10 am	1544	1915	2059	2059	1977	1740	1425
Period (34)	10-11am	1560	1934	2080	2080	1997	1799	1473
Period (34)	11am-12 pm	1544	1915	2059	2059	1977	1838	1506
Period (34)	12-1 pm	1451	1799	1934	1934	1857	1779	1457
Period (34)	1-2 pm	1435	1779	1914	1914	1837	1760	1441
Period (34)	2-3 pm	1404	1741	1872	1872	1797	1760	1441
Period (34)	3-4 pm	1373	1702	1830	1830	1757	1681	1377
Period (34)	4-5 pm	1404	1741	1872	1872	1797	1662	1361
Period (34)	5-6 pm	1435	1779	1914	1914	1837	1720	1409
Period (34)	6-7 pm	1498	1857	1997	1997	1917	1799	1473
Period (34)	7-8 pm	1529	1895	2038	2038	1957	1955	1602
Period (34)	8-9 pm	1498	1857	1997	1997	1917	1896	1554
Period (34)	9-10 pm	1404	1741	1872	1872	1797	1857	1522
Period (34)	10-11 pm	1248	1547	1664	1664	1597	1760	1441
Period (34)	11pm-12am	1092	1354	1456	1456	1398	1662	1361
Period (35)	12-1 am	978	1213	1304	1304	1252	1460	1195
Period (35)	1-2 am	963	1194	1283	1283	1232	1421	1164
Period (35)	2-3 am	932	1155	1242	1242	1192	1343	1100
Period (35)	3-4 am	900	1117	1201	1201	1153	1284	1052
Period (35)	4-5 am	916	1136	1221	1221	1172	1265	1036
Period (35)	5-6 am	1009	1251	1346	1346	1292	1265	1036
Period (35)	6-7 am	1118	1386	1490	1490	1431	1323	1084
Period (35)	7-8 am	1320	1636	1760	1760	1689	1440	1179
Period (35)	8-9 am	1475	1829	1967	1967	1888	1615	1323
Period (35)	9-10 am	1537	1906	2049	2049	1967	1732	1419
Period (35)	10-11am	1553	1925	2070	2070	1987	1790	1466
Period (35)	11am-12 pm	1537	1906	2049	2049	1967	1829	1498
Period (35)	12-1 pm	1444	1790	1925	1925	1848	1771	1450
Period (35)	1-2 pm	1428	1771	1904	1904	1828	1751	1435
Period (35)	2-3 pm	1397	1733	1863	1863	1788	1751	1435
Period (35)	3-4 pm	1366	1694	1822	1822	1749	1674	1371

Period (35)	4-5 pm	1397	1733	1863	1863	1788	1654	1355
Period (35)	5-6 pm	1428	1771	1904	1904	1828	1712	1403
Period (35)	6-7 pm	1490	1848	1987	1987	1908	1790	1466
Period (35)	7-8 pm	1521	1887	2029	2029	1947	1946	1594
Period (35)	8-9 pm	1490	1848	1987	1987	1908	1888	1546
Period (35)	9-10 pm	1397	1733	1863	1863	1788	1849	1514
Period (35)	10-11 pm	1242	1540	1656	1656	1590	1751	1435
Period (35)	11pm-12am	1087	1348	1449	1449	1391	1654	1355
Period (36)	12-1 am	950	1177	1266	1266	1216	1417	1161
Period (36)	1-2 am	935	1159	1246	1246	1196	1379	1130
Period (36)	2-3 am	905	1121	1206	1206	1158	1303	1068
Period (36)	3-4 am	874	1084	1166	1166	1119	1247	1021
Period (36)	4-5 am	889	1103	1186	1186	1138	1228	1006
Period (36)	5-6 am	980	1215	1307	1307	1254	1228	1006
Period (36)	6-7 am	1085	1346	1447	1447	1389	1285	1052
Period (36)	7-8 am	1281	1589	1709	1709	1640	1398	1145
Period (36)	8-9 am	1432	1776	1910	1910	1833	1568	1285
Period (36)	9-10 am	1492	1850	1990	1990	1910	1681	1377
Period (36)	10-11am	1508	1869	2010	2010	1930	1738	1424
Period (36)	11am-12 pm	1492	1850	1990	1990	1910	1776	1455
Period (36)	12-1 pm	1402	1738	1869	1869	1795	1719	1408
Period (36)	1-2 pm	1387	1719	1849	1849	1775	1700	1393
Period (36)	2-3 pm	1357	1682	1809	1809	1737	1700	1393
Period (36)	3-4 pm	1327	1645	1769	1769	1698	1625	1331
Period (36)	4-5 pm	1357	1682	1809	1809	1737	1606	1316
Period (36)	5-6 pm	1387	1719	1849	1849	1775	1662	1362
Period (36)	6-7 pm	1447	1794	1930	1930	1852	1738	1424
Period (36)	7-8 pm	1477	1832	1970	1970	1891	1889	1548
Period (36)	8-9 pm	1447	1794	1930	1930	1852	1832	1501
Period (36)	9-10 pm	1357	1682	1809	1809	1737	1795	1470
Period (36)	10-11 pm	1206	1495	1608	1608	1544	1700	1393
Period (36)	11pm-12am	1055	1308	1407	1407	1351	1606	1316
Period (37)	12-1 am	1049	1301	1399	1399	1343	1565	1282
Period (37)	1-2 am	1032	1280	1376	1376	1321	1524	1248
Period (37)	2-3 am	999	1239	1332	1332	1279	1440	1179
Period (37)	3-4 am	966	1198	1288	1288	1236	1377	1128
Period	4-5 am	982	1218	1310	1310	1257	1357	1111

(37)								
Period (37)	5-6 am	1082	1342	1443	1443	1385	1357	1111
Period (37)	6-7 am	1199	1487	1598	1598	1534	1419	1162
Period (37)	7-8 am	1415	1755	1887	1887	1812	1544	1265
Period (37)	8-9 am	1582	1962	2109	2109	2025	1732	1419
Period (37)	9-10 am	1648	2044	2198	2198	2110	1857	1521
Period (37)	10-11am	1665	2065	2220	2220	2131	1920	1573
Period (37)	11am-12 pm	1648	2044	2198	2198	2110	1962	1607
Period (37)	12-1 pm	1548	1920	2065	2065	1982	1899	1556
Period (37)	1-2 pm	1532	1900	2042	2042	1961	1878	1538
Period (37)	2-3 pm	1499	1859	1998	1998	1918	1878	1538
Period (37)	3-4 pm	1465	1817	1954	1954	1875	1795	1470
Period (37)	4-5 pm	1499	1859	1998	1998	1918	1774	1453
Period (37)	5-6 pm	1532	1900	2042	2042	1961	1837	1504
Period (37)	6-7 pm	1598	1982	2131	2131	2046	1920	1573
Period (37)	7-8 pm	1632	2024	2176	2176	2089	2087	1709
Period (37)	8-9 pm	1598	1982	2131	2131	2046	2024	1658
Period (37)	9-10 pm	1499	1859	1998	1998	1918	1983	1624
Period (37)	10-11 pm	1332	1652	1776	1776	1705	1878	1538
Period (37)	11pm-12am	1166	1446	1554	1554	1492	1774	1453
Period (38)	12-1 am	936	1160	1247	1247	1198	1396	1143
Period (38)	1-2 am	921	1141	1228	1228	1178	1359	1113
Period (38)	2-3 am	891	1105	1188	1188	1140	1284	1052
Period (38)	3-4 am	861	1068	1148	1148	1102	1228	1006
Period (38)	4-5 am	876	1086	1168	1168	1121	1210	991
Period (38)	5-6 am	965	1197	1287	1287	1236	1210	991
Period (38)	6-7 am	1069	1326	1426	1426	1369	1265	1037
Period (38)	7-8 am	1262	1565	1683	1683	1616	1377	1128
Period (38)	8-9 am	1411	1749	1881	1881	1806	1545	1265
Period (38)	9-10 am	1470	1823	1960	1960	1882	1656	1357
Period (38)	10-11am	1485	1841	1980	1980	1901	1712	1403
Period (38)	11am-12 pm	1470	1823	1960	1960	1882	1749	1433
Period (38)	12-1 pm	1381	1712	1841	1841	1768	1694	1387
Period (38)	1-2 pm	1366	1694	1822	1822	1749	1675	1372
Period (38)	2-3 pm	1337	1657	1782	1782	1711	1675	1372
Period (38)	3-4 pm	1307	1620	1742	1742	1673	1600	1311
Period (38)	4-5 pm	1337	1657	1782	1782	1711	1582	1296

Period (38)	5-6 pm	1366	1694	1822	1822	1749	1638	1342
Period (38)	6-7 pm	1426	1767	1901	1901	1825	1712	1403
Period (38)	7-8 pm	1455	1804	1940	1940	1863	1861	1525
Period (38)	8-9 pm	1426	1767	1901	1901	1825	1805	1479
Period (38)	9-10 pm	1337	1657	1782	1782	1711	1768	1448
Period (38)	10-11 pm	1188	1473	1584	1584	1521	1675	1372
Period (38)	11pm-12am	1040	1289	1386	1386	1331	1582	1296
Period (39)	12-1 am	973	1207	1298	1298	1246	1452	1190
Period (39)	1-2 am	958	1188	1277	1277	1226	1413	1158
Period (39)	2-3 am	927	1150	1236	1236	1187	1336	1094
Period (39)	3-4 am	896	1111	1195	1195	1147	1278	1047
Period (39)	4-5 am	912	1130	1215	1215	1167	1258	1031
Period (39)	5-6 am	1004	1245	1339	1339	1285	1258	1031
Period (39)	6-7 am	1112	1380	1483	1483	1424	1316	1079
Period (39)	7-8 am	1313	1629	1751	1751	1681	1433	1174
Period (39)	8-9 am	1468	1820	1957	1957	1879	1607	1317
Period (39)	9-10 am	1530	1897	2039	2039	1958	1723	1412
Period (39)	10-11am	1545	1916	2060	2060	1978	1781	1459
Period (39)	11am-12 pm	1530	1897	2039	2039	1958	1820	1491
Period (39)	12-1 pm	1437	1782	1916	1916	1839	1762	1443
Period (39)	1-2 pm	1421	1763	1895	1895	1819	1742	1428
Period (39)	2-3 pm	1391	1724	1854	1854	1780	1742	1428
Period (39)	3-4 pm	1360	1686	1813	1813	1740	1665	1364
Period (39)	4-5 pm	1391	1724	1854	1854	1780	1646	1348
Period (39)	5-6 pm	1421	1763	1895	1895	1819	1704	1396
Period (39)	6-7 pm	1483	1839	1978	1978	1898	1781	1459
Period (39)	7-8 pm	1514	1878	2019	2019	1938	1936	1586
Period (39)	8-9 pm	1483	1839	1978	1978	1898	1878	1539
Period (39)	9-10 pm	1391	1724	1854	1854	1780	1839	1507
Period (39)	10-11 pm	1236	1533	1648	1648	1582	1742	1428
Period (39)	11pm-12am	1082	1341	1442	1442	1384	1646	1348
Period (40)	12-1 am	973	1207	1298	1298	1246	1452	1190
Period (40)	1-2 am	958	1188	1277	1277	1226	1413	1158
Period (40)	2-3 am	927	1150	1236	1236	1187	1336	1094
Period (40)	3-4 am	896	1111	1195	1195	1147	1278	1047
Period (40)	4-5 am	912	1130	1215	1215	1167	1258	1031
Period	5-6 am	1004	1245	1339	1339	1285	1258	1031

(40)								
Period (40)	6-7 am	1112	1380	1483	1483	1424	1316	1079
Period (40)	7-8 am	1313	1629	1751	1751	1681	1433	1174
Period (40)	8-9 am	1468	1820	1957	1957	1879	1607	1317
Period (40)	9-10 am	1530	1897	2039	2039	1958	1723	1412
Period (40)	10-11am	1545	1916	2060	2060	1978	1781	1459
Period (40)	11am-12 pm	1530	1897	2039	2039	1958	1820	1491
Period (40)	12-1 pm	1437	1782	1916	1916	1839	1762	1443
Period (40)	1-2 pm	1421	1763	1895	1895	1819	1742	1428
Period (40)	2-3 pm	1391	1724	1854	1854	1780	1742	1428
Period (40)	3-4 pm	1360	1686	1813	1813	1740	1665	1364
Period (40)	4-5 pm	1391	1724	1854	1854	1780	1646	1348
Period (40)	5-6 pm	1421	1763	1895	1895	1819	1704	1396
Period (40)	6-7 pm	1483	1839	1978	1978	1898	1781	1459
Period (40)	7-8 pm	1514	1878	2019	2019	1938	1936	1586
Period (40)	8-9 pm	1483	1839	1978	1978	1898	1878	1539
Period (40)	9-10 pm	1391	1724	1854	1854	1780	1839	1507
Period (40)	10-11 pm	1236	1533	1648	1648	1582	1742	1428
Period (40)	11pm-12am	1082	1341	1442	1442	1384	1646	1348
Period (41)	12-1 am	1002	1242	1336	1336	1282	1495	1224
Period (41)	1-2 am	986	1223	1314	1314	1262	1455	1192
Period (41)	2-3 am	954	1183	1272	1272	1221	1375	1126
Period (41)	3-4 am	922	1144	1230	1230	1180	1315	1077
Period (41)	4-5 am	938	1163	1251	1251	1201	1295	1061
Period (41)	5-6 am	1034	1282	1378	1378	1323	1295	1061
Period (41)	6-7 am	1145	1420	1526	1526	1465	1355	1110
Period (41)	7-8 am	1352	1676	1802	1802	1730	1475	1208
Period (41)	8-9 am	1511	1873	2014	2014	1933	1654	1355
Period (41)	9-10 am	1574	1952	2099	2099	2015	1774	1453
Period (41)	10-11am	1590	1972	2120	2120	2035	1834	1502
Period (41)	11am-12 pm	1574	1952	2099	2099	2015	1873	1534
Period (41)	12-1 pm	1479	1834	1972	1972	1893	1814	1485
Period (41)	1-2 pm	1463	1814	1950	1950	1872	1794	1469
Period (41)	2-3 pm	1431	1775	1908	1908	1832	1794	1469
Period (41)	3-4 pm	1399	1735	1866	1866	1791	1714	1404
Period (41)	4-5 pm	1431	1775	1908	1908	1832	1694	1388
Period (41)	5-6 pm	1463	1814	1950	1950	1872	1754	1437

Period (41)	6-7 pm	1526	1893	2035	2035	1954	1834	1502
Period (41)	7-8 pm	1558	1933	2078	2078	1994	1993	1632
Period (41)	8-9 pm	1526	1893	2035	2035	1954	1933	1583
Period (41)	9-10 pm	1431	1775	1908	1908	1832	1893	1551
Period (41)	10-11 pm	1272	1578	1696	1696	1628	1794	1469
Period (41)	11pm-12am	1113	1380	1484	1484	1425	1694	1388
Period (42)	12-1 am	1002	1242	1336	1336	1282	1495	1224
Period (42)	1-2 am	986	1223	1314	1314	1262	1455	1192
Period (42)	2-3 am	954	1183	1272	1272	1221	1375	1126
Period (42)	3-4 am	922	1144	1230	1230	1180	1315	1077
Period (42)	4-5 am	938	1163	1251	1251	1201	1295	1061
Period (42)	5-6 am	1034	1282	1378	1378	1323	1295	1061
Period (42)	6-7 am	1145	1420	1526	1526	1465	1355	1110
Period (42)	7-8 am	1352	1676	1802	1802	1730	1475	1208
Period (42)	8-9 am	1511	1873	2014	2014	1933	1654	1355
Period (42)	9-10 am	1574	1952	2099	2099	2015	1774	1453
Period (42)	10-11am	1590	1972	2120	2120	2035	1834	1502
Period (42)	11am-12 pm	1574	1952	2099	2099	2015	1873	1534
Period (42)	12-1 pm	1479	1834	1972	1972	1893	1814	1485
Period (42)	1-2 pm	1463	1814	1950	1950	1872	1794	1469
Period (42)	2-3 pm	1431	1775	1908	1908	1832	1794	1469
Period (42)	3-4 pm	1399	1735	1866	1866	1791	1714	1404
Period (42)	4-5 pm	1431	1775	1908	1908	1832	1694	1388
Period (42)	5-6 pm	1463	1814	1950	1950	1872	1754	1437
Period (42)	6-7 pm	1526	1893	2035	2035	1954	1834	1502
Period (42)	7-8 pm	1558	1933	2078	2078	1994	1993	1632
Period (42)	8-9 pm	1526	1893	2035	2035	1954	1933	1583
Period (42)	9-10 pm	1431	1775	1908	1908	1832	1893	1551
Period (42)	10-11 pm	1272	1578	1696	1696	1628	1794	1469
Period (42)	11pm-12am	1113	1380	1484	1484	1425	1694	1388
Period (43)	12-1 am	1077	1336	1436	1436	1379	1607	1317
Period (43)	1-2 am	1060	1314	1414	1414	1357	1564	1282
Period (43)	2-3 am	1026	1272	1368	1368	1313	1479	1211
Period (43)	3-4 am	992	1230	1322	1322	1270	1414	1159
Period (43)	4-5 am	1009	1251	1345	1345	1291	1393	1141
Period (43)	5-6 am	1112	1378	1482	1482	1423	1393	1141
Period	6-7 am	1231	1526	1642	1642	1576	1457	1194

(43)								
Period (43)	7-8 am	1454	1802	1938	1938	1860	1586	1299
Period (43)	8-9 am	1625	2014	2166	2166	2079	1779	1457
Period (43)	9-10 am	1693	2099	2257	2257	2167	1907	1562
Period (43)	10-11am	1710	2120	2280	2280	2189	1972	1615
Period (43)	11am-12 pm	1693	2099	2257	2257	2167	2014	1650
Period (43)	12-1 pm	1590	1972	2120	2120	2036	1950	1598
Period (43)	1-2 pm	1573	1950	2098	2098	2014	1929	1580
Period (43)	2-3 pm	1539	1908	2052	2052	1970	1929	1580
Period (43)	3-4 pm	1505	1866	2006	2006	1926	1843	1510
Period (43)	4-5 pm	1539	1908	2052	2052	1970	1822	1492
Period (43)	5-6 pm	1573	1950	2098	2098	2014	1886	1545
Period (43)	6-7 pm	1642	2035	2189	2189	2101	1972	1615
Period (43)	7-8 pm	1676	2078	2234	2234	2145	2143	1756
Period (43)	8-9 pm	1642	2035	2189	2189	2101	2079	1703
Period (43)	9-10 pm	1539	1908	2052	2052	1970	2036	1668
Period (43)	10-11 pm	1368	1696	1824	1824	1751	1929	1580
Period (43)	11pm-12am	1197	1484	1596	1596	1532	1822	1492
Period (44)	12-1 am	1261	1564	1682	1682	1614	1840	1508
Period (44)	1-2 am	1186	1471	1581	1581	1518	1699	1392
Period (44)	2-3 am	1130	1401	1506	1506	1446	1604	1314
Period (44)	3-4 am	1111	1377	1481	1481	1422	1557	1276
Period (44)	4-5 am	1111	1377	1481	1481	1422	1510	1237
Period (44)	5-6 am	1130	1401	1506	1506	1446	1534	1256
Period (44)	6-7 am	1393	1727	1857	1857	1783	1557	1276
Period (44)	7-8 am	1619	2007	2159	2159	2072	1652	1353
Period (44)	8-9 am	1788	2218	2385	2385	2289	1888	1546
Period (44)	9-10 am	1807	2241	2410	2410	2313	2076	1701
Period (44)	10-11am	1807	2241	2410	2410	2313	2123	1739
Period (44)	11am-12 pm	1788	2218	2385	2385	2289	2147	1759
Period (44)	12-1 pm	1788	2218	2385	2385	2289	2123	1739
Period (44)	1-2 pm	1788	2218	2385	2385	2289	2076	1701
Period (44)	2-3 pm	1751	2171	2334	2334	2241	2053	1681
Period (44)	3-4 pm	1770	2194	2359	2359	2265	2053	1681
Period (44)	4-5 pm	1864	2311	2485	2485	2386	2147	1759
Period (44)	5-6 pm	1883	2334	2510	2510	2410	2359	1933
Period (44)	6-7 pm	1883	2334	2510	2510	2410	2336	1913

Period (44)	7-8 pm	1807	2241	2410	2410	2313	2289	1875
Period (44)	8-9 pm	1713	2124	2284	2284	2193	2218	1817
Period (44)	9-10 pm	1562	1937	2083	2083	2000	2171	1778
Period (44)	10-11 pm	1374	1704	1832	1832	1759	2053	1681
Period (44)	11pm-12am	1186	1471	1581	1581	1518	1911	1565
Period (45)	12-1 am	1266	1570	1682	1682	1621	1848	1514
Period (45)	1-2 am	1191	1476	1581	1581	1524	1706	1397
Period (45)	2-3 am	1134	1406	1506	1506	1452	1611	1319
Period (45)	3-4 am	1115	1383	1481	1481	1427	1563	1281
Period (45)	4-5 am	1115	1383	1481	1481	1427	1516	1242
Period (45)	5-6 am	1134	1406	1506	1506	1452	1540	1261
Period (45)	6-7 am	1399	1734	1857	1857	1790	1563	1281
Period (45)	7-8 am	1625	2015	2159	2159	2081	1658	1358
Period (45)	8-9 am	1796	2226	2385	2385	2298	1895	1552
Period (45)	9-10 am	1814	2250	2410	2410	2322	2085	1708
Period (45)	10-11am	1814	2250	2410	2410	2322	2132	1746
Period (45)	11am-12 pm	1796	2226	2385	2385	2298	2156	1766
Period (45)	12-1 pm	1796	2226	2385	2385	2298	2132	1746
Period (45)	1-2 pm	1796	2226	2385	2385	2298	2085	1708
Period (45)	2-3 pm	1758	2180	2334	2334	2250	2061	1688
Period (45)	3-4 pm	1777	2203	2359	2359	2274	2061	1688
Period (45)	4-5 pm	1871	2320	2485	2485	2395	2156	1766
Period (45)	5-6 pm	1890	2344	2510	2510	2419	2369	1940
Period (45)	6-7 pm	1890	2344	2510	2510	2419	2345	1921
Period (45)	7-8 pm	1814	2250	2410	2410	2322	2298	1882
Period (45)	8-9 pm	1720	2133	2284	2284	2201	2227	1824
Period (45)	9-10 pm	1569	1945	2083	2083	2008	2179	1785
Period (45)	10-11 pm	1380	1711	1832	1832	1766	2061	1688
Period (45)	11pm-12am	1191	1476	1581	1581	1524	1919	1572
Period (46)	12-1 am	1301	1614	1735	1735	1666	1899	1556
Period (46)	1-2 am	1224	1517	1632	1632	1566	1753	1436
Period (46)	2-3 am	1166	1445	1554	1554	1492	1656	1356
Period (46)	3-4 am	1146	1421	1528	1528	1467	1607	1316
Period (46)	4-5 am	1146	1421	1528	1528	1467	1558	1276
Period (46)	5-6 am	1166	1445	1554	1554	1492	1582	1296
Period (46)	6-7 am	1437	1782	1917	1917	1840	1607	1316
Period	7-8 am	1671	2071	2227	2227	2138	1704	1396

(46)								
Period (46)	8-9 am	1845	2288	2461	2461	2362	1948	1595
Period (46)	9-10 am	1865	2312	2486	2486	2387	2142	1755
Period (46)	10-11am	1865	2312	2486	2486	2387	2191	1795
Period (46)	11am-12 pm	1845	2288	2461	2461	2362	2215	1815
Period (46)	12-1 pm	1845	2288	2461	2461	2362	2191	1795
Period (46)	1-2 pm	1845	2288	2461	2461	2362	2142	1755
Period (46)	2-3 pm	1807	2240	2409	2409	2312	2118	1735
Period (46)	3-4 pm	1826	2264	2435	2435	2337	2118	1735
Period (46)	4-5 pm	1923	2385	2564	2564	2462	2215	1815
Period (46)	5-6 pm	1943	2409	2590	2590	2486	2435	1994
Period (46)	6-7 pm	1943	2409	2590	2590	2486	2410	1974
Period (46)	7-8 pm	1865	2312	2486	2486	2387	2362	1934
Period (46)	8-9 pm	1768	2192	2357	2357	2263	2289	1875
Period (46)	9-10 pm	1612	1999	2150	2150	2064	2240	1835
Period (46)	10-11 pm	1418	1758	1891	1891	1815	2118	1735
Period (46)	11pm-12am	1224	1517	1632	1632	1566	1972	1615
Period (47)	12-1 am	1347	1670	1796	1796	1724	1965	1610
Period (47)	1-2 am	1266	1570	1688	1688	1621	1814	1486
Period (47)	2-3 am	1206	1495	1608	1608	1544	1713	1403
Period (47)	3-4 am	1186	1471	1581	1581	1518	1663	1362
Period (47)	4-5 am	1186	1471	1581	1581	1518	1612	1321
Period (47)	5-6 am	1206	1495	1608	1608	1544	1637	1341
Period (47)	6-7 am	1487	1844	1983	1983	1904	1663	1362
Period (47)	7-8 am	1729	2143	2305	2305	2213	1763	1445
Period (47)	8-9 am	1910	2368	2546	2546	2444	2015	1651
Period (47)	9-10 am	1930	2393	2573	2573	2470	2217	1816
Period (47)	10-11am	1930	2393	2573	2573	2470	2267	1857
Period (47)	11am-12 pm	1910	2368	2546	2546	2444	2292	1878
Period (47)	12-1 pm	1910	2368	2546	2546	2444	2267	1857
Period (47)	1-2 pm	1910	2368	2546	2546	2444	2217	1816
Period (47)	2-3 pm	1869	2318	2492	2492	2393	2192	1795
Period (47)	3-4 pm	1889	2343	2519	2519	2418	2192	1795
Period (47)	4-5 pm	1990	2467	2653	2653	2547	2292	1878
Period (47)	5-6 pm	2010	2492	2680	2680	2573	2519	2064
Period (47)	6-7 pm	2010	2492	2680	2680	2573	2494	2043
Period (47)	7-8 pm	1930	2393	2573	2573	2470	2444	2002

Period (47)	8-9 pm	1829	2268	2439	2439	2341	2368	1940
Period (47)	9-10 pm	1668	2069	2224	2224	2135	2318	1899
Period (47)	10-11 pm	1467	1819	1956	1956	1878	2192	1795
Period (47)	11pm-12am	1266	1570	1688	1688	1621	2041	1672
Period (48)	12-1 am	1276	1583	1702	1702	1634	1862	1526
Period (48)	1-2 am	1200	1488	1600	1600	1536	1719	1408
Period (48)	2-3 am	1143	1417	1524	1524	1463	1624	1330
Period (48)	3-4 am	1124	1394	1499	1499	1439	1576	1291
Period (48)	4-5 am	1124	1394	1499	1499	1439	1528	1252
Period (48)	5-6 am	1143	1417	1524	1524	1463	1552	1271
Period (48)	6-7 am	1410	1748	1880	1880	1804	1576	1291
Period (48)	7-8 am	1638	2031	2184	2184	2097	1671	1369
Period (48)	8-9 am	1810	2244	2413	2413	2316	1910	1565
Period (48)	9-10 am	1829	2268	2438	2438	2341	2101	1721
Period (48)	10-11am	1829	2268	2438	2438	2341	2149	1760
Period (48)	11am-12 pm	1810	2244	2413	2413	2316	2173	1780
Period (48)	12-1 pm	1810	2244	2413	2413	2316	2149	1760
Period (48)	1-2 pm	1810	2244	2413	2413	2316	2101	1721
Period (48)	2-3 pm	1772	2197	2362	2362	2268	2077	1702
Period (48)	3-4 pm	1791	2220	2388	2388	2292	2077	1702
Period (48)	4-5 pm	1886	2339	2515	2515	2414	2173	1780
Period (48)	5-6 pm	1905	2362	2540	2540	2438	2388	1956
Period (48)	6-7 pm	1905	2362	2540	2540	2438	2364	1936
Period (48)	7-8 pm	1829	2268	2438	2438	2341	2316	1897
Period (48)	8-9 pm	1734	2150	2311	2311	2219	2244	1838
Period (48)	9-10 pm	1581	1961	2108	2108	2024	2197	1799
Period (48)	10-11 pm	1391	1724	1854	1854	1780	2077	1702
Period (48)	11pm-12am	1200	1488	1600	1600	1536	1934	1584
Period (49)	12-1 am	1347	1670	1796	1796	1724	1965	1610
Period (49)	1-2 am	1266	1570	1688	1688	1621	1814	1486
Period (49)	2-3 am	1206	1495	1608	1608	1544	1713	1403
Period (49)	3-4 am	1186	1471	1581	1581	1518	1663	1362
Period (49)	4-5 am	1186	1471	1581	1581	1518	1612	1321
Period (49)	5-6 am	1206	1495	1608	1608	1544	1637	1341
Period (49)	6-7 am	1487	1844	1983	1983	1904	1663	1362
Period (49)	7-8 am	1729	2143	2305	2305	2213	1763	1445
Period	8-9 am	1910	2368	2546	2546	2444	2015	1651

(49)								
Period (49)	9-10 am	1930	2393	2573	2573	2470	2217	1816
Period (49)	10-11am	1930	2393	2573	2573	2470	2267	1857
Period (49)	11am-12 pm	1910	2368	2546	2546	2444	2292	1878
Period (49)	12-1 pm	1910	2368	2546	2546	2444	2267	1857
Period (49)	1-2 pm	1910	2368	2546	2546	2444	2217	1816
Period (49)	2-3 pm	1869	2318	2492	2492	2393	2192	1795
Period (49)	3-4 pm	1889	2343	2519	2519	2418	2192	1795
Period (49)	4-5 pm	1990	2467	2653	2653	2547	2292	1878
Period (49)	5-6 pm	2010	2492	2680	2680	2573	2519	2064
Period (49)	6-7 pm	2010	2492	2680	2680	2573	2494	2043
Period (49)	7-8 pm	1930	2393	2573	2573	2470	2444	2002
Period (49)	8-9 pm	1829	2268	2439	2439	2341	2368	1940
Period (49)	9-10 pm	1668	2069	2224	2224	2135	2318	1899
Period (49)	10-11 pm	1467	1819	1956	1956	1878	2192	1795
Period (49)	11pm-12am	1266	1570	1688	1688	1621	2041	1672
Period (50)	12-1 am	1387	1720	1849	1849	1775	2024	1658
Period (50)	1-2 am	1304	1617	1739	1739	1669	1868	1530
Period (50)	2-3 am	1242	1540	1656	1656	1590	1764	1445
Period (50)	3-4 am	1221	1514	1628	1628	1563	1712	1403
Period (50)	4-5 am	1221	1514	1628	1628	1563	1660	1360
Period (50)	5-6 am	1242	1540	1656	1656	1590	1686	1381
Period (50)	6-7 am	1532	1899	2042	2042	1961	1712	1403
Period (50)	7-8 am	1780	2207	2374	2374	2279	1816	1488
Period (50)	8-9 am	1967	2438	2622	2622	2517	2076	1700
Period (50)	9-10 am	1987	2464	2650	2650	2544	2283	1870
Period (50)	10-11am	1987	2464	2650	2650	2544	2335	1913
Period (50)	11am-12 pm	1967	2438	2622	2622	2517	2361	1934
Period (50)	12-1 pm	1967	2438	2622	2622	2517	2335	1913
Period (50)	1-2 pm	1967	2438	2622	2622	2517	2283	1870
Period (50)	2-3 pm	1925	2387	2567	2567	2464	2257	1849
Period (50)	3-4 pm	1946	2413	2594	2594	2491	2257	1849
Period (50)	4-5 pm	2049	2541	2732	2732	2623	2361	1934
Period (50)	5-6 pm	2070	2567	2760	2760	2650	2594	2125
Period (50)	6-7 pm	2070	2567	2760	2760	2650	2568	2104
Period (50)	7-8 pm	1987	2464	2650	2650	2544	2517	2061
Period (50)	8-9 pm	1884	2336	2512	2512	2411	2439	1998

Period (50)	9-10 pm	1718	2130	2291	2291	2199	2387	1955
Period (50)	10-11 pm	1511	1874	2015	2015	1934	2257	1849
Period (50)	11pm-12am	1304	1617	1739	1739	1669	2101	1721
Period (51)	12-1 am	1432	1776	1910	1910	1833	2090	1712
Period (51)	1-2 am	1347	1670	1796	1796	1724	1929	1580
Period (51)	2-3 am	1283	1590	1710	1710	1642	1822	1492
Period (51)	3-4 am	1261	1564	1682	1682	1614	1768	1448
Period (51)	4-5 am	1261	1564	1682	1682	1614	1715	1404
Period (51)	5-6 am	1283	1590	1710	1710	1642	1741	1426
Period (51)	6-7 am	1582	1961	2109	2109	2025	1768	1448
Period (51)	7-8 am	1838	2279	2451	2451	2353	1875	1536
Period (51)	8-9 am	2031	2518	2708	2708	2599	2143	1756
Period (51)	9-10 am	2052	2544	2736	2736	2627	2358	1931
Period (51)	10-11am	2052	2544	2736	2736	2627	2411	1975
Period (51)	11am-12 pm	2031	2518	2708	2708	2599	2438	1997
Period (51)	12-1 pm	2031	2518	2708	2708	2599	2411	1975
Period (51)	1-2 pm	2031	2518	2708	2708	2599	2358	1931
Period (51)	2-3 pm	1988	2465	2651	2651	2544	2331	1909
Period (51)	3-4 pm	2009	2491	2679	2679	2572	2331	1909
Period (51)	4-5 pm	2116	2624	2822	2822	2709	2438	1997
Period (51)	5-6 pm	2138	2651	2850	2850	2736	2679	2195
Period (51)	6-7 pm	2138	2651	2850	2850	2736	2652	2173
Period (51)	7-8 pm	2052	2544	2736	2736	2627	2599	2129
Period (51)	8-9 pm	1945	2412	2594	2594	2490	2518	2063
Period (51)	9-10 pm	1774	2200	2366	2366	2271	2465	2019
Period (51)	10-11 pm	1560	1935	2081	2081	1997	2331	1909
Period (51)	11pm-12am	1347	1670	1796	1796	1724	2170	1778
Period (52)	12-1 am	1367	1695	1822	1822	1750	1994	1634
Period (52)	1-2 am	1285	1594	1714	1714	1645	1841	1508
Period (52)	2-3 am	1224	1518	1632	1632	1567	1739	1424
Period (52)	3-4 am	1204	1492	1605	1605	1541	1687	1382
Period (52)	4-5 am	1204	1492	1605	1605	1541	1636	1340
Period (52)	5-6 am	1224	1518	1632	1632	1567	1662	1361
Period (52)	6-7 am	1510	1872	2013	2013	1932	1687	1382
Period (52)	7-8 am	1754	2175	2339	2339	2246	1790	1466
Period (52)	8-9 am	1938	2403	2584	2584	2481	2045	1676
Period	9-10 am	1958	2428	2611	2611	2507	2250	1843

(52)								
Period (52)	10-11am	1958	2428	2611	2611	2507	2301	1885
Period (52)	11am-12 pm	1938	2403	2584	2584	2481	2327	1906
Period (52)	12-1 pm	1938	2403	2584	2584	2481	2301	1885
Period (52)	1-2 pm	1938	2403	2584	2584	2481	2250	1843
Period (52)	2-3 pm	1897	2353	2530	2530	2428	2224	1822
Period (52)	3-4 pm	1918	2378	2557	2557	2455	2224	1822
Period (52)	4-5 pm	2020	2504	2693	2693	2585	2327	1906
Period (52)	5-6 pm	2040	2530	2720	2720	2611	2557	2094
Period (52)	6-7 pm	2040	2530	2720	2720	2611	2531	2073
Period (52)	7-8 pm	1958	2428	2611	2611	2507	2480	2032
Period (52)	8-9 pm	1856	2302	2475	2475	2376	2403	1969
Period (52)	9-10 pm	1693	2100	2258	2258	2167	2352	1927
Period (52)	10-11 pm	1489	1847	1986	1986	1906	2224	1822
Period (52)	11pm-12am	1285	1594	1714	1714	1645	2071	1696

Dataset for the Load Demand with 36-unit system

Periods	Hours	Weeks						
		**Sunday (weekday)**	**Monday (weekday)**	**Tuesday (weekday)**	**Wensday (weekday)**	**Thursday (weekday)**	**Friday (weekend)**	**Saturday (weekend)**
Period (1)	12-1 am	2472	3066	3296	3296	3164	3608	2954
Period (1)	1-2 am	2324	2882	3100	3100	2976	3330	2728
Period (1)	2-3 am	2214	2746	2952	2952	2834	3144	2576
Period (1)	3-4 am	2178	2700	2902	2902	2786	3052	2500
Period (1)	4-5 am	2178	2700	2902	2902	2786	2960	2424
Period (1)	5-6 am	2214	2746	2952	2952	2834	3006	2462
Period (1)	6-7 am	2730	3386	3640	3640	3496	3052	2500
Period (1)	7-8 am	3174	3936	4232	4232	4062	3238	2652
Period (1)	8-9 am	3506	4346	4674	4674	4488	3700	3030
Period (1)	9-10 am	3542	4392	4724	4724	4534	4070	3334
Period (1)	10-11am	3542	4392	4724	4724	4534	4162	3410
Period (1)	11am-12 pm	3506	4346	4674	4674	4488	4208	3448
Period (1)	12-1 pm	3506	4346	4674	4674	4488	4162	3410
Period (1)	1-2 pm	3506	4346	4674	4674	4488	4070	3334
Period (1)	2-3 pm	3432	4256	4576	4576	4392	4024	3296
Period (1)	3-4 pm	3468	4302	4624	4624	4440	4024	3296
Period (1)	4-5 pm	3654	4530	4870	4870	4676	4208	3448
Period (1)	5-6 pm	3690	4576	4920	4920	4724	4624	3788
Period (1)	6-7 pm	3690	4576	4920	4920	4724	4578	3750
Period (1)	7-8 pm	3542	4392	4724	4724	4534	4486	3674
Period (1)	8-9 pm	3358	4164	4478	4478	4298	4348	3562
Period (1)	9-10 pm	3062	3798	4084	4084	3920	4254	3486
Period (1)	10-11 pm	2694	3340	3592	3592	3448	4024	3296
Period (1)	11pm-12am	2324	2882	3100	3100	2976	3746	3068
Period (2)	12-1 am	2582	3202	3444	3444	3306	3768	3088
Period (2)	1-2 am	2428	3012	3238	3238	3108	3478	2850
Period (2)	2-3 am	2314	2868	3084	3084	2960	3286	2692
Period (2)	3-4 am	2274	2820	3032	3032	2912	3188	2612
Period (2)	4-5 am	2274	2820	3032	3032	2912	3092	2532
Period (2)	5-6 am	2314	2868	3084	3084	2960	3140	2572
Period (2)	6-7 am	2852	3538	3804	3804	3652	3188	2612
Period (2)	7-8 am	3316	4110	4420	4420	4244	3382	2770

Period (2)	8-9 am	3662	4542	4884	4884	4688	3866	3166
Period (2)	9-10 am	3700	4588	4934	4934	4738	4252	3482
Period (2)	10-11am	3700	4588	4934	4934	4738	4348	3562
Period (2)	11am-12 pm	3662	4542	4884	4884	4688	4396	3602
Period (2)	12-1 pm	3662	4542	4884	4884	4688	4348	3562
Period (2)	1-2 pm	3662	4542	4884	4884	4688	4252	3482
Period (2)	2-3 pm	3586	4446	4780	4780	4588	4204	3444
Period (2)	3-4 pm	3624	4494	4832	4832	4638	4204	3444
Period (2)	4-5 pm	3816	4732	5088	5088	4886	4396	3602
Period (2)	5-6 pm	3856	4780	5140	5140	4934	4832	3958
Period (2)	6-7 pm	3856	4780	5140	5140	4934	4784	3918
Period (2)	7-8 pm	3700	4588	4934	4934	4738	4686	3840
Period (2)	8-9 pm	3508	4350	4678	4678	4490	4542	3720
Period (2)	9-10 pm	3200	3968	4266	4266	4096	4446	3642
Period (2)	10-11 pm	2814	3490	3752	3752	3602	4204	3444
Period (2)	11pm-12am	2428	3012	3238	3238	3108	3914	3206
Period (3)	12-1 am	2512	3116	3350	3350	3216	3666	3004
Period (3)	1-2 am	2362	2930	3150	3150	3024	3384	2772
Period (3)	2-3 am	2250	2790	3000	3000	2880	3196	2618
Period (3)	3-4 am	2212	2744	2950	2950	2832	3102	2542
Period (3)	4-5 am	2212	2744	2950	2950	2832	3008	2464
Period (3)	5-6 am	2250	2790	3000	3000	2880	3056	2502
Period (3)	6-7 am	2776	3442	3700	3700	3552	3102	2542
Period (3)	7-8 am	3226	4000	4300	4300	4128	3290	2696
Period (3)	8-9 am	3562	4418	4750	4750	4560	3760	3080
Period (3)	9-10 am	3600	4464	4800	4800	4608	4136	3388
Period (3)	10-11am	3600	4464	4800	4800	4608	4230	3466
Period (3)	11am-12 pm	3562	4418	4750	4750	4560	4278	3504
Period (3)	12-1 pm	3562	4418	4750	4750	4560	4230	3466
Period (3)	1-2 pm	3562	4418	4750	4750	4560	4136	3388
Period (3)	2-3 pm	3488	4324	4650	4650	4464	4090	3350
Period (3)	3-4 pm	3526	4372	4700	4700	4512	4090	3350
Period (3)	4-5 pm	3712	4604	4950	4950	4752	4278	3504
Period (3)	5-6 pm	3750	4650	5000	5000	4800	4700	3850
Period (3)	6-7 pm	3750	4650	5000	5000	4800	4654	3812
Period (3)	7-8 pm	3600	4464	4800	4800	4608	4560	3734
Period	8-9 pm	3412	4232	4550	4550	4368	4418	3620

(3)								
Period (3)	9-10 pm	3112	3860	4150	4150	3984	4324	3542
Period (3)	10-11 pm	2738	3394	3650	3650	3504	4090	3350
Period (4)	11pm-12am	2362	2930	3150	3150	3024	3808	3118
Period (4)	12-1 am	2392	2966	3190	3190	3062	3490	2858
Period (4)	1-2 am	2250	2788	2998	2998	2878	3222	2638
Period (4)	2-3 am	2142	2656	2856	2856	2742	3042	2492
Period (4)	3-4 am	2106	2612	2808	2808	2696	2954	2420
Period (4)	4-5 am	2106	2612	2808	2808	2696	2864	2346
Period (4)	5-6 am	2142	2656	2856	2856	2742	2908	2382
Period (4)	6-7 am	2642	3276	3522	3522	3382	2954	2420
Period (4)	7-8 am	3070	3808	4094	4094	3930	3132	2566
Period (4)	8-9 am	3392	4206	4522	4522	4342	3580	2932
Period (4)	9-10 am	3428	4250	4570	4570	4386	3938	3226
Period (4)	10-11am	3428	4250	4570	4570	4386	4026	3298
Period (4)	11am-12 pm	3392	4206	4522	4522	4342	4072	3336
Period (4)	12-1 pm	3392	4206	4522	4522	4342	4026	3298
Period (4)	1-2 pm	3392	4206	4522	4522	4342	3938	3226
Period (4)	2-3 pm	3320	4116	4426	4426	4250	3892	3188
Period (4)	3-4 pm	3356	4162	4474	4474	4296	3892	3188
Period (4)	4-5 pm	3534	4382	4712	4712	4524	4072	3336
Period (4)	5-6 pm	3570	4426	4760	4760	4570	4474	3666
Period (4)	6-7 pm	3570	4426	4760	4760	4570	4430	3628
Period (4)	7-8 pm	3428	4250	4570	4570	4386	4340	3556
Period (4)	8-9 pm	3248	4028	4332	4332	4158	4206	3446
Period (4)	9-10 pm	2964	3674	3950	3950	3792	4116	3372
Period (4)	10-11 pm	2606	3232	3474	3474	3336	3892	3188
Period (4)	11pm-12am	2250	2788	2998	2998	2878	3624	2968
Period (5)	12-1 am	2522	3128	3364	3364	3228	3680	3016
Period (5)	1-2 am	2372	2942	3162	3162	3036	3398	2784
Period (5)	2-3 am	2260	2802	3012	3012	2892	3208	2628
Period (5)	3-4 am	2222	2754	2962	2962	2844	3114	2552
Period (5)	4-5 am	2222	2754	2962	2962	2844	3020	2474
Period (5)	5-6 am	2260	2802	3012	3012	2892	3068	2512
Period (5)	6-7 am	2786	3454	3714	3714	3566	3114	2552
Period (5)	7-8 am	3238	4014	4318	4318	4144	3304	2706
Period (5)	8-9 am	3576	4436	4770	4770	4578	3776	3092

Period (5)	9-10 am	3614	4482	4820	4820	4626	4152	3402
Period (5)	10-11am	3614	4482	4820	4820	4626	4246	3478
Period (5)	11am-12 pm	3576	4436	4770	4770	4578	4294	3518
Period (5)	12-1 pm	3576	4436	4770	4770	4578	4246	3478
Period (5)	1-2 pm	3576	4436	4770	4770	4578	4152	3402
Period (5)	2-3 pm	3502	4342	4668	4668	4482	4106	3362
Period (5)	3-4 pm	3540	4388	4718	4718	4530	4106	3362
Period (5)	4-5 pm	3728	4622	4970	4970	4772	4294	3518
Period (5)	5-6 pm	3766	4668	5020	5020	4820	4718	3866
Period (5)	6-7 pm	3766	4668	5020	5020	4820	4672	3826
Period (5)	7-8 pm	3614	4482	4820	4820	4626	4578	3750
Period (5)	8-9 pm	3426	4248	4568	4568	4386	4436	3634
Period (5)	9-10 pm	3124	3874	4166	4166	4000	4342	3556
Period (5)	10-11 pm	2748	3408	3664	3664	3518	4106	3362
Period (5)	11pm-12am	2372	2942	3162	3162	3036	3822	3130
Period (6)	12-1 am	2412	2990	3216	3216	3088	3520	2882
Period (6)	1-2 am	2268	2812	3024	3024	2904	3248	2662
Period (6)	2-3 am	2160	2678	2880	2880	2764	3068	2514
Period (6)	3-4 am	2124	2634	2832	2832	2718	2978	2440
Period (6)	4-5 am	2124	2634	2832	2832	2718	2888	2366
Period (6)	5-6 am	2160	2678	2880	2880	2764	2932	2402
Period (6)	6-7 am	2664	3304	3552	3552	3410	2978	2440
Period (6)	7-8 am	3096	3840	4128	4128	3962	3158	2588
Period (6)	8-9 am	3420	4240	4560	4560	4378	3610	2956
Period (6)	9-10 am	3456	4286	4608	4608	4424	3970	3252
Period (6)	10-11am	3456	4286	4608	4608	4424	4060	3326
Period (6)	11am-12 pm	3420	4240	4560	4560	4378	4106	3364
Period (6)	12-1 pm	3420	4240	4560	4560	4378	4060	3326
Period (6)	1-2 pm	3420	4240	4560	4560	4378	3970	3252
Period (6)	2-3 pm	3348	4152	4464	4464	4286	3926	3216
Period (6)	3-4 pm	3384	4196	4512	4512	4332	3926	3216
Period (6)	4-5 pm	3564	4420	4752	4752	4562	4106	3364
Period (6)	5-6 pm	3600	4464	4800	4800	4608	4512	3696
Period (6)	6-7 pm	3600	4464	4800	4800	4608	4466	3660
Period (6)	7-8 pm	3456	4286	4608	4608	4424	4376	3586
Period (6)	8-9 pm	3276	4062	4368	4368	4194	4242	3474
Period	9-10 pm	2988	3706	3984	3984	3824	4152	3400

(6)								
Period (6)	10-11 pm	2628	3258	3504	3504	3364	3926	3216
Period (6)	11pm-12am	2268	2812	3024	3024	2904	3654	2994
Period (7)	12-1 am	2382	2954	3176	3176	3048	3476	2846
Period (7)	1-2 am	2240	2778	2986	2986	2866	3208	2628
Period (7)	2-3 am	2134	2644	2844	2844	2730	3030	2482
Period (7)	3-4 am	2098	2600	2796	2796	2684	2940	2408
Period (7)	4-5 am	2098	2600	2796	2796	2684	2852	2336
Period (7)	5-6 am	2134	2644	2844	2844	2730	2896	2372
Period (7)	6-7 am	2630	3262	3508	3508	3368	2940	2408
Period (7)	7-8 am	3058	3792	4076	4076	3914	3118	2554
Period (7)	8-9 am	3378	4188	4504	4504	4322	3564	2920
Period (7)	9-10 am	3412	4232	4550	4550	4368	3920	3212
Period (7)	10-11am	3412	4232	4550	4550	4368	4010	3284
Period (7)	11am-12 pm	3378	4188	4504	4504	4322	4054	3322
Period (7)	12-1 pm	3378	4188	4504	4504	4322	4010	3284
Period (7)	1-2 pm	3378	4188	4504	4504	4322	3920	3212
Period (7)	2-3 pm	3306	4100	4408	4408	4232	3876	3176
Period (7)	3-4 pm	3342	4144	4456	4456	4278	3876	3176
Period (7)	4-5 pm	3520	4364	4692	4692	4504	4054	3322
Period (7)	5-6 pm	3556	4408	4740	4740	4550	4456	3650
Period (7)	6-7 pm	3556	4408	4740	4740	4550	4412	3614
Period (7)	7-8 pm	3412	4232	4550	4550	4368	4322	3540
Period (7)	8-9 pm	3236	4012	4314	4314	4140	4188	3430
Period (7)	9-10 pm	2950	3658	3934	3934	3776	4100	3358
Period (7)	10-11 pm	2596	3218	3460	3460	3322	3876	3176
Period (7)	11pm-12am	2240	2778	2986	2986	2866	3610	2956
Period (8)	12-1 am	2312	2866	3082	3082	2958	3372	2762
Period (8)	1-2 am	2174	2696	2898	2898	2782	3114	2550
Period (8)	2-3 am	2070	2566	2760	2760	2650	2940	2408
Period (8)	3-4 am	2036	2524	2714	2714	2606	2854	2338
Period (8)	4-5 am	2036	2524	2714	2714	2606	2768	2266
Period (8)	5-6 am	2070	2566	2760	2760	2650	2810	2302
Period (8)	6-7 am	2554	3166	3404	3404	3268	2854	2338
Period (8)	7-8 am	2968	3680	3956	3956	3798	3026	2480
Period (8)	8-9 am	3278	4064	4370	4370	4196	3460	2834
Period (8)	9-10 am	3312	4106	4416	4416	4240	3806	3116

Period (8)	10-11am	3312	4106	4416	4416	4240	3892	3188
Period (8)	11am-12 pm	3278	4064	4370	4370	4196	3934	3224
Period (8)	12-1 pm	3278	4064	4370	4370	4196	3892	3188
Period (8)	1-2 pm	3278	4064	4370	4370	4196	3806	3116
Period (8)	2-3 pm	3208	3978	4278	4278	4106	3762	3082
Period (8)	3-4 pm	3244	4022	4324	4324	4152	3762	3082
Period (8)	4-5 pm	3416	4236	4554	4554	4372	3934	3224
Period (8)	5-6 pm	3450	4278	4600	4600	4416	4324	3542
Period (8)	6-7 pm	3450	4278	4600	4600	4416	4280	3506
Period (8)	7-8 pm	3312	4106	4416	4416	4240	4194	3436
Period (8)	8-9 pm	3140	3892	4186	4186	4018	4064	3330
Period (8)	9-10 pm	2864	3550	3818	3818	3666	3978	3258
Period (8)	10-11 pm	2518	3122	3358	3358	3224	3762	3082
Period (8)	11pm-12am	2174	2696	2898	2898	2782	3502	2870
Period (9)	12-1 am	1994	2472	2658	2658	2552	2976	2438
Period (9)	1-2 am	1962	2432	2616	2616	2512	2896	2372
Period (9)	2-3 am	1900	2354	2532	2532	2430	2738	2242
Period (9)	3-4 am	1836	2276	2448	2448	2350	2618	2144
Period (9)	4-5 am	1868	2316	2490	2490	2390	2578	2112
Period (9)	5-6 am	2058	2550	2744	2744	2634	2578	2112
Period (9)	6-7 am	2278	2826	3038	3038	2916	2698	2210
Period (9)	7-8 am	2690	3336	3588	3588	3444	2936	2404
Period (9)	8-9 am	3006	3728	4010	4010	3848	3292	2698
Period (9)	9-10 am	3134	3884	4178	4178	4010	3530	2892
Period (9)	10-11am	3166	3924	4220	4220	4052	3650	2990
Period (9)	11am-12 pm	3134	3884	4178	4178	4010	3728	3054
Period (9)	12-1 pm	2944	3650	3924	3924	3768	3610	2956
Period (9)	1-2 pm	2912	3610	3882	3882	3728	3570	2924
Period (9)	2-3 pm	2848	3532	3798	3798	3646	3570	2924
Period (9)	3-4 pm	2786	3454	3714	3714	3566	3412	2794
Period (9)	4-5 pm	2848	3532	3798	3798	3646	3372	2762
Period (9)	5-6 pm	2912	3610	3882	3882	3728	3490	2860
Period (9)	6-7 pm	3038	3768	4052	4052	3890	3650	2990
Period (9)	7-8 pm	3102	3846	4136	4136	3970	3966	3250
Period (9)	8-9 pm	3038	3768	4052	4052	3890	3848	3152
Period (9)	9-10 pm	2848	3532	3798	3798	3646	3768	3086
Period	10-11	2532	3140	3376	3376	3240	3570	2924

(9)	pm							
Period (9)	11pm-12am	2216	2746	2954	2954	2836	3372	2762
Period (10)	12-1 am	1984	2460	2646	2646	2540	2962	2426
Period (10)	1-2 am	1954	2422	2604	2604	2500	2882	2360
Period (10)	2-3 am	1890	2344	2520	2520	2420	2724	2232
Period (10)	3-4 am	1828	2266	2436	2436	2338	2606	2134
Period (10)	4-5 am	1858	2304	2478	2478	2378	2566	2102
Period (10)	5-6 am	2048	2538	2730	2730	2620	2566	2102
Period (10)	6-7 am	2268	2812	3024	3024	2904	2684	2200
Period (10)	7-8 am	2678	3320	3570	3570	3428	2922	2394
Period (10)	8-9 am	2992	3710	3990	3990	3830	3276	2684
Period (10)	9-10 am	3118	3866	4158	4158	3992	3514	2878
Period (10)	10-11am	3150	3906	4200	4200	4032	3632	2976
Period (10)	11am-12 pm	3118	3866	4158	4158	3992	3712	3040
Period (10)	12-1 pm	2930	3632	3906	3906	3750	3592	2942
Period (10)	1-2 pm	2898	3594	3864	3864	3710	3554	2910
Period (10)	2-3 pm	2836	3516	3780	3780	3628	3554	2910
Period (10)	3-4 pm	2772	3438	3696	3696	3548	3396	2782
Period (10)	4-5 pm	2836	3516	3780	3780	3628	3356	2748
Period (10)	5-6 pm	2898	3594	3864	3864	3710	3474	2846
Period (10)	6-7 pm	3024	3750	4032	4032	3870	3632	2976
Period (10)	7-8 pm	3088	3828	4116	4116	3952	3948	3234
Period (10)	8-9 pm	3024	3750	4032	4032	3870	3830	3136
Period (10)	9-10 pm	2836	3516	3780	3780	3628	3750	3072
Period (10)	10-11 pm	2520	3124	3360	3360	3226	3554	2910
Period (10)	11pm-12am	2206	2734	2940	2940	2822	3356	2748
Period (11)	12-1 am	1928	2390	2570	2570	2468	2876	2356
Period (11)	1-2 am	1898	2352	2530	2530	2428	2800	2294
Period (11)	2-3 am	1836	2276	2448	2448	2350	2646	2168
Period (11)	3-4 am	1774	2200	2366	2366	2272	2532	2074
Period (11)	4-5 am	1806	2238	2408	2408	2310	2492	2042
Period (11)	5-6 am	1990	2466	2652	2652	2546	2492	2042
Period (11)	6-7 am	2204	2732	2938	2938	2820	2608	2136
Period (11)	7-8 am	2602	3224	3468	3468	3330	2838	2324
Period (11)	8-9 am	2908	3604	3876	3876	3720	3184	2608
Period (11)	9-10 am	3030	3756	4040	4040	3878	3414	2796
Period (11)	10-11am	3060	3794	4080	4080	3916	3528	2890

Period (11)	11am-12 pm	3030	3756	4040	4040	3878	3606	2954
Period (11)	12-1 pm	2846	3528	3794	3794	3642	3490	2858
Period (11)	1-2 pm	2816	3490	3754	3754	3604	3452	2828
Period (11)	2-3 pm	2754	3414	3672	3672	3526	3452	2828
Period (11)	3-4 pm	2692	3338	3590	3590	3446	3298	2702
Period (11)	4-5 pm	2754	3414	3672	3672	3526	3260	2670
Period (11)	5-6 pm	2816	3490	3754	3754	3604	3374	2764
Period (11)	6-7 pm	2938	3642	3916	3916	3760	3528	2890
Period (11)	7-8 pm	2998	3718	3998	3998	3838	3836	3142
Period (11)	8-9 pm	2938	3642	3916	3916	3760	3720	3048
Period (11)	9-10 pm	2754	3414	3672	3672	3526	3644	2984
Period (11)	10-11 pm	2448	3036	3264	3264	3134	3452	2828
Period (11)	11pm-12am	2142	2656	2856	2856	2742	3260	2670
Period (12)	12-1 am	1956	2426	2608	2608	2504	2918	2390
Period (12)	1-2 am	1926	2388	2566	2566	2464	2840	2328
Period (12)	2-3 am	1864	2310	2484	2484	2384	2686	2200
Period (12)	3-4 am	1800	2234	2402	2402	2306	2568	2104
Period (12)	4-5 am	1832	2272	2442	2442	2344	2530	2072
Period (12)	5-6 am	2018	2502	2692	2692	2584	2530	2072
Period (12)	6-7 am	2236	2772	2980	2980	2862	2646	2168
Period (12)	7-8 am	2640	3272	3520	3520	3378	2880	2358
Period (12)	8-9 am	2950	3658	3934	3934	3776	3230	2646
Period (12)	9-10 am	3074	3812	4098	4098	3934	3464	2838
Period (12)	10-11am	3106	3850	4140	4140	3974	3580	2932
Period (12)	11am-12 pm	3074	3812	4098	4098	3934	3658	2996
Period (12)	12-1 pm	2888	3580	3850	3850	3696	3542	2900
Period (12)	1-2 pm	2856	3542	3808	3808	3656	3502	2870
Period (12)	2-3 pm	2794	3466	3726	3726	3576	3502	2870
Period (12)	3-4 pm	2732	3388	3644	3644	3498	3346	2742
Period (12)	4-5 pm	2794	3466	3726	3726	3576	3308	2710
Period (12)	5-6 pm	2856	3542	3808	3808	3656	3424	2806
Period (12)	6-7 pm	2980	3696	3974	3974	3816	3580	2932
Period (12)	7-8 pm	3042	3774	4058	4058	3894	3892	3188
Period (12)	8-9 pm	2980	3696	3974	3974	3816	3774	3092
Period (12)	9-10 pm	2794	3466	3726	3726	3576	3698	3028
Period (12)	10-11 pm	2484	3080	3312	3312	3180	3502	2870
Period	11pm-	2174	2696	2898	2898	2782	3308	2710

(12)	12am							
Period (13)	12-1 am	1900	1528	2532	2532	2432	2834	2322
Period (13)	1-2 am	1870	1480	2492	2492	2392	2758	2260
Period (13)	2-3 am	1810	1386	2412	2412	2316	2608	2136
Period (13)	3-4 am	1748	1296	2332	2332	2238	2494	2042
Period (13)	4-5 am	1778	1340	2372	2372	2276	2456	2012
Period (13)	5-6 am	1960	1626	2614	2614	2508	2456	2012
Period (13)	6-7 am	2170	1996	2894	2894	2778	2570	2104
Period (13)	7-8 am	2562	2782	3418	3418	3280	2796	2290
Period (13)	8-9 am	2864	3474	3820	3820	3666	3136	2570
Period (13)	9-10 am	2984	3774	3980	3980	3820	3364	2754
Period (13)	10-11am	3016	3850	4020	4020	3860	3476	2848
Period (13)	11am-12 pm	2984	3774	3980	3980	3820	3552	2910
Period (13)	12-1 pm	2804	3330	3738	3738	3590	3438	2816
Period (13)	1-2 pm	2774	3258	3698	3698	3550	3400	2786
Period (13)	2-3 pm	2714	3118	3618	3618	3474	3400	2786
Period (13)	3-4 pm	2654	2982	3538	3538	3396	3250	2662
Period (13)	4-5 pm	2714	3118	3618	3618	3474	3212	2632
Period (13)	5-6 pm	2774	3258	3698	3698	3550	3326	2724
Period (13)	6-7 pm	2894	3548	3860	3860	3704	3476	2848
Period (13)	7-8 pm	2954	3698	3940	3940	3782	3778	3096
Period (13)	8-9 pm	2894	3548	3860	3860	3704	3666	3002
Period (13)	9-10 pm	2714	3118	3618	3618	3474	3590	2940
Period (13)	10-11 pm	2412	2464	3216	3216	3088	3400	2786
Period (13)	11pm-12am	2110	1886	2814	2814	2702	3212	2632
Period (14)	12-1 am	2022	2508	2696	2696	2588	3018	2472
Period (14)	1-2 am	1990	2468	2654	2654	2548	2936	2406
Period (14)	2-3 am	1926	2388	2568	2568	2466	2776	2274
Period (14)	3-4 am	1862	2308	2482	2482	2384	2656	2176
Period (14)	4-5 am	1894	2348	2526	2526	2424	2616	2142
Period (14)	5-6 am	2086	2588	2782	2782	2670	2616	2142
Period (14)	6-7 am	2312	2866	3082	3082	2958	2736	2242
Period (14)	7-8 am	2728	3384	3638	3638	3492	2978	2438
Period (14)	8-9 am	3050	3782	4066	4066	3904	3340	2736
Period (14)	9-10 am	3178	3940	4238	4238	4068	3580	2934
Period (14)	10-11am	3210	3980	4280	4280	4108	3702	3032
Period (14)	11am-12 pm	3178	3940	4238	4238	4068	3782	3098

Period (14)	12-1 pm	2986	3702	3980	3980	3822	3662	2998
Period (14)	1-2 pm	2954	3662	3938	3938	3780	3620	2966
Period (14)	2-3 pm	2890	3582	3852	3852	3698	3620	2966
Period (14)	3-4 pm	2824	3502	3766	3766	3616	3460	2834
Period (14)	4-5 pm	2890	3582	3852	3852	3698	3420	2802
Period (14)	5-6 pm	2954	3662	3938	3938	3780	3540	2900
Period (14)	6-7 pm	3082	3820	4108	4108	3944	3702	3032
Period (14)	7-8 pm	3146	3900	4194	4194	4026	4024	3296
Period (14)	8-9 pm	3082	3820	4108	4108	3944	3902	3196
Period (14)	9-10 pm	2890	3582	3852	3852	3698	3822	3130
Period (14)	10-11 pm	2568	3184	3424	3424	3288	3620	2966
Period (14)	11pm-12am	2248	2786	2996	2996	2876	3420	2802
Period (15)	12-1 am	1938	2402	2584	2584	2480	2890	2368
Period (15)	1-2 am	1906	2364	2542	2542	2440	2814	2304
Period (15)	2-3 am	1846	2288	2460	2460	2362	2660	2178
Period (15)	3-4 am	1784	2212	2378	2378	2282	2544	2084
Period (15)	4-5 am	1814	2250	2420	2420	2322	2506	2052
Period (15)	5-6 am	1998	2480	2666	2666	2558	2506	2052
Period (15)	6-7 am	2214	2746	2952	2952	2834	2620	2146
Period (15)	7-8 am	2614	3242	3486	3486	3346	2852	2336
Period (15)	8-9 am	2922	3624	3896	3896	3740	3198	2620
Period (15)	9-10 am	3044	3776	4060	4060	3896	3430	2810
Period (15)	10-11am	3076	3814	4100	4100	3936	3546	2904
Period (15)	11am-12 pm	3044	3776	4060	4060	3896	3622	2968
Period (15)	12-1 pm	2860	3548	3814	3814	3660	3508	2872
Period (15)	1-2 pm	2830	3508	3772	3772	3622	3468	2842
Period (15)	2-3 pm	2768	3432	3690	3690	3542	3468	2842
Period (15)	3-4 pm	2706	3356	3608	3608	3464	3314	2716
Period (15)	4-5 pm	2768	3432	3690	3690	3542	3276	2684
Period (15)	5-6 pm	2830	3508	3772	3772	3622	3392	2778
Period (15)	6-7 pm	2952	3662	3936	3936	3778	3546	2904
Period (15)	7-8 pm	3014	3738	4018	4018	3858	3854	3158
Period (15)	8-9 pm	2952	3662	3936	3936	3778	3738	3062
Period (15)	9-10 pm	2768	3432	3690	3690	3542	3662	3000
Period (15)	10-11 pm	2460	3052	3280	3280	3148	3468	2842
Period (15)	11pm-12am	2152	2670	2870	2870	2756	3276	2684
Period	12-1 am	2154	2672	2872	2872	2758	3214	2634

(16)								
Period (16)	1-2 am	2120	2628	2828	2828	2714	3130	2564
Period (16)	2-3 am	2052	2544	2736	2736	2626	2958	2422
Period (16)	3-4 am	1984	2460	2644	2644	2540	2830	2318
Period (16)	4-5 am	2018	2502	2690	2690	2582	2786	2282
Period (16)	5-6 am	2224	2756	2964	2964	2846	2786	2282
Period (16)	6-7 am	2462	3052	3284	3284	3152	2914	2388
Period (16)	7-8 am	2908	3604	3876	3876	3720	3172	2598
Period (16)	8-9 am	3250	4028	4332	4332	4158	3558	2914
Period (16)	9-10 am	3386	4198	4514	4514	4334	3814	3124
Period (16)	10-11am	3420	4240	4560	4560	4378	3944	3230
Period (16)	11am-12 pm	3386	4198	4514	4514	4334	4030	3300
Period (16)	12-1 pm	3180	3944	4240	4240	4072	3900	3196
Period (16)	1-2 pm	3146	3900	4196	4196	4028	3858	3160
Period (16)	2-3 pm	3078	3816	4104	4104	3940	3858	3160
Period (16)	3-4 pm	3010	3732	4012	4012	3852	3686	3020
Period (16)	4-5 pm	3078	3816	4104	4104	3940	3644	2984
Period (16)	5-6 pm	3146	3900	4196	4196	4028	3772	3090
Period (16)	6-7 pm	3284	4070	4378	4378	4202	3944	3230
Period (16)	7-8 pm	3352	4156	4468	4468	4290	4286	3512
Period (16)	8-9 pm	3284	4070	4378	4378	4202	4158	3406
Period (16)	9-10 pm	3078	3816	4104	4104	3940	4072	3336
Period (16)	10-11 pm	2736	3392	3648	3648	3502	3858	3160
Period (16)	11pm-12am	2394	2968	3192	3192	3064	3644	2984
Period (17)	12-1 am	2032	2520	2710	2710	2600	3032	2484
Period (17)	1-2 am	2000	2480	2666	2666	2560	2950	2418
Period (17)	2-3 am	1936	2400	2580	2580	2476	2788	2284
Period (17)	3-4 am	1870	2320	2494	2494	2394	2668	2186
Period (17)	4-5 am	1902	2360	2538	2538	2436	2628	2152
Period (17)	5-6 am	2096	2600	2796	2796	2684	2628	2152
Period (17)	6-7 am	2322	2880	3096	3096	2972	2748	2252
Period (17)	7-8 am	2742	3400	3656	3656	3508	2992	2450
Period (17)	8-9 am	3064	3800	4086	4086	3922	3354	2748
Period (17)	9-10 am	3192	3960	4258	4258	4086	3598	2946
Period (17)	10-11am	3226	4000	4300	4300	4128	3718	3046
Period (17)	11am-12 pm	3192	3960	4258	4258	4086	3800	3112
Period (17)	12-1 pm	3000	3720	4000	4000	3840	3678	3014

Period (17)	1-2 pm	2968	3680	3956	3956	3798	3638	2980
Period (17)	2-3 pm	2902	3600	3870	3870	3716	3638	2980
Period (17)	3-4 pm	2838	3520	3784	3784	3632	3476	2848
Period (17)	4-5 pm	2902	3600	3870	3870	3716	3436	2814
Period (17)	5-6 pm	2968	3680	3956	3956	3798	3556	2914
Period (17)	6-7 pm	3096	3840	4128	4128	3962	3718	3046
Period (17)	7-8 pm	3160	3920	4214	4214	4046	4042	3312
Period (17)	8-9 pm	3096	3840	4128	4128	3962	3920	3212
Period (17)	9-10 pm	2902	3600	3870	3870	3716	3840	3146
Period (17)	10-11 pm	2580	3200	3440	3440	3302	3638	2980
Period (17)	11pm-12am	2258	2800	3010	3010	2890	3436	2814
Period (18)	12-1 am	2294	2846	3060	3060	2936	3324	2724
Period (18)	1-2 am	2152	2668	2868	2868	2754	3146	2576
Period (18)	2-3 am	2080	2578	2772	2772	2662	2966	2430
Period (18)	3-4 am	2008	2490	2676	2676	2570	2920	2392
Period (18)	4-5 am	2008	2490	2676	2676	2570	2876	2356
Period (18)	5-6 am	2080	2578	2772	2772	2662	2786	2282
Period (18)	6-7 am	2294	2846	3060	3060	2936	2786	2282
Period (18)	7-8 am	2724	3378	3632	3632	3488	2966	2430
Period (18)	8-9 am	3118	3868	4158	4158	3992	3640	2982
Period (18)	9-10 am	3406	4224	4542	4542	4360	3864	3166
Period (18)	10-11am	3550	4400	4732	4732	4542	4088	3350
Period (18)	11am-12 pm	3586	4446	4780	4780	4588	4178	3422
Period (18)	12-1 pm	3550	4400	4732	4732	4542	4178	3422
Period (18)	1-2 pm	3586	4446	4780	4780	4588	4134	3386
Period (18)	2-3 pm	3586	4446	4780	4780	4588	4088	3350
Period (18)	3-4 pm	3478	4312	4636	4636	4452	4088	3350
Period (18)	4-5 pm	3442	4268	4588	4588	4406	4134	3386
Period (18)	5-6 pm	3442	4268	4588	4588	4406	4224	3460
Period (18)	6-7 pm	3334	4134	4446	4446	4268	4268	3496
Period (18)	7-8 pm	3298	4090	4398	4398	4222	4268	3496
Period (18)	8-9 pm	3298	4090	4398	4398	4222	4494	3680
Period (18)	9-10 pm	3334	4134	4446	4446	4268	4178	3422
Period (18)	10-11 pm	3118	3868	4158	4158	3992	3954	3238
Period (18)	11pm-12am	2582	3200	3442	3442	3304	3594	2944
Period (19)	12-1 am	2380	2952	3174	3174	3048	3450	2826
Period	1-2 am	2232	2768	2976	2976	2856	3264	2674

(19)								
Period (19)	2-3 am	2158	2676	2876	2876	2762	3078	2520
Period (19)	3-4 am	2084	2584	2778	2778	2666	3030	2482
Period (19)	4-5 am	2084	2584	2778	2778	2666	2984	2444
Period (19)	5-6 am	2158	2676	2876	2876	2762	2890	2368
Period (19)	6-7 am	2380	2952	3174	3174	3048	2890	2368
Period (19)	7-8 am	2828	3506	3770	3770	3618	3078	2520
Period (19)	8-9 am	3236	4014	4316	4316	4142	3776	3094
Period (19)	9-10 am	3534	4382	4712	4712	4524	4010	3284
Period (19)	10-11am	3682	4566	4910	4910	4714	4242	3476
Period (19)	11am-12 pm	3720	4612	4960	4960	4762	4336	3552
Period (19)	12-1 pm	3682	4566	4910	4910	4714	4336	3552
Period (19)	1-2 pm	3720	4612	4960	4960	4762	4290	3514
Period (19)	2-3 pm	3720	4612	4960	4960	4762	4242	3476
Period (19)	3-4 pm	3608	4474	4812	4812	4618	4242	3476
Period (19)	4-5 pm	3572	4428	4762	4762	4572	4290	3514
Period (19)	5-6 pm	3572	4428	4762	4762	4572	4382	3590
Period (19)	6-7 pm	3460	4290	4612	4612	4428	4430	3628
Period (19)	7-8 pm	3422	4244	4564	4564	4380	4430	3628
Period (19)	8-9 pm	3422	4244	4564	4564	4380	4662	3820
Period (19)	9-10 pm	3460	4290	4612	4612	4428	4336	3552
Period (19)	10-11 pm	3236	4014	4316	4316	4142	4102	3360
Period (19)	11pm-12am	2678	3322	3572	3572	3428	3730	3056
Period (20)	12-1 am	2410	2988	3212	3212	3084	3492	2860
Period (20)	1-2 am	2260	2802	3012	3012	2892	3304	2706
Period (20)	2-3 am	2184	2708	2912	2912	2796	3114	2552
Period (20)	3-4 am	2108	2614	2812	2812	2698	3068	2512
Period (20)	4-5 am	2108	2614	2812	2812	2698	3020	2474
Period (20)	5-6 am	2184	2708	2912	2912	2796	2926	2396
Period (20)	6-7 am	2410	2988	3212	3212	3084	2926	2396
Period (20)	7-8 am	2862	3548	3816	3816	3662	3114	2552
Period (20)	8-9 am	3276	4062	4368	4368	4192	3822	3130
Period (20)	9-10 am	3576	4436	4770	4770	4578	4058	3324
Period (20)	10-11am	3728	4622	4970	4970	4772	4294	3518
Period (20)	11am-12 pm	3766	4668	5020	5020	4820	4388	3594
Period (20)	12-1 pm	3728	4622	4970	4970	4772	4388	3594
Period (20)	1-2 pm	3766	4668	5020	5020	4820	4342	3556

Period (20)	2-3 pm	3766	4668	5020	5020	4820	4294	3518
Period (20)	3-4 pm	3652	4528	4870	4870	4674	4294	3518
Period (20)	4-5 pm	3614	4482	4820	4820	4626	4342	3556
Period (20)	5-6 pm	3614	4482	4820	4820	4626	4436	3634
Period (20)	6-7 pm	3502	4342	4668	4668	4482	4482	3672
Period (20)	7-8 pm	3464	4296	4618	4618	4434	4482	3672
Period (20)	8-9 pm	3464	4296	4618	4618	4434	4718	3866
Period (20)	9-10 pm	3502	4342	4668	4668	4482	4388	3594
Period (20)	10-11 pm	3276	4062	4368	4368	4192	4152	3402
Period (20)	11pm-12am	2710	3362	3614	3614	3470	3776	3092
Period (21)	12-1 am	2342	2904	3124	3124	2998	3394	2780
Period (21)	1-2 am	2196	2724	2928	2928	2810	3212	2630
Period (21)	2-3 am	2122	2632	2830	2830	2718	3028	2480
Period (21)	3-4 am	2050	2542	2732	2732	2624	2982	2442
Period (21)	4-5 am	2050	2542	2732	2732	2624	2936	2404
Period (21)	5-6 am	2122	2632	2830	2830	2718	2844	2330
Period (21)	6-7 am	2342	2904	3124	3124	2998	2844	2330
Period (21)	7-8 am	2782	3450	3708	3708	3560	3028	2480
Period (21)	8-9 am	3184	3948	4246	4246	4076	3716	3044
Period (21)	9-10 am	3478	4312	4636	4636	4450	3944	3232
Period (21)	10-11am	3624	4494	4832	4832	4638	4174	3420
Period (21)	11am-12 pm	3660	4538	4880	4880	4684	4266	3494
Period (21)	12-1 pm	3624	4494	4832	4832	4638	4266	3494
Period (21)	1-2 pm	3660	4538	4880	4880	4684	4220	3456
Period (21)	2-3 pm	3660	4538	4880	4880	4684	4174	3420
Period (21)	3-4 pm	3550	4402	4734	4734	4544	4174	3420
Period (21)	4-5 pm	3514	4356	4684	4684	4498	4220	3456
Period (21)	5-6 pm	3514	4356	4684	4684	4498	4312	3532
Period (21)	6-7 pm	3404	4220	4538	4538	4356	4358	3570
Period (21)	7-8 pm	3368	4176	4490	4490	4310	4358	3570
Period (21)	8-9 pm	3368	4176	4490	4490	4310	4588	3758
Period (21)	9-10 pm	3404	4220	4538	4538	4356	4266	3494
Period (21)	10-11 pm	3184	3948	4246	4246	4076	4036	3306
Period (21)	11pm-12am	2636	3268	3514	3514	3374	3670	3006
Period (22)	12-1 am	2218	1858	2956	2956	2838	3214	2632
Period (22)	1-2 am	2080	1634	2772	2772	2662	3040	2490
Period	2-3 am	2010	1526	2680	2680	2572	2866	2348

(22)								
Period (22)	3-4 am	1940	1424	2588	2588	2484	2822	2312
Period (22)	4-5 am	1940	1424	2588	2588	2484	2780	2276
Period (22)	5-6 am	2010	1526	2680	2680	2572	2692	2206
Period (22)	6-7 am	2218	1858	2956	2956	2838	2692	2206
Period (22)	7-8 am	2634	2622	3512	3512	3370	2866	2348
Period (22)	8-9 am	3014	3436	4020	4020	3858	3518	2882
Period (22)	9-10 am	3292	4096	4390	4390	4214	3734	3060
Period (22)	10-11am	3430	4448	4574	4574	4390	3952	3238
Period (22)	11am-12 pm	3466	4538	4620	4620	4436	4038	3308
Period (22)	12-1 pm	3430	4448	4574	4574	4390	4038	3308
Period (22)	1-2 pm	3466	4538	4620	4620	4436	3996	3272
Period (22)	2-3 pm	3466	4538	4620	4620	4436	3952	3238
Period (22)	3-4 pm	3362	4270	4482	4482	4302	3952	3238
Period (22)	4-5 pm	3326	4182	4436	4436	4258	3996	3272
Period (22)	5-6 pm	3326	4182	4436	4436	4258	4082	3344
Period (22)	6-7 pm	3222	3926	4296	4296	4124	4126	3380
Period (22)	7-8 pm	3188	3842	4250	4250	4080	4126	3380
Period (22)	8-9 pm	3188	3842	4250	4250	4080	4342	3558
Period (22)	9-10 pm	3222	3926	4296	4296	4124	4038	3308
Period (22)	10-11 pm	3014	3436	4020	4020	3858	3822	3130
Period (22)	11pm-12am	2494	2352	3326	3326	3194	3474	2846
Period (23)	12-1 am	2468	3060	3290	3290	3158	3576	2928
Period (23)	1-2 am	2314	2868	3084	3084	2960	3382	2770
Period (23)	2-3 am	2236	2772	2982	2982	2862	3188	2612
Period (23)	3-4 am	2158	2676	2878	2878	2764	3140	2572
Period (23)	4-5 am	2158	2676	2878	2878	2764	3092	2532
Period (23)	5-6 am	2236	2772	2982	2982	2862	2996	2454
Period (23)	6-7 am	2468	3060	3290	3290	3158	2996	2454
Period (23)	7-8 am	2930	3632	3906	3906	3750	3188	2612
Period (23)	8-9 am	3354	4158	4472	4472	4292	3914	3206
Period (23)	9-10 am	3662	4542	4884	4884	4688	4156	3404
Period (23)	10-11am	3816	4732	5088	5088	4886	4396	3602
Period (23)	11am-12 pm	3856	4780	5140	5140	4934	4494	3680
Period (23)	12-1 pm	3816	4732	5088	5088	4886	4494	3680
Period (23)	1-2 pm	3856	4780	5140	5140	4934	4446	3642
Period (23)	2-3 pm	3856	4780	5140	5140	4934	4396	3602

Period (23)	3-4 pm	3740	4636	4986	4986	4786	4396	3602
Period (23)	4-5 pm	3700	4588	4934	4934	4738	4446	3642
Period (23)	5-6 pm	3700	4588	4934	4934	4738	4542	3720
Period (23)	6-7 pm	3586	4446	4780	4780	4588	4590	3760
Period (23)	7-8 pm	3546	4398	4728	4728	4540	4590	3760
Period (23)	8-9 pm	3546	4398	4728	4728	4540	4832	3958
Period (23)	9-10 pm	3586	4446	4780	4780	4588	4494	3680
Period (23)	10-11 pm	3354	4158	4472	4472	4292	4252	3482
Period (23)	11pm-12am	2776	3442	3700	3700	3552	3866	3166
Period (24)	12-1 am	2428	3012	3238	3238	3108	3520	2884
Period (24)	1-2 am	2278	2824	3036	3036	2914	3330	2728
Period (24)	2-3 am	2202	2730	2934	2934	2818	3140	2572
Period (24)	3-4 am	2126	2636	2834	2834	2720	3092	2532
Period (24)	4-5 am	2126	2636	2834	2834	2720	3044	2494
Period (24)	5-6 am	2202	2730	2934	2934	2818	2948	2416
Period (24)	6-7 am	2428	3012	3238	3238	3108	2948	2416
Period (24)	7-8 am	2884	3576	3846	3846	3692	3140	2572
Period (24)	8-9 am	3302	4094	4402	4402	4226	3852	3156
Period (24)	9-10 am	3606	4470	4808	4808	4614	4090	3350
Period (24)	10-11am	3758	4658	5010	5010	4810	4328	3546
Period (24)	11am-12 pm	3796	4706	5060	5060	4858	4424	3624
Period (24)	12-1 pm	3758	4658	5010	5010	4810	4424	3624
Period (24)	1-2 pm	3796	4706	5060	5060	4858	4376	3584
Period (24)	2-3 pm	3796	4706	5060	5060	4858	4328	3546
Period (24)	3-4 pm	3682	4564	4908	4908	4712	4328	3546
Period (24)	4-5 pm	3644	4518	4858	4858	4664	4376	3584
Period (24)	5-6 pm	3644	4518	4858	4858	4664	4472	3662
Period (24)	6-7 pm	3530	4376	4706	4706	4518	4518	3702
Period (24)	7-8 pm	3492	4330	4656	4656	4468	4518	3702
Period (24)	8-9 pm	3492	4330	4656	4656	4468	4756	3896
Period (24)	9-10 pm	3530	4376	4706	4706	4518	4424	3624
Period (24)	10-11 pm	3302	4094	4402	4402	4226	4186	3428
Period (24)	11pm-12am	2732	3388	3644	3644	3498	3806	3116
Period (25)	12-1 am	2448	3036	3264	3264	3134	3548	2906
Period (25)	1-2 am	2296	2846	3060	3060	2938	3356	2748
Period (25)	2-3 am	2218	2750	2958	2958	2840	3164	2592
Period	3-4 am	2142	2656	2856	2856	2742	3116	2552

(25)								
Period (25)	4-5 am	2142	2656	2856	2856	2742	3068	2514
Period (25)	5-6 am	2218	2750	2958	2958	2840	2972	2434
Period (25)	6-7 am	2448	3036	3264	3264	3134	2972	2434
Period (25)	7-8 am	2908	3604	3876	3876	3720	3164	2592
Period (25)	8-9 am	3328	4126	4438	4438	4260	3884	3180
Period (25)	9-10 am	3634	4506	4846	4846	4652	4122	3378
Period (25)	10-11am	3786	4696	5050	5050	4848	4362	3574
Period (25)	11am-12 pm	3826	4744	5100	5100	4896	4458	3652
Period (25)	12-1 pm	3786	4696	5050	5050	4848	4458	3652
Period (25)	1-2 pm	3826	4744	5100	5100	4896	4410	3612
Period (25)	2-3 pm	3826	4744	5100	5100	4896	4362	3574
Period (25)	3-4 pm	3710	4600	4948	4948	4750	4362	3574
Period (25)	4-5 pm	3672	4554	4896	4896	4700	4410	3612
Period (25)	5-6 pm	3672	4554	4896	4896	4700	4506	3692
Period (25)	6-7 pm	3558	4410	4744	4744	4554	4554	3730
Period (25)	7-8 pm	3520	4364	4692	4692	4504	4554	3730
Period (25)	8-9 pm	3520	4364	4692	4692	4504	4794	3928
Period (25)	9-10 pm	3558	4410	4744	4744	4554	4458	3652
Period (25)	10-11 pm	3328	4126	4438	4438	4260	4218	3456
Period (25)	11pm-12am	2754	3414	3672	3672	3526	3836	3142
Period (26)	12-1 am	2352	2916	3136	3136	3010	3408	2792
Period (26)	1-2 am	2206	2734	2940	2940	2822	3224	2642
Period (26)	2-3 am	2132	2644	2842	2842	2728	3040	2490
Period (26)	3-4 am	2058	2552	2744	2744	2634	2994	2452
Period (26)	4-5 am	2058	2552	2744	2744	2634	2948	2414
Period (26)	5-6 am	2132	2644	2842	2842	2728	2856	2340
Period (26)	6-7 am	2352	2916	3136	3136	3010	2856	2340
Period (26)	7-8 am	2794	3464	3724	3724	3576	3040	2490
Period (26)	8-9 am	3198	3964	4264	4264	4092	3730	3056
Period (26)	9-10 am	3492	4330	4656	4656	4468	3962	3244
Period (26)	10-11am	3638	4512	4852	4852	4656	4192	3434
Period (26)	11am-12 pm	3676	4558	4900	4900	4704	4284	3508
Period (26)	12-1 pm	3638	4512	4852	4852	4656	4284	3508
Period (26)	1-2 pm	3676	4558	4900	4900	4704	4238	3472
Period (26)	2-3 pm	3676	4558	4900	4900	4704	4192	3434
Period (26)	3-4 pm	3564	4420	4754	4754	4562	4192	3434

Period (26)	4-5 pm	3528	4374	4704	4704	4516	4238	3472
Period (26)	5-6 pm	3528	4374	4704	4704	4516	4330	3546
Period (26)	6-7 pm	3418	4238	4558	4558	4374	4376	3584
Period (26)	7-8 pm	3382	4192	4508	4508	4328	4376	3584
Period (26)	8-9 pm	3382	4192	4508	4508	4328	4606	3774
Period (26)	9-10 pm	3418	4238	4558	4558	4374	4284	3508
Period (26)	10-11 pm	3198	3964	4264	4264	4092	4054	3320
Period (26)	11pm-12am	2646	3282	3528	3528	3386	3684	3018
Period (27)	12-1 am	2064	2560	2752	2752	2642	2992	2450
Period (27)	1-2 am	1936	2400	2580	2580	2476	2830	2318
Period (27)	2-3 am	1870	2320	2494	2494	2394	2668	2186
Period (27)	3-4 am	1806	2240	2408	2408	2312	2628	2152
Period (27)	4-5 am	1806	2240	2408	2408	2312	2586	2120
Period (27)	5-6 am	1870	2320	2494	2494	2394	2506	2052
Period (27)	6-7 am	2064	2560	2752	2752	2642	2506	2052
Period (27)	7-8 am	2452	3040	3268	3268	3138	2668	2186
Period (27)	8-9 am	2806	3480	3742	3742	3592	3274	2682
Period (27)	9-10 am	3064	3800	4086	4086	3922	3476	2848
Period (27)	10-11am	3192	3960	4258	4258	4086	3678	3014
Period (27)	11am-12 pm	3226	4000	4300	4300	4128	3760	3080
Period (27)	12-1 pm	3192	3960	4258	4258	4086	3760	3080
Period (27)	1-2 pm	3226	4000	4300	4300	4128	3718	3046
Period (27)	2-3 pm	3226	4000	4300	4300	4128	3678	3014
Period (27)	3-4 pm	3128	3880	4172	4172	4004	3678	3014
Period (27)	4-5 pm	3096	3840	4128	4128	3962	3718	3046
Period (27)	5-6 pm	3096	3840	4128	4128	3962	3800	3112
Period (27)	6-7 pm	3000	3720	4000	4000	3840	3840	3146
Period (27)	7-8 pm	2968	3680	3956	3956	3798	3840	3146
Period (27)	8-9 pm	2968	3680	3956	3956	3798	4042	3312
Period (27)	9-10 pm	3000	3720	4000	4000	3840	3760	3080
Period (27)	10-11 pm	2806	3480	3742	3742	3592	3556	2914
Period (27)	11pm-12am	2322	2880	3096	3096	2972	3234	2648
Period (28)	12-1 am	2236	2774	2982	2982	2864	3242	2656
Period (28)	1-2 am	2098	2600	2796	2796	2684	3066	2512
Period (28)	2-3 am	2028	2514	2702	2702	2594	2892	2368
Period (28)	3-4 am	1958	2426	2610	2610	2506	2848	2332
Period	4-5 am	1958	2426	2610	2610	2506	2804	2296

(28)								
Period (28)	5-6 am	2028	2514	2702	2702	2594	2716	2224
Period (28)	6-7 am	2236	2774	2982	2982	2864	2716	2224
Period (28)	7-8 am	2656	3294	3542	3542	3400	2892	2368
Period (28)	8-9 am	3040	3770	4054	4054	3892	3548	2906
Period (28)	9-10 am	3320	4118	4428	4428	4250	3768	3086
Period (28)	10-11am	3460	4290	4614	4614	4428	3986	3266
Period (28)	11am-12 pm	3496	4334	4660	4660	4474	4074	3338
Period (28)	12-1 pm	3460	4290	4614	4614	4428	4074	3338
Period (28)	1-2 pm	3496	4334	4660	4660	4474	4030	3302
Period (28)	2-3 pm	3496	4334	4660	4660	4474	3986	3266
Period (28)	3-4 pm	3390	4204	4520	4520	4340	3986	3266
Period (28)	4-5 pm	3356	4160	4474	4474	4294	4030	3302
Period (28)	5-6 pm	3356	4160	4474	4474	4294	4118	3372
Period (28)	6-7 pm	3250	4030	4334	4334	4160	4162	3408
Period (28)	7-8 pm	3216	3988	4288	4288	4116	4162	3408
Period (28)	8-9 pm	3216	3988	4288	4288	4116	4380	3588
Period (28)	9-10 pm	3250	4030	4334	4334	4160	4074	3338
Period (28)	10-11 pm	3040	3770	4054	4054	3892	3854	3158
Period (28)	11pm-12am	2516	3120	3356	3356	3220	3504	2870
Period (29)	12-1 am	2188	2714	2918	2918	2802	3172	2598
Period (29)	1-2 am	2052	2544	2736	2736	2626	3000	2458
Period (29)	2-3 am	1984	2460	2644	2644	2540	2830	2318
Period (29)	3-4 am	1916	2374	2554	2554	2452	2786	2282
Period (29)	4-5 am	1916	2374	2554	2554	2452	2744	2248
Period (29)	5-6 am	1984	2460	2644	2644	2540	2658	2176
Period (29)	6-7 am	2188	2714	2918	2918	2802	2658	2176
Period (29)	7-8 am	2600	3224	3466	3466	3326	2830	2318
Period (29)	8-9 am	2976	3690	3968	3968	3808	3472	2844
Period (29)	9-10 am	3250	4028	4332	4332	4158	3686	3020
Period (29)	10-11am	3386	4198	4514	4514	4334	3900	3196
Period (29)	11am-12 pm	3420	4240	4560	4560	4378	3986	3266
Period (29)	12-1 pm	3386	4198	4514	4514	4334	3986	3266
Period (29)	1-2 pm	3420	4240	4560	4560	4378	3944	3230
Period (29)	2-3 pm	3420	4240	4560	4560	4378	3900	3196
Period (29)	3-4 pm	3318	4114	4424	4424	4246	3900	3196
Period (29)	4-5 pm	3284	4072	4378	4378	4202	3944	3230

Period (29)	5-6 pm	3284	4072	4378	4378	4202	4030	3300
Period (29)	6-7 pm	3180	3944	4240	4240	4072	4072	3336
Period (29)	7-8 pm	3146	3902	4196	4196	4028	4072	3336
Period (29)	8-9 pm	3146	3902	4196	4196	4028	4286	3512
Period (29)	9-10 pm	3180	3944	4240	4240	4072	3986	3266
Period (29)	10-11 pm	2976	3690	3968	3968	3808	3772	3090
Period (29)	11pm-12am	2462	3054	3284	3284	3152	3430	2808
Period (30)	12-1 am	2410	2988	3212	3212	3084	3492	2860
Period (30)	1-2 am	2260	2802	3012	3012	2892	3304	2706
Period (30)	2-3 am	2184	2708	2912	2912	2796	3114	2552
Period (30)	3-4 am	2108	2614	2812	2812	2698	3068	2512
Period (30)	4-5 am	2108	2614	2812	2812	2698	3020	2474
Period (30)	5-6 am	2184	2708	2912	2912	2796	2926	2396
Period (30)	6-7 am	2410	2988	3212	3212	3084	2926	2396
Period (30)	7-8 am	2862	3548	3816	3816	3662	3114	2552
Period (30)	8-9 am	3276	4062	4368	4368	4192	3822	3130
Period (30)	9-10 am	3576	4436	4770	4770	4578	4058	3324
Period (30)	10-11am	3728	4622	4970	4970	4772	4294	3518
Period (30)	11am-12 pm	3766	4668	5020	5020	4820	4388	3594
Period (30)	12-1 pm	3728	4622	4970	4970	4772	4388	3594
Period (30)	1-2 pm	3766	4668	5020	5020	4820	4342	3556
Period (30)	2-3 pm	3766	4668	5020	5020	4820	4294	3518
Period (30)	3-4 pm	3652	4528	4870	4870	4674	4294	3518
Period (30)	4-5 pm	3614	4482	4820	4820	4626	4342	3556
Period (30)	5-6 pm	3614	4482	4820	4820	4626	4436	3634
Period (30)	6-7 pm	3502	4342	4668	4668	4482	4482	3672
Period (30)	7-8 pm	3464	4296	4618	4618	4434	4482	3672
Period (30)	8-9 pm	3464	4296	4618	4618	4434	4718	3866
Period (30)	9-10 pm	3502	4342	4668	4668	4482	4388	3594
Period (30)	10-11 pm	3276	4062	4368	4368	4192	4152	3402
Period (30)	11pm-12am	2710	3362	3614	3614	3470	3776	3092
Period (31)	12-1 am	1946	2414	2596	2596	2492	2904	2380
Period (31)	1-2 am	1916	2376	2554	2554	2452	2828	2316
Period (31)	2-3 am	1854	2300	2472	2472	2374	2672	2188
Period (31)	3-4 am	1792	2222	2390	2390	2294	2556	2094
Period (31)	4-5 am	1824	2260	2430	2430	2334	2518	2062
Period	5-6 am	2008	2490	2678	2678	2570	2518	2062

(31)								
Period (31)	6-7 am	2224	2760	2966	2966	2848	2634	2158
Period (31)	7-8 am	2626	3258	3502	3502	3362	2866	2348
Period (31)	8-9 am	2936	3640	3914	3914	3758	3214	2634
Period (31)	9-10 am	3060	3794	4078	4078	3916	3446	2824
Period (31)	10-11am	3090	3832	4120	4120	3956	3562	2918
Period (31)	11am-12 pm	3060	3794	4078	4078	3916	3640	2982
Period (31)	12-1 pm	2874	3564	3832	3832	3678	3524	2886
Period (31)	1-2 pm	2842	3526	3790	3790	3638	3486	2856
Period (31)	2-3 pm	2782	3448	3708	3708	3560	3486	2856
Period (31)	3-4 pm	2720	3372	3626	3626	3480	3330	2728
Period (31)	4-5 pm	2782	3448	3708	3708	3560	3292	2696
Period (31)	5-6 pm	2842	3526	3790	3790	3638	3408	2792
Period (31)	6-7 pm	2966	3678	3956	3956	3796	3562	2918
Period (31)	7-8 pm	3028	3756	4038	4038	3876	3872	3172
Period (31)	8-9 pm	2966	3678	3956	3956	3796	3756	3078
Period (31)	9-10 pm	2782	3448	3708	3708	3560	3680	3014
Period (31)	10-11 pm	2472	3066	3296	3296	3164	3486	2856
Period (31)	11pm-12am	2164	2682	2884	2884	2768	3292	2696
Period (32)	12-1 am	2088	2590	2784	2784	2674	3116	2552
Period (32)	1-2 am	2056	2548	2740	2740	2630	3034	2484
Period (32)	2-3 am	1990	2466	2652	2652	2546	2866	2348
Period (32)	3-4 am	1922	2384	2564	2564	2462	2742	2246
Period (32)	4-5 am	1956	2424	2608	2608	2504	2700	2212
Period (32)	5-6 am	2154	2672	2874	2874	2758	2700	2212
Period (32)	6-7 am	2386	2960	3182	3182	3056	2826	2314
Period (32)	7-8 am	2818	3494	3758	3758	3606	3074	2518
Period (32)	8-9 am	3150	3904	4200	4200	4032	3448	2824
Period (32)	9-10 am	3282	4068	4376	4376	4200	3698	3030
Period (32)	10-11am	3316	4110	4420	4420	4244	3822	3132
Period (32)	11am-12 pm	3282	4068	4376	4376	4200	3906	3200
Period (32)	12-1 pm	3082	3822	4110	4110	3946	3780	3098
Period (32)	1-2 pm	3050	3782	4066	4066	3904	3740	3064
Period (32)	2-3 pm	2984	3700	3978	3978	3818	3740	3064
Period (32)	3-4 pm	2918	3616	3890	3890	3734	3574	2926
Period (32)	4-5 pm	2984	3700	3978	3978	3818	3532	2892
Period (32)	5-6 pm	3050	3782	4066	4066	3904	3656	2994

Period (32)	6-7 pm	3182	3946	4244	4244	4074	3822	3132
Period (32)	7-8 pm	3248	4028	4332	4332	4158	4154	3404
Period (32)	8-9 pm	3182	3946	4244	4244	4074	4030	3302
Period (32)	9-10 pm	2984	3700	3978	3978	3818	3948	3234
Period (32)	10-11 pm	2652	3288	3536	3536	3394	3740	3064
Period (32)	11pm-12am	2320	2878	3094	3094	2970	3532	2892
Period (33)	12-1 am	2154	2672	2872	2872	2758	3214	2634
Period (33)	1-2 am	2120	2628	2828	2828	2714	3130	2564
Period (33)	2-3 am	2052	2544	2736	2736	2626	2958	2422
Period (33)	3-4 am	1984	2460	2644	2644	2540	2830	2318
Period (33)	4-5 am	2018	2502	2690	2690	2582	2786	2282
Period (33)	5-6 am	2224	2756	2964	2964	2846	2786	2282
Period (33)	6-7 am	2462	3052	3284	3284	3152	2914	2388
Period (33)	7-8 am	2908	3604	3876	3876	3720	3172	2598
Period (33)	8-9 am	3250	4028	4332	4332	4158	3558	2914
Period (33)	9-10 am	3386	4198	4514	4514	4334	3814	3124
Period (33)	10-11am	3420	4240	4560	4560	4378	3944	3230
Period (33)	11am-12 pm	3386	4198	4514	4514	4334	4030	3300
Period (33)	12-1 pm	3180	3944	4240	4240	4072	3900	3196
Period (33)	1-2 pm	3146	3900	4196	4196	4028	3858	3160
Period (33)	2-3 pm	3078	3816	4104	4104	3940	3858	3160
Period (33)	3-4 pm	3010	3732	4012	4012	3852	3686	3020
Period (33)	4-5 pm	3078	3816	4104	4104	3940	3644	2984
Period (33)	5-6 pm	3146	3900	4196	4196	4028	3772	3090
Period (33)	6-7 pm	3284	4070	4378	4378	4202	3944	3230
Period (33)	7-8 pm	3352	4156	4468	4468	4290	4286	3512
Period (33)	8-9 pm	3284	4070	4378	4378	4202	4158	3406
Period (33)	9-10 pm	3078	3816	4104	4104	3940	4072	3336
Period (33)	10-11 pm	2736	3392	3648	3648	3502	3858	3160
Period (33)	11pm-12am	2394	2968	3192	3192	3064	3644	2984
Period (34)	12-1 am	1966	2436	2620	2620	2516	2932	2402
Period (34)	1-2 am	1934	2398	2580	2580	2476	2854	2338
Period (34)	2-3 am	1872	2320	2496	2496	2396	2698	2210
Period (34)	3-4 am	1810	2244	2412	2412	2316	2580	2114
Period (34)	4-5 am	1840	2282	2454	2454	2356	2542	2082
Period (34)	5-6 am	2028	2514	2704	2704	2596	2542	2082
Period	6-7 am	2246	2784	2996	2996	2876	2658	2178

(34)								
Period (34)	7-8 am	2652	3288	3536	3536	3394	2894	2370
Period (34)	8-9 am	2964	3674	3952	3952	3794	3246	2658
Period (34)	9-10 am	3088	3830	4118	4118	3954	3480	2850
Period (34)	10-11am	3120	3868	4160	4160	3994	3598	2946
Period (34)	11am-12 pm	3088	3830	4118	4118	3954	3676	3012
Period (34)	12-1 pm	2902	3598	3868	3868	3714	3558	2914
Period (34)	1-2 pm	2870	3558	3828	3828	3674	3520	2882
Period (34)	2-3 pm	2808	3482	3744	3744	3594	3520	2882
Period (34)	3-4 pm	2746	3404	3660	3660	3514	3362	2754
Period (34)	4-5 pm	2808	3482	3744	3744	3594	3324	2722
Period (34)	5-6 pm	2870	3558	3828	3828	3674	3440	2818
Period (34)	6-7 pm	2996	3714	3994	3994	3834	3598	2946
Period (34)	7-8 pm	3058	3790	4076	4076	3914	3910	3204
Period (34)	8-9 pm	2996	3714	3994	3994	3834	3792	3108
Period (34)	9-10 pm	2808	3482	3744	3744	3594	3714	3044
Period (34)	10-11 pm	2496	3094	3328	3328	3194	3520	2882
Period (34)	11pm-12am	2184	2708	2912	2912	2796	3324	2722
Period (35)	12-1 am	1956	2426	2608	2608	2504	2920	2390
Period (35)	1-2 am	1926	2388	2566	2566	2464	2842	2328
Period (35)	2-3 am	1864	2310	2484	2484	2384	2686	2200
Period (35)	3-4 am	1800	2234	2402	2402	2306	2568	2104
Period (35)	4-5 am	1832	2272	2442	2442	2344	2530	2072
Period (35)	5-6 am	2018	2502	2692	2692	2584	2530	2072
Period (35)	6-7 am	2236	2772	2980	2980	2862	2646	2168
Period (35)	7-8 am	2640	3272	3520	3520	3378	2880	2358
Period (35)	8-9 am	2950	3658	3934	3934	3776	3230	2646
Period (35)	9-10 am	3074	3812	4098	4098	3934	3464	2838
Period (35)	10-11am	3106	3850	4140	4140	3974	3580	2932
Period (35)	11am-12 pm	3074	3812	4098	4098	3934	3658	2996
Period (35)	12-1 pm	2888	3580	3850	3850	3696	3542	2900
Period (35)	1-2 pm	2856	3542	3808	3808	3656	3502	2870
Period (35)	2-3 pm	2794	3466	3726	3726	3576	3502	2870
Period (35)	3-4 pm	2732	3388	3644	3644	3498	3348	2742
Period (35)	4-5 pm	2794	3466	3726	3726	3576	3308	2710
Period (35)	5-6 pm	2856	3542	3808	3808	3656	3424	2806
Period (35)	6-7 pm	2980	3696	3974	3974	3816	3580	2932

Period (35)	7-8 pm	3042	3774	4058	4058	3894	3892	3188
Period (35)	8-9 pm	2980	3696	3974	3974	3816	3776	3092
Period (35)	9-10 pm	2794	3466	3726	3726	3576	3698	3028
Period (35)	10-11 pm	2484	3080	3312	3312	3180	3502	2870
Period (35)	11pm-12am	2174	2696	2898	2898	2782	3308	2710
Period (36)	12-1 am	1900	2354	2532	2532	2432	2834	2322
Period (36)	1-2 am	1870	2318	2492	2492	2392	2758	2260
Period (36)	2-3 am	1810	2242	2412	2412	2316	2606	2136
Period (36)	3-4 am	1748	2168	2332	2332	2238	2494	2042
Period (36)	4-5 am	1778	2206	2372	2372	2276	2456	2012
Period (36)	5-6 am	1960	2430	2614	2614	2508	2456	2012
Period (36)	6-7 am	2170	2692	2894	2894	2778	2570	2104
Period (36)	7-8 am	2562	3178	3418	3418	3280	2796	2290
Period (36)	8-9 am	2864	3552	3820	3820	3666	3136	2570
Period (36)	9-10 am	2984	3700	3980	3980	3820	3362	2754
Period (36)	10-11am	3016	3738	4020	4020	3860	3476	2848
Period (36)	11am-12 pm	2984	3700	3980	3980	3820	3552	2910
Period (36)	12-1 pm	2804	3476	3738	3738	3590	3438	2816
Period (36)	1-2 pm	2774	3438	3698	3698	3550	3400	2786
Period (36)	2-3 pm	2714	3364	3618	3618	3474	3400	2786
Period (36)	3-4 pm	2654	3290	3538	3538	3396	3250	2662
Period (36)	4-5 pm	2714	3364	3618	3618	3474	3212	2632
Period (36)	5-6 pm	2774	3438	3698	3698	3550	3324	2724
Period (36)	6-7 pm	2894	3588	3860	3860	3704	3476	2848
Period (36)	7-8 pm	2954	3664	3940	3940	3782	3778	3096
Period (36)	8-9 pm	2894	3588	3860	3860	3704	3664	3002
Period (36)	9-10 pm	2714	3364	3618	3618	3474	3590	2940
Period (36)	10-11 pm	2412	2990	3216	3216	3088	3400	2786
Period (36)	11pm-12am	2110	2616	2814	2814	2702	3212	2632
Period (37)	12-1 am	2098	2602	2798	2798	2686	3130	2564
Period (37)	1-2 am	2064	2560	2752	2752	2642	3048	2496
Period (37)	2-3 am	1998	2478	2664	2664	2558	2880	2358
Period (37)	3-4 am	1932	2396	2576	2576	2472	2754	2256
Period (37)	4-5 am	1964	2436	2620	2620	2514	2714	2222
Period (37)	5-6 am	2164	2684	2886	2886	2770	2714	2222
Period (37)	6-7 am	2398	2974	3196	3196	3068	2838	2324
Period	7-8 am	2830	3510	3774	3774	3624	3088	2530

(37)								
Period (37)	8-9 am	3164	3924	4218	4218	4050	3464	2838
Period (37)	9-10 am	3296	4088	4396	4396	4220	3714	3042
Period (37)	10-11am	3330	4130	4440	4440	4262	3840	3146
Period (37)	11am-12 pm	3296	4088	4396	4396	4220	3924	3214
Period (37)	12-1 pm	3096	3840	4130	4130	3964	3798	3112
Period (37)	1-2 pm	3064	3800	4084	4084	3922	3756	3076
Period (37)	2-3 pm	2998	3718	3996	3996	3836	3756	3076
Period (37)	3-4 pm	2930	3634	3908	3908	3750	3590	2940
Period (37)	4-5 pm	2998	3718	3996	3996	3836	3548	2906
Period (37)	5-6 pm	3064	3800	4084	4084	3922	3674	3008
Period (37)	6-7 pm	3196	3964	4262	4262	4092	3840	3146
Period (37)	7-8 pm	3264	4048	4352	4352	4178	4174	3418
Period (37)	8-9 pm	3196	3964	4262	4262	4092	4048	3316
Period (37)	9-10 pm	2998	3718	3996	3996	3836	3966	3248
Period (37)	10-11 pm	2664	3304	3552	3552	3410	3756	3076
Period (37)	11pm-12am	2332	2892	3108	3108	2984	3548	2906
Period (38)	12-1 am	1872	2320	2494	2494	2396	2792	2286
Period (38)	1-2 am	1842	2282	2456	2456	2356	2718	2226
Period (38)	2-3 am	1782	2210	2376	2376	2280	2568	2104
Period (38)	3-4 am	1722	2136	2296	2296	2204	2456	2012
Period (38)	4-5 am	1752	2172	2336	2336	2242	2420	1982
Period (38)	5-6 am	1930	2394	2574	2574	2472	2420	1982
Period (38)	6-7 am	2138	2652	2852	2852	2738	2530	2074
Period (38)	7-8 am	2524	3130	3366	3366	3232	2754	2256
Period (38)	8-9 am	2822	3498	3762	3762	3612	3090	2530
Period (38)	9-10 am	2940	3646	3920	3920	3764	3312	2714
Period (38)	10-11am	2970	3682	3960	3960	3802	3424	2806
Period (38)	11am-12 pm	2940	3646	3920	3920	3764	3498	2866
Period (38)	12-1 pm	2762	3424	3682	3682	3536	3388	2774
Period (38)	1-2 pm	2732	3388	3644	3644	3498	3350	2744
Period (38)	2-3 pm	2674	3314	3564	3564	3422	3350	2744
Period (38)	3-4 pm	2614	3240	3484	3484	3346	3200	2622
Period (38)	4-5 pm	2674	3314	3564	3564	3422	3164	2592
Period (38)	5-6 pm	2732	3388	3644	3644	3498	3276	2684
Period (38)	6-7 pm	2852	3534	3802	3802	3650	3424	2806
Period (38)	7-8 pm	2910	3608	3880	3880	3726	3722	3050

Period (38)	8-9 pm	2852	3534	3802	3802	3650	3610	2958
Period (38)	9-10 pm	2674	3314	3564	3564	3422	3536	2896
Period (38)	10-11 pm	2376	2946	3168	3168	3042	3350	2744
Period (38)	11pm-12am	2080	2578	2772	2772	2662	3164	2592
Period (39)	12-1 am	1946	2414	2596	2596	2492	2904	2380
Period (39)	1-2 am	1916	2376	2554	2554	2452	2826	2316
Period (39)	2-3 am	1854	2300	2472	2472	2374	2672	2188
Period (39)	3-4 am	1792	2222	2390	2390	2294	2556	2094
Period (39)	4-5 am	1824	2260	2430	2430	2334	2516	2062
Period (39)	5-6 am	2008	2490	2678	2678	2570	2516	2062
Period (39)	6-7 am	2224	2760	2966	2966	2848	2632	2158
Period (39)	7-8 am	2626	3258	3502	3502	3362	2866	2348
Period (39)	8-9 am	2936	3640	3914	3914	3758	3214	2634
Period (39)	9-10 am	3060	3794	4078	4078	3916	3446	2824
Period (39)	10-11am	3090	3832	4120	4120	3956	3562	2918
Period (39)	11am-12 pm	3060	3794	4078	4078	3916	3640	2982
Period (39)	12-1 pm	2874	3564	3832	3832	3678	3524	2886
Period (39)	1-2 pm	2842	3526	3790	3790	3638	3484	2856
Period (39)	2-3 pm	2782	3448	3708	3708	3560	3484	2856
Period (39)	3-4 pm	2720	3372	3626	3626	3480	3330	2728
Period (39)	4-5 pm	2782	3448	3708	3708	3560	3292	2696
Period (39)	5-6 pm	2842	3526	3790	3790	3638	3408	2792
Period (39)	6-7 pm	2966	3678	3956	3956	3796	3562	2918
Period (39)	7-8 pm	3028	3756	4038	4038	3876	3872	3172
Period (39)	8-9 pm	2966	3678	3956	3956	3796	3756	3078
Period (39)	9-10 pm	2782	3448	3708	3708	3560	3678	3014
Period (39)	10-11 pm	2472	3066	3296	3296	3164	3484	2856
Period (39)	11pm-12am	2164	2682	2884	2884	2768	3292	2696
Period (40)	12-1 am	1946	2414	2596	2596	2492	2904	2380
Period (40)	1-2 am	1916	2376	2554	2554	2452	2826	2316
Period (40)	2-3 am	1854	2300	2472	2472	2374	2672	2188
Period (40)	3-4 am	1792	2222	2390	2390	2294	2556	2094
Period (40)	4-5 am	1824	2260	2430	2430	2334	2516	2062
Period (40)	5-6 am	2008	2490	2678	2678	2570	2516	2062
Period (40)	6-7 am	2224	2760	2966	2966	2848	2632	2158
Period (40)	7-8 am	2626	3258	3502	3502	3362	2866	2348
Period	8-9 am	2936	3640	3914	3914	3758	3214	2634

(40)								
Period (40)	9-10 am	3060	3794	4078	4078	3916	3446	2824
Period (40)	10-11am	3090	3832	4120	4120	3956	3562	2918
Period (40)	11am-12 pm	3060	3794	4078	4078	3916	3640	2982
Period (40)	12-1 pm	2874	3564	3832	3832	3678	3524	2886
Period (40)	1-2 pm	2842	3526	3790	3790	3638	3484	2856
Period (40)	2-3 pm	2782	3448	3708	3708	3560	3484	2856
Period (40)	3-4 pm	2720	3372	3626	3626	3480	3330	2728
Period (40)	4-5 pm	2782	3448	3708	3708	3560	3292	2696
Period (40)	5-6 pm	2842	3526	3790	3790	3638	3408	2792
Period (40)	6-7 pm	2966	3678	3956	3956	3796	3562	2918
Period (40)	7-8 pm	3028	3756	4038	4038	3876	3872	3172
Period (40)	8-9 pm	2966	3678	3956	3956	3796	3756	3078
Period (40)	9-10 pm	2782	3448	3708	3708	3560	3678	3014
Period (40)	10-11 pm	2472	3066	3296	3296	3164	3484	2856
Period (40)	11pm-12am	2164	2682	2884	2884	2768	3292	2696
Period (41)	12-1 am	2004	2484	2672	2672	2564	2990	2448
Period (41)	1-2 am	1972	2446	2628	2628	2524	2910	2384
Period (41)	2-3 am	1908	2366	2544	2544	2442	2750	2252
Period (41)	3-4 am	1844	2288	2460	2460	2360	2630	2154
Period (41)	4-5 am	1876	2326	2502	2502	2402	2590	2122
Period (41)	5-6 am	2068	2564	2756	2756	2646	2590	2122
Period (41)	6-7 am	2290	2840	3052	3052	2930	2710	2220
Period (41)	7-8 am	2704	3352	3604	3604	3460	2950	2416
Period (41)	8-9 am	3022	3746	4028	4028	3866	3308	2710
Period (41)	9-10 am	3148	3904	4198	4198	4030	3548	2906
Period (41)	10-11am	3180	3944	4240	4240	4070	3668	3004
Period (41)	11am-12 pm	3148	3904	4198	4198	4030	3746	3068
Period (41)	12-1 pm	2958	3668	3944	3944	3786	3628	2970
Period (41)	1-2 pm	2926	3628	3900	3900	3744	3588	2938
Period (41)	2-3 pm	2862	3550	3816	3816	3664	3588	2938
Period (41)	3-4 pm	2798	3470	3732	3732	3582	3428	2808
Period (41)	4-5 pm	2862	3550	3816	3816	3664	3388	2776
Period (41)	5-6 pm	2926	3628	3900	3900	3744	3508	2874
Period (41)	6-7 pm	3052	3786	4070	4070	3908	3668	3004
Period (41)	7-8 pm	3116	3866	4156	4156	3988	3986	3264
Period (41)	8-9 pm	3052	3786	4070	4070	3908	3866	3166

Period (41)	9-10 pm	2862	3550	3816	3816	3664	3786	3102
Period (41)	10-11 pm	2544	3156	3392	3392	3256	3588	2938
Period (41)	11pm-12am	2226	2760	2968	2968	2850	3388	2776
Period (42)	12-1 am	2004	2484	2672	2672	2564	2990	2448
Period (42)	1-2 am	1972	2446	2628	2628	2524	2910	2384
Period (42)	2-3 am	1908	2366	2544	2544	2442	2750	2252
Period (42)	3-4 am	1844	2288	2460	2460	2360	2630	2154
Period (42)	4-5 am	1876	2326	2502	2502	2402	2590	2122
Period (42)	5-6 am	2068	2564	2756	2756	2646	2590	2122
Period (42)	6-7 am	2290	2840	3052	3052	2930	2710	2220
Period (42)	7-8 am	2704	3352	3604	3604	3460	2950	2416
Period (42)	8-9 am	3022	3746	4028	4028	3866	3308	2710
Period (42)	9-10 am	3148	3904	4198	4198	4030	3548	2906
Period (42)	10-11am	3180	3944	4240	4240	4070	3668	3004
Period (42)	11am-12 pm	3148	3904	4198	4198	4030	3746	3068
Period (42)	12-1 pm	2958	3668	3944	3944	3786	3628	2970
Period (42)	1-2 pm	2926	3628	3900	3900	3744	3588	2938
Period (42)	2-3 pm	2862	3550	3816	3816	3664	3588	2938
Period (42)	3-4 pm	2798	3470	3732	3732	3582	3428	2808
Period (42)	4-5 pm	2862	3550	3816	3816	3664	3388	2776
Period (42)	5-6 pm	2926	3628	3900	3900	3744	3508	2874
Period (42)	6-7 pm	3052	3786	4070	4070	3908	3668	3004
Period (42)	7-8 pm	3116	3866	4156	4156	3988	3986	3264
Period (42)	8-9 pm	3052	3786	4070	4070	3908	3866	3166
Period (42)	9-10 pm	2862	3550	3816	3816	3664	3786	3102
Period (42)	10-11 pm	2544	3156	3392	3392	3256	3588	2938
Period (42)	11pm-12am	2226	2760	2968	2968	2850	3388	2776
Period (43)	12-1 am	2154	2672	2872	2872	2758	3214	2634
Period (43)	1-2 am	2120	2628	2828	2828	2714	3128	2564
Period (43)	2-3 am	2052	2544	2736	2736	2626	2958	2422
Period (43)	3-4 am	1984	2460	2644	2644	2540	2828	2318
Period (43)	4-5 am	2018	2502	2690	2690	2582	2786	2282
Period (43)	5-6 am	2224	2756	2964	2964	2846	2786	2282
Period (43)	6-7 am	2462	3052	3284	3284	3152	2914	2388
Period (43)	7-8 am	2908	3604	3876	3876	3720	3172	2598
Period (43)	8-9 am	3250	4028	4332	4332	4158	3558	2914
Period	9-10 am	3386	4198	4514	4514	4334	3814	3124

(43)								
Period (43)	10-11am	3420	4240	4560	4560	4378	3944	3230
Period (43)	11am-12 pm	3386	4198	4514	4514	4334	4028	3300
Period (43)	12-1 pm	3180	3944	4240	4240	4072	3900	3196
Period (43)	1-2 pm	3146	3900	4196	4196	4028	3858	3160
Period (43)	2-3 pm	3078	3816	4104	4104	3940	3858	3160
Period (43)	3-4 pm	3010	3732	4012	4012	3852	3686	3020
Period (43)	4-5 pm	3078	3816	4104	4104	3940	3644	2984
Period (43)	5-6 pm	3146	3900	4196	4196	4028	3772	3090
Period (43)	6-7 pm	3284	4070	4378	4378	4202	3944	3230
Period (43)	7-8 pm	3352	4156	4468	4468	4290	4286	3512
Period (43)	8-9 pm	3284	4070	4378	4378	4202	4158	3406
Period (43)	9-10 pm	3078	3816	4104	4104	3940	4072	3336
Period (43)	10-11 pm	2736	3392	3648	3648	3502	3858	3160
Period (43)	11pm-12am	2394	2968	3192	3192	3064	3644	2984
Period (44)	12-1 am	2522	3128	3364	3364	3228	3680	3016
Period (44)	1-2 am	2372	2942	3162	3162	3036	3398	2784
Period (44)	2-3 am	2260	2802	3012	3012	2892	3208	2628
Period (44)	3-4 am	2222	2754	2962	2962	2844	3114	2552
Period (44)	4-5 am	2222	2754	2962	2962	2844	3020	2474
Period (44)	5-6 am	2260	2802	3012	3012	2892	3068	2512
Period (44)	6-7 am	2786	3454	3714	3714	3566	3114	2552
Period (44)	7-8 am	3238	4014	4318	4318	4144	3304	2706
Period (44)	8-9 am	3576	4436	4770	4770	4578	3776	3092
Period (44)	9-10 am	3614	4482	4820	4820	4626	4152	3402
Period (44)	10-11am	3614	4482	4820	4820	4626	4246	3478
Period (44)	11am-12 pm	3576	4436	4770	4770	4578	4294	3518
Period (44)	12-1 pm	3576	4436	4770	4770	4578	4246	3478
Period (44)	1-2 pm	3576	4436	4770	4770	4578	4152	3402
Period (44)	2-3 pm	3502	4342	4668	4668	4482	4106	3362
Period (44)	3-4 pm	3540	4388	4718	4718	4530	4106	3362
Period (44)	4-5 pm	3728	4622	4970	4970	4772	4294	3518
Period (44)	5-6 pm	3766	4668	5020	5020	4820	4718	3866
Period (44)	6-7 pm	3766	4668	5020	5020	4820	4672	3826
Period (44)	7-8 pm	3614	4482	4820	4820	4626	4578	3750
Period (44)	8-9 pm	3426	4248	4568	4568	4386	4436	3634
Period (44)	9-10 pm	3124	3874	4166	4166	4000	4342	3556

Period (44)	10-11 pm	2748	3408	3664	3664	3518	4106	3362
Period (44)	11pm-12am	2372	2942	3162	3162	3036	3822	3130
Period (45)	12-1 am	2532	3140	3364	3364	3242	3696	3028
Period (45)	1-2 am	2382	2952	3162	3162	3048	3412	2794
Period (45)	2-3 am	2268	2812	3012	3012	2904	3222	2638
Period (45)	3-4 am	2230	2766	2962	2962	2854	3126	2562
Period (45)	4-5 am	2230	2766	2962	2962	2854	3032	2484
Period (45)	5-6 am	2268	2812	3012	3012	2904	3080	2522
Period (45)	6-7 am	2798	3468	3714	3714	3580	3126	2562
Period (45)	7-8 am	3250	4030	4318	4318	4162	3316	2716
Period (45)	8-9 am	3592	4452	4770	4770	4596	3790	3104
Period (45)	9-10 am	3628	4500	4820	4820	4644	4170	3416
Period (45)	10-11am	3628	4500	4820	4820	4644	4264	3492
Period (45)	11am-12 pm	3592	4452	4770	4770	4596	4312	3532
Period (45)	12-1 pm	3592	4452	4770	4770	4596	4264	3492
Period (45)	1-2 pm	3592	4452	4770	4770	4596	4170	3416
Period (45)	2-3 pm	3516	4360	4668	4668	4500	4122	3376
Period (45)	3-4 pm	3554	4406	4718	4718	4548	4122	3376
Period (45)	4-5 pm	3742	4640	4970	4970	4790	4312	3532
Period (45)	5-6 pm	3780	4688	5020	5020	4838	4738	3880
Period (45)	6-7 pm	3780	4688	5020	5020	4838	4690	3842
Period (45)	7-8 pm	3628	4500	4820	4820	4644	4596	3764
Period (45)	8-9 pm	3440	4266	4568	4568	4402	4454	3648
Period (45)	9-10 pm	3138	3890	4166	4166	4016	4358	3570
Period (45)	10-11 pm	2760	3422	3664	3664	3532	4122	3376
Period (45)	11pm-12am	2382	2952	3162	3162	3048	3838	3144
Period (46)	12-1 am	2602	3228	3470	3470	3332	3798	3112
Period (46)	1-2 am	2448	3034	3264	3264	3132	3506	2872
Period (46)	2-3 am	2332	2890	3108	3108	2984	3312	2712
Period (46)	3-4 am	2292	2842	3056	3056	2934	3214	2632
Period (46)	4-5 am	2292	2842	3056	3056	2934	3116	2552
Period (46)	5-6 am	2332	2890	3108	3108	2984	3164	2592
Period (46)	6-7 am	2874	3564	3834	3834	3680	3214	2632
Period (46)	7-8 am	3342	4142	4454	4454	4276	3408	2792
Period (46)	8-9 am	3690	4576	4922	4922	4724	3896	3190
Period (46)	9-10 am	3730	4624	4972	4972	4774	4284	3510
Period	10-11am	3730	4624	4972	4972	4774	4382	3590

(46)								
Period (46)	11am-12 pm	3690	4576	4922	4922	4724	4430	3630
Period (46)	12-1 pm	3690	4576	4922	4922	4724	4382	3590
Period (46)	1-2 pm	3690	4576	4922	4922	4724	4284	3510
Period (46)	2-3 pm	3614	4480	4818	4818	4624	4236	3470
Period (46)	3-4 pm	3652	4528	4870	4870	4674	4236	3470
Period (46)	4-5 pm	3846	4770	5128	5128	4924	4430	3630
Period (46)	5-6 pm	3886	4818	5180	5180	4972	4870	3988
Period (46)	6-7 pm	3886	4818	5180	5180	4972	4820	3948
Period (46)	7-8 pm	3730	4624	4972	4972	4774	4724	3868
Period (46)	8-9 pm	3536	4384	4714	4714	4526	4578	3750
Period (46)	9-10 pm	3224	3998	4300	4300	4128	4480	3670
Period (46)	10-11 pm	2836	3516	3782	3782	3630	4236	3470
Period (46)	11pm-12am	2448	3034	3264	3264	3132	3944	3230
Period (47)	12-1 am	2694	3340	3592	3592	3448	3930	3220
Period (47)	1-2 am	2532	3140	3376	3376	3242	3628	2972
Period (47)	2-3 am	2412	2990	3216	3216	3088	3426	2806
Period (47)	3-4 am	2372	2942	3162	3162	3036	3326	2724
Period (47)	4-5 am	2372	2942	3162	3162	3036	3224	2642
Period (47)	5-6 am	2412	2990	3216	3216	3088	3274	2682
Period (47)	6-7 am	2974	3688	3966	3966	3808	3326	2724
Period (47)	7-8 am	3458	4286	4610	4610	4426	3526	2890
Period (47)	8-9 am	3820	4736	5092	5092	4888	4030	3302
Period (47)	9-10 am	3860	4786	5146	5146	4940	4434	3632
Period (47)	10-11am	3860	4786	5146	5146	4940	4534	3714
Period (47)	11am-12 pm	3820	4736	5092	5092	4888	4584	3756
Period (47)	12-1 pm	3820	4736	5092	5092	4888	4534	3714
Period (47)	1-2 pm	3820	4736	5092	5092	4888	4434	3632
Period (47)	2-3 pm	3738	4636	4984	4984	4786	4384	3590
Period (47)	3-4 pm	3778	4686	5038	5038	4836	4384	3590
Period (47)	4-5 pm	3980	4934	5306	5306	5094	4584	3756
Period (47)	5-6 pm	4020	4984	5360	5360	5146	5038	4128
Period (47)	6-7 pm	4020	4984	5360	5360	5146	4988	4086
Period (47)	7-8 pm	3860	4786	5146	5146	4940	4888	4004
Period (47)	8-9 pm	3658	4536	4878	4878	4682	4736	3880
Period (47)	9-10 pm	3336	4138	4448	4448	4270	4636	3798
Period (47)	10-11 pm	2934	3638	3912	3912	3756	4384	3590

Period (47)	11pm-12am	2532	3140	3376	3376	3242	4082	3344
Period (48)	12-1 am	2552	3166	3404	3404	3268	3724	3052
Period (48)	1-2 am	2400	2976	3200	3200	3072	3438	2816
Period (48)	2-3 am	2286	2834	3048	3048	2926	3248	2660
Period (48)	3-4 am	2248	2788	2998	2998	2878	3152	2582
Period (48)	4-5 am	2248	2788	2998	2998	2878	3056	2504
Period (48)	5-6 am	2286	2834	3048	3048	2926	3104	2542
Period (48)	6-7 am	2820	3496	3760	3760	3608	3152	2582
Period (48)	7-8 am	3276	4062	4368	4368	4194	3342	2738
Period (48)	8-9 am	3620	4488	4826	4826	4632	3820	3130
Period (48)	9-10 am	3658	4536	4876	4876	4682	4202	3442
Period (48)	10-11am	3658	4536	4876	4876	4682	4298	3520
Period (48)	11am-12 pm	3620	4488	4826	4826	4632	4346	3560
Period (48)	12-1 pm	3620	4488	4826	4826	4632	4298	3520
Period (48)	1-2 pm	3620	4488	4826	4826	4632	4202	3442
Period (48)	2-3 pm	3544	4394	4724	4724	4536	4154	3404
Period (48)	3-4 pm	3582	4440	4776	4776	4584	4154	3404
Period (48)	4-5 pm	3772	4678	5030	5030	4828	4346	3560
Period (48)	5-6 pm	3810	4724	5080	5080	4876	4776	3912
Period (48)	6-7 pm	3810	4724	5080	5080	4876	4728	3872
Period (48)	7-8 pm	3658	4536	4876	4876	4682	4632	3794
Period (48)	8-9 pm	3468	4300	4622	4622	4438	4488	3676
Period (48)	9-10 pm	3162	3922	4216	4216	4048	4394	3598
Period (48)	10-11 pm	2782	3448	3708	3708	3560	4154	3404
Period (48)	11pm-12am	2400	2976	3200	3200	3072	3868	3168
Period (49)	12-1 am	2694	3340	3592	3592	3448	3930	3220
Period (49)	1-2 am	2532	3140	3376	3376	3242	3628	2972
Period (49)	2-3 am	2412	2990	3216	3216	3088	3426	2806
Period (49)	3-4 am	2372	2942	3162	3162	3036	3326	2724
Period (49)	4-5 am	2372	2942	3162	3162	3036	3224	2642
Period (49)	5-6 am	2412	2990	3216	3216	3088	3274	2682
Period (49)	6-7 am	2974	3688	3966	3966	3808	3326	2724
Period (49)	7-8 am	3458	4286	4610	4610	4426	3526	2890
Period (49)	8-9 am	3820	4736	5092	5092	4888	4030	3302
Period (49)	9-10 am	3860	4786	5146	5146	4940	4434	3632
Period (49)	10-11am	3860	4786	5146	5146	4940	4534	3714
Period	11am-12	3820	4736	5092	5092	4888	4584	3756

(49)	pm							
Period (49)	12-1 pm	3820	4736	5092	5092	4888	4534	3714
Period (49)	1-2 pm	3820	4736	5092	5092	4888	4434	3632
Period (49)	2-3 pm	3738	4636	4984	4984	4786	4384	3590
Period (49)	3-4 pm	3778	4686	5038	5038	4836	4384	3590
Period (49)	4-5 pm	3980	4934	5306	5306	5094	4584	3756
Period (49)	5-6 pm	4020	4984	5360	5360	5146	5038	4128
Period (49)	6-7 pm	4020	4984	5360	5360	5146	4988	4086
Period (49)	7-8 pm	3860	4786	5146	5146	4940	4888	4004
Period (49)	8-9 pm	3658	4536	4878	4878	4682	4736	3880
Period (49)	9-10 pm	3336	4138	4448	4448	4270	4636	3798
Period (49)	10-11 pm	2934	3638	3912	3912	3756	4384	3590
Period (49)	11pm-12am	2532	3140	3376	3376	3242	4082	3344
Period (50)	12-1 am	2774	3440	3698	3698	3550	4048	3316
Period (50)	1-2 am	2608	3234	3478	3478	3338	3736	3060
Period (50)	2-3 am	2484	3080	3312	3312	3180	3528	2890
Period (50)	3-4 am	2442	3028	3256	3256	3126	3424	2806
Period (50)	4-5 am	2442	3028	3256	3256	3126	3320	2720
Period (50)	5-6 am	2484	3080	3312	3312	3180	3372	2762
Period (50)	6-7 am	3064	3798	4084	4084	3922	3424	2806
Period (50)	7-8 am	3560	4414	4748	4748	4558	3632	2976
Period (50)	8-9 am	3934	4876	5244	5244	5034	4152	3400
Period (50)	9-10 am	3974	4928	5300	5300	5088	4566	3740
Period (50)	10-11am	3974	4928	5300	5300	5088	4670	3826
Period (50)	11am-12 pm	3934	4876	5244	5244	5034	4722	3868
Period (50)	12-1 pm	3934	4876	5244	5244	5034	4670	3826
Period (50)	1-2 pm	3934	4876	5244	5244	5034	4566	3740
Period (50)	2-3 pm	3850	4774	5134	5134	4928	4514	3698
Period (50)	3-4 pm	3892	4826	5188	5188	4982	4514	3698
Period (50)	4-5 pm	4098	5082	5464	5464	5246	4722	3868
Period (50)	5-6 pm	4140	5134	5520	5520	5300	5188	4250
Period (50)	6-7 pm	4140	5134	5520	5520	5300	5136	4208
Period (50)	7-8 pm	3974	4928	5300	5300	5088	5034	4122
Period (50)	8-9 pm	3768	4672	5024	5024	4822	4878	3996
Period (50)	9-10 pm	3436	4260	4582	4582	4398	4774	3910
Period (50)	10-11 pm	3022	3748	4030	4030	3868	4514	3698
Period (50)	11pm-12am	2608	3234	3478	3478	3338	4202	3442

Period (51)	12-1 am	2864	3552	3820	3820	3666	4180	3424
Period (51)	1-2 am	2694	3340	3592	3592	3448	3858	3160
Period (51)	2-3 am	2566	3180	3420	3420	3284	3644	2984
Period (51)	3-4 am	2522	3128	3364	3364	3228	3536	2896
Period (51)	4-5 am	2522	3128	3364	3364	3228	3430	2808
Period (51)	5-6 am	2566	3180	3420	3420	3284	3482	2852
Period (51)	6-7 am	3164	3922	4218	4218	4050	3536	2896
Period (51)	7-8 am	3676	4558	4902	4902	4706	3750	3072
Period (51)	8-9 am	4062	5036	5416	5416	5198	4286	3512
Period (51)	9-10 am	4104	5088	5472	5472	5254	4716	3862
Period (51)	10-11am	4104	5088	5472	5472	5254	4822	3950
Period (51)	11am-12 pm	4062	5036	5416	5416	5198	4876	3994
Period (51)	12-1 pm	4062	5036	5416	5416	5198	4822	3950
Period (51)	1-2 pm	4062	5036	5416	5416	5198	4716	3862
Period (51)	2-3 pm	3976	4930	5302	5302	5088	4662	3818
Period (51)	3-4 pm	4018	4982	5358	5358	5144	4662	3818
Period (51)	4-5 pm	4232	5248	5644	5644	5418	4876	3994
Period (51)	5-6 pm	4276	5302	5700	5700	5472	5358	4390
Period (51)	6-7 pm	4276	5302	5700	5700	5472	5304	4346
Period (51)	7-8 pm	4104	5088	5472	5472	5254	5198	4258
Period (51)	8-9 pm	3890	4824	5188	5188	4980	5036	4126
Period (51)	9-10 pm	3548	4400	4732	4732	4542	4930	4038
Period (51)	10-11 pm	3120	3870	4162	4162	3994	4662	3818
Period (51)	11pm-12am	2694	3340	3592	3592	3448	4340	3556
Period (52)	12-1 am	2734	3390	3644	3644	3500	3988	3268
Period (52)	1-2 am	2570	3188	3428	3428	3290	3682	3016
Period (52)	2-3 am	2448	3036	3264	3264	3134	3478	2848
Period (52)	3-4 am	2408	2984	3210	3210	3082	3374	2764
Period (52)	4-5 am	2408	2984	3210	3210	3082	3272	2680
Period (52)	5-6 am	2448	3036	3264	3264	3134	3324	2722
Period (52)	6-7 am	3020	3744	4026	4026	3864	3374	2764
Period (52)	7-8 am	3508	4350	4678	4678	4492	3580	2932
Period (52)	8-9 am	3876	4806	5168	5168	4962	4090	3352
Period (52)	9-10 am	3916	4856	5222	5222	5014	4500	3686
Period (52)	10-11am	3916	4856	5222	5222	5014	4602	3770
Period (52)	11am-12 pm	3876	4806	5168	5168	4962	4654	3812
Period	12-1 pm	3876	4806	5168	5168	4962	4602	3770

(52)								
Period (52)	1-2 pm	3876	4806	5168	5168	4962	4500	3686
Period (52)	2-3 pm	3794	4706	5060	5060	4856	4448	3644
Period (52)	3-4 pm	3836	4756	5114	5114	4910	4448	3644
Period (52)	4-5 pm	4040	5008	5386	5386	5170	4654	3812
Period (52)	5-6 pm	4080	5060	5440	5440	5222	5114	4188
Period (52)	6-7 pm	4080	5060	5440	5440	5222	5062	4146
Period (52)	7-8 pm	3916	4856	5222	5222	5014	4960	4064
Period (52)	8-9 pm	3712	4604	4950	4950	4752	4806	3938
Period (52)	9-10 pm	3386	4200	4516	4516	4334	4704	3854
Period (52)	10-11 pm	2978	3694	3972	3972	3812	4448	3644
Period (52)	11pm-12am	2570	3188	3428	3428	3290	4142	3392
